# Analysis of miR-497/195 cluster identifies new therapeutic targets in cervical cancer

**DOI:** 10.1186/s13104-024-06876-8

**Published:** 2024-08-02

**Authors:** Shreyas Hulusemane Karunakara, Sangavi Eswaran, Sandeep Mallya, Padmanaban S. Suresh, Sanjiban Chakrabarty, Shama Prasada Kabekkodu

**Affiliations:** 1 Department of Molecular Biology, Yuvaraja’s College, University of Mysore, Mysuru, Karnataka 570005 India; 2 Department of Cell and Molecular Biology, Manipal School of Life Sciences, Manipal Academy of Higher Education, Manipal, Karnataka 576104 India; 3 Department of Bioinformatics, Manipal School of Life Sciences, Manipal Academy of Higher Education, Manipal, Karnataka 576104 India; 4 School of Biotechnology, National Institute of Technology, Calicut, Kerala 673601 India

**Keywords:** miR-497/195, Bioinformatics, Prognosis, TCGA-CESC, Hub genes, Cervical carcinoma

## Abstract

**Objective:**

miR-497/195, located at 17p13.1, is a highly conserved miRNA cluster whose abnormal expression is a key regulator of carcinogenesis. We performed a comprehensive analysis of the miR-497/195 cluster to determine its prognostic utility and role in cervical cancer (CC) using publicly available datasets.

**Results:**

In silico analysis and validation revealed that this cluster is downregulated in CC. A total of 60 target genes of miR-497/195 cluster were identified as differentially expressed between normal and CC samples. ShinyGO, STRING, CytoHubba, Timer 2.0, HPA, and HCMBD were used for functional enrichment, PPIN network construction, hub gene identification, immune infiltration correlation, histopathological expression, and determination of the metastatic potential of miR-497/195 cluster and their target genes. PPIN analysis identified *CCNE1, CCNE2, ANLN, RACGAP1, KIF23, CHEK1, CDC25A, E2F7, CDK1,* and *CEP55* as the top 10 hub genes (HGs). Furthermore, the upregulation of RECK, ATD5, and BCL2, downregulation of OSBPL3, RCAN3, and HIST1H3H effected overall survival of CC patients. We identified 6 targets (TFAP2A, CLSPN, RASEF, HIST1H3H, AKT3, and ITPR1) of miR-497/195 cluster to influence metastasis. In addition, 8 druggable genes and 38 potential drugs were also identified. Our study identified miR-497/195 cluster target genes and pathways that could be used for prognostic and therapeutic applications in CC.

**Supplementary Information:**

The online version contains supplementary material available at 10.1186/s13104-024-06876-8.

## Introduction

Cervical cancer (CC) remains the leading cancer among female populations in developing countries [1, 2]. In 2022, approximately 661,021 new cases and 348,189 deaths due to CC were reported [3]. Multiple risk factors, such as human papillomavirus (HPV) infection, exposure to tobacco-based products, use of contraceptives, and unhealthy sexual habits, drive CC mortality [1, 4]. Previous genome-wide and mechanistic studies have indicated the role of abnormal genetic and epigenetic changes in CC pathology [5, 6]. The high mortality rate in CC and late detection suggests the need for epigenetic/genetic biomarkers that can be employed for the diagnosis and prognosis of CC [7].

The miR-497/195 cluster located at chromosome 17p13.1 encodes miR-497-5p and miR-195-5p in humans [8]. Clinical and functional studies revealed that this cluster is underexpressed in breast cancer [8], colorectal cancer [9], hepatocellular carcinoma [10], pancreatic cancer [11], ovarian cancer [12], and lymphomas [13]. However, gliomas and chronic lymphocytic leukemia (CLL) exhibit significant overexpression of miR-497, suggesting a possible oncogenic role [14]. In CC, serum miR-497 and miR-195 are proposed as biomarkers to distinguish CC and cervical intraepithelial neoplasia (CIN) patients from healthy individuals [15].

Genome-wide studies are invaluable data for studying molecular events during cancer development and progression [6, 16]. Previous studies have shown that the reanalysis of genome-wide data available in the public domain can identify markers for the clinical management of cancers [17]. The reanalysis of big data via an integrated systems biology approach and validation has been shown to identify molecular markers with high sensitivity and specificity for the diagnosis and prognosis of CC [18, 19]. We and others have demonstrated that the reanalysis of genome-wide studies may identify novel genes and pathways for the clinical management of CC [20, 21]. However, such studies related to miRNA clusters are limited in CC.

The current study aimed to investigate the diagnostic, prognostic, and functional role of the miR-497/195 cluster in CC using an integrated systems biology approach. Most previous studies focused on the functional investigation of individual miRNAs rather than the entire cluster. The advantages of studying the miR-497/195 cluster include a comprehensive understanding of the coordinated regulation and functional interplay between these miRNAs, offering deeper insights into cancer development. Further, this approach could reveal synergistic effects between miRNAs, uncover novel regulatory networks, and identify effective therapeutic targets compared to studying single miRNAs. We initially analysed cluster expression in normal and CC tissue samples from the Gene Expression Omnibus (GEO) and The Cancer Genome Atlas-Cervical Squamous Cell Carcinoma and Endocervical Adenocarcinoma (TCGA-CESC) datasets and subsequently validated the results using a previous study published by us [22]. Next, the target gene interactomes specific to the miR-497/195 cluster were constructed, and their clinical significance was characterized through functional enrichment analyses. Finally, we identified potentially druggable genes targetted by miR-497/195 cluster.

## Materials and methods

### Data source and identification of the expression of miR-497/195 cluster

The gene expression values of miR-497-5p and miR-195-5p were obtained from the TCGA-CESC and GEO portal (http://www.ncbi.nlm.nih.gov/gds/). The TCGA-CESC dataset consisted of 3 normal and 306 tumor samples. The GEO included the GSE55478 [23] and GSE86100 [24] datasets, which consisted of 10 normal and 10 tumor samples. Differential expression analysis of the clusters in the TCGA-CESC dataset was performed using RNA-seq data (Supplementary Material 2) from UCSC-Xena (https://xenabrowser.net/) [25]. Datasets obtained from GEO were queried using their accession IDs in the GEO portal. The resulting sample data (GSM) obtained were assigned to normal and tumor categories to determine the differential expression of the miR-497/195 cluster using the GEO2R tool. The differentially expressed miRNAs were considered statistically significant if p < 0.05.

### Validation of miR-497/195 cluster expression

The findings of the miR-497-5p and miR-195-5p expression were cross-validated using small RNA sequencing of normal (n = 15) and tumor (n = 15) cervical tissue samples published previously by us [22]. To further support our data, we performed a literature search to identify the interaction of miR-497-5p and miR-195-5p with target genes identified in the study.

The materials and methods used in the study are available in Supplementary Material 1.

## Results

### The miR-497/195 cluster is downregulated in CC

**Fig. 1:**
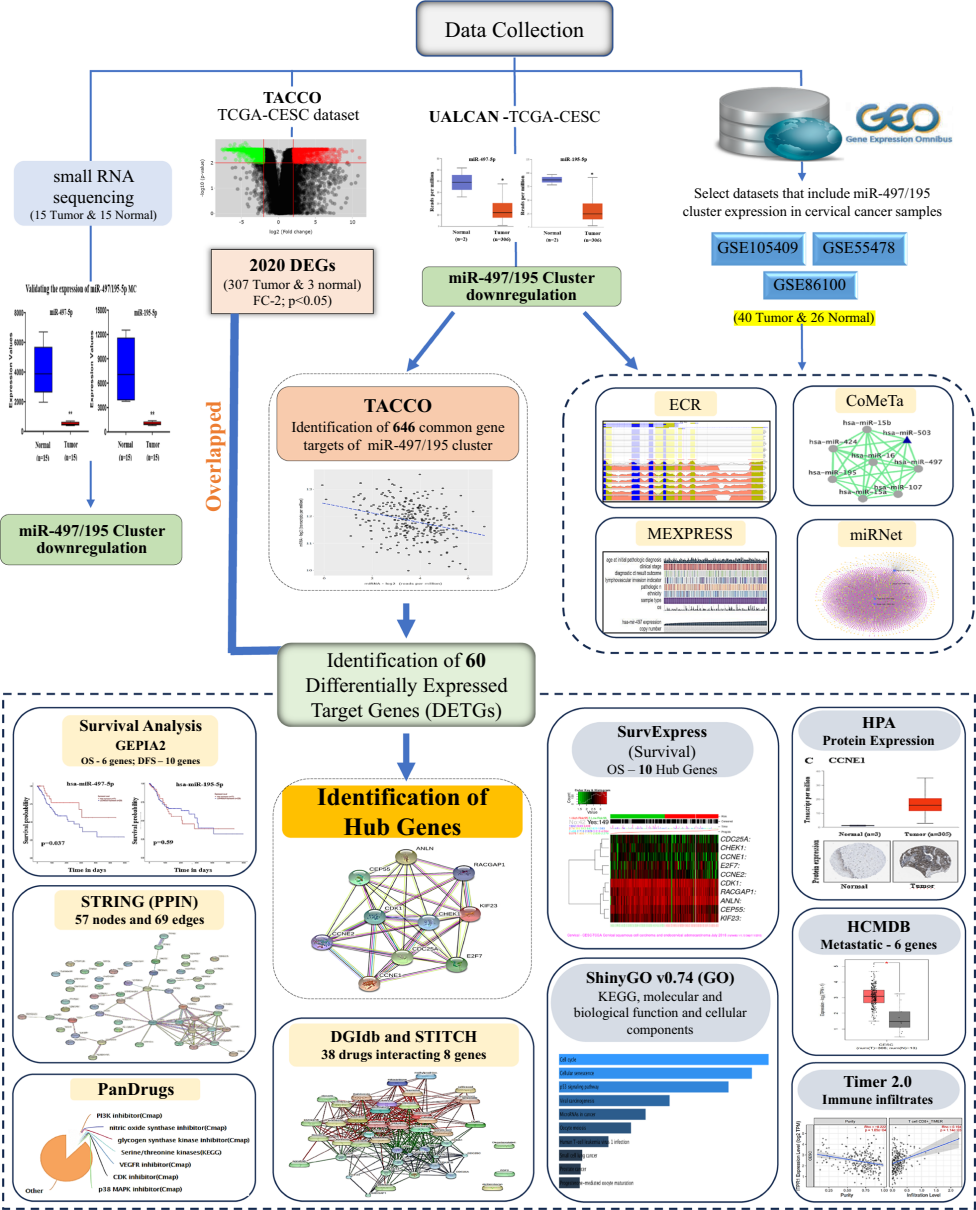
Schematic representation of workflow of in silico analysis

**Fig. 2:**
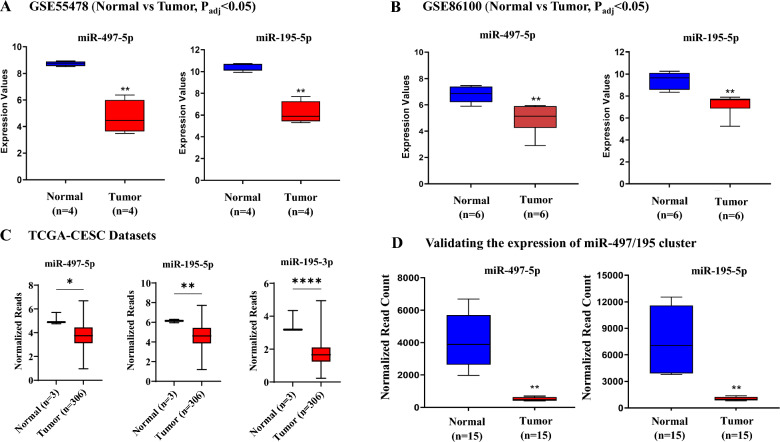
Expression analysis of hsa-miR-497-5p and hsa-miR-195-5p in the datasets. **A** and **B** Differentially downregulated miR-497-5p and miR-195-5p (normal vs tumor) in the GSE55478 and GSE86100 datasets, respectively. **C** Differential expression of the miR-497/miR-195 cluster in the TCGA-CESC dataset. **D** Validation of the expression of miR-497-5p and miR-195-5p via small RNA sequencing revealed the downregulation of both miRNAs in CC samples

The overall workflow of the study is represented in Fig. 1. The data retrieved from the GEO and TCGA-CESC datasets were evaluated for differential expression of miR-497-5p and miR-195-5p in CC. miR-497-5p and miR-195-5p were significantly downregulated in the GSE55478, GSE84100, and TCGA-CESC datasets (Fig. 2A–C). The cluster downregulation was further confirmed by analysing the expression of the miR-497/195 cluster from our previously published small RNA sequencing data (Fig. 2D) as tabulated in Supplementary Table 1. Supplementary Fig. 1 summarizes the correlation between the cluster and clinicopathological data. The cluster was found to be evolutionarily conserved across vertebrate genomes (Supplementary Fig. 2A). Interestingly, the downregulation of the cluster was significantly correlated with features such as computed tomography (CT) score, pathology, ethnicity, and sample type (Supplementary Fig. 2B and C).

### Construction of the miR-497/195 cluster and identification of target genes

**Fig. 3:**
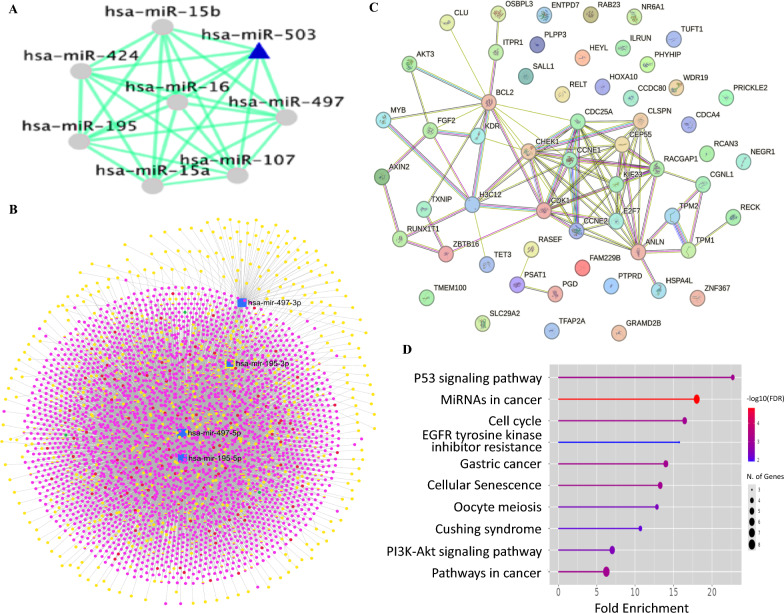
The network and interactome of miR-497/195 cluster. **A** miRNA‒miRNA interactions as predicted by CoMeTa. **B** Represents the interactome of miR-497/195 cluster (Blue) and its target genes (Yellow), lncRNAs (Red), sncRNAs (Green), and circRNAs (Pink) as identified by miRNet. **C** Represents the PPIN of 60 DETGs that are targeted by miR-497/195 cluster displaying 57 nodes and 69 edges. **D** Represents the pathway enrichment analysis of miR-497/195 cluster target genes

CoMeTa tool was used to identify miR-15b, miR-15a, miR-16, miR-195, miR-107, miR-424, and miR-503 as potential coexpressed miRNAs (Fig. 3A). Figure 3B displays the interactome of the miR-497/195 cluster (shown in blue) with its target genes (yellow), lncRNAs (red), sncRNAs (green), and circRNAs (pink), as identified by miRNet (3930 nodes). Analysis of the TCGA-CESC dataset using TACCO database revealed 2020 DEGs (802 upregulated and 1218 downregulated; ± twofold, p < 0.05) (Supplementary Table 2). Among these, 646 genes were identified as targets of the miR-497/195 cluster (Supplementary Table 3). Furthermore, the overlapping analysis identified 60 differentially expressed target genes (DETGs) in CC (Supplementary Table 4). Among the 60 DETGs, 27 were upregulated and 26 were downregulated targets of miR-497-5p, whereas 22 were upregulated and 25 were downregulated targets of miR-195-5p in CC. Furthermore, 40 DETGs were identified as common targets for both miRNAs (18 upregulated and 22 downregulated) (Supplementary Table 4).

### PPIN and functional enrichment analysis

The PPIN of the 60 DETGs comprised 57 nodes, 69 edges, and an interaction enrichment with a p value = 5.94e-13 (Fig. 3C). Pathway enrichment analyses of DETGs revealed that the p53 signaling pathway, microRNAs involved in cancer, the cell cycle, EGFR tyrosine kinase inhibitor resistance, gastric cancer, cellular senescence, oocyte meiosis, and Cushing syndrome were the top 10 enriched KEGG pathways (Fig. 3D). Gene Ontology for biological process enrichment included regulation of retinal cell programmed cell death, assembly of the actomyosin apparatus involved in cytokinesis, actomyosin contractile ring assembly, positive regulation of cell migration involved in sprouting angiogenesis, mitotic cytokinesis, embryonic limb morphogenesis, embryonic appendage morphogenesis, sprouting angiogenesis, appendage morphogenesis, and limb morphogenesis (Supplementary Fig. 3A). Cell component enrichment included cyclin E1-CDK2 complex, central spindlin complex, muscle thin filament tropomyosin, cyclin B1-CDK2 complex, cyclin E2-CDK2 complex, bleb, perinuclear endoplasmic reticulum, Flemming body, striated muscle thin filament, and myofilament (Supplementary Fig. 3B).

Similarly, the enriched molecular functions included histone kinase activity, structural constituent of muscle, DNA-binding transcription repressor activity-RNA polymerase II specific, DNA-binding transcription repressor activity, DNA-binding transcription activator activity-RNA polymerase II specific, DNA-binding transcription activator activity, and identical protein binding (Supplementary Fig. 3C).

### Prognostic significance of miR-497/195 cluster in CC

**Fig. 4:**
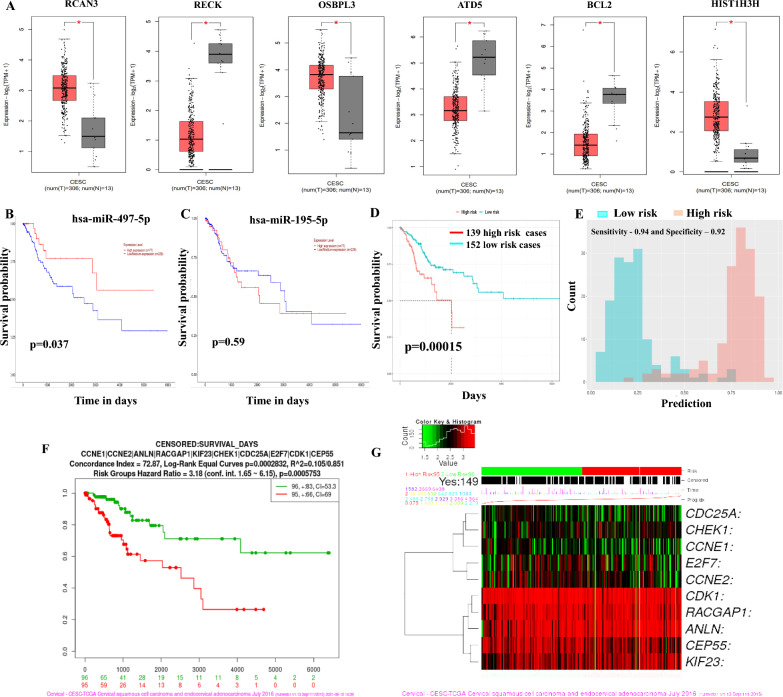
Identification of survival-significant genes. **A** Differential expression of 6 genes associated with overall survival. **B** and **C** Kaplan‒Meier survival plot of miR-497-5p (significant) and miR-195-5p (nonsignificant). **D** The survival probability of 60 DETGs for high-risk (red line) and low-risk (blue line) patients. **E** The sensitivity and specificity of the prognostic model predicted by the random forest method. **F** OS predicted for Hub Genes by SurvExpress. **G** Heatmap of Hub gene expression in CC

The analysis of the 60 DETGs in CC revealed 6 prognostically potential genes (RCAN3, RECK, OSBPL3, ATD5, BCL2, and HIST1H3H) (Fig. 4A). The altered expression of these genes affected the OS of CC patients (Supplementary Fig. 4A). The downregulation of miR-497-5p but not miR-195-5p affected overall survival (Fig. 4B, C). Additionally, 10 DETGs (RECK, HOXA10, PTPRD, KDR, TPM1, MYB, AXIN2, NEGR1, TMEM100, and SLC229A2) were associated with DFS in CC patients. Using the Random Forest model, the OS of DETGs predicted 139 high-risk and 152 low-risk CC with a specificity and sensitivity of 0.92 and 0.94, respectively (Fig. 4D, E). Additionally, the DFS of DETGs identified 150 high-risk and 138 low-risk CC with a specificity of 0.88 and a sensitivity of 0.94 (Supplementary Figs. 5B and C). Furthermore, the model predicted N-stage: N0 (138 entries) and N1, N2, N3 (55 entries) with a specificity of 0.9 and a sensitivity of 0.99 (Supplementary Fig. 5D). Similarly, the model predicted T-stage: T1, T2 (217 entries) and T3, T4 (25 entries) with a specificity of 0.81 and a sensitivity of 1 (Supplementary Fig. 5E).

### Identification of DETGs associated with metastasis

Next, we evaluated the associations between the DETGs and metastatic potential. Among the 60 DETGs, genes such as TFAP2A, CLSPN, RASEF, HIST1H3H, AKT3, and ITPR1 were associated with metastasis to secondary sites, including the lungs, and with head and neck carcinoma (Supplementary Table 5). The expression of metastatic genes and their association with OS were verified using GEPIA2 (Supplementary Fig. 5). Interestingly, we found that HIST1H3H has significant prognostic value for OS and metastasis in CC patients.

### Identification of Hub genes and functional enrichment analysis

The top 10 highly connected genes identified in our in silico analysis included *CCNE1, CCNE2, ANLN, RACGAP1, KIF23, CHEK1, CDC25A, E2F7, CDK1,* and *CEP55* (Supplementary Fig. 6A). Among that, 9 HGs were experimentally validated targets for miR 497/195 cluster identified using MirTarBase (Supplementary Table 6). Pathway enrichment analysis of HGs using Enrichr (https://maayanlab.cloud/Enrichr/) against the KEGG pathway revealed cell cycle regulation, cellular senescence, the p53 signalling pathway, viral carcinogenesis, regulation of miRNAs in cancer, and other functions as enriched (Supplementary Fig. 6B). The BP, Cell Comp, and MF enrichment of the HG-related genes are provided in Supplementary Fig. 6C–E. Briefly, the enriched BPs included mitotic cytokinesis, protein kinase activity, G1/S-cell cycle transition, mitotic spindle mid-zone assembly, and regulation of cytokinesis. Enriched MFs included cyclin-dependent serine/threonine kinase regulation, histone-threonine kinase activity, microtube binding, RNA polymerase II CTD heptapeptide kinase activity, and tubulin activity. The enriched Cell Comp were cyclin-dependent kinase holoenzyme complex, serine-threonine protein kinase complex, mitotic spindle, nucleus, bleb, microtubules, intracellular membrane, cell-cortex region, and condensed nuclear chromosome.

### HG expression and its prognostic application

**Fig. 5:**
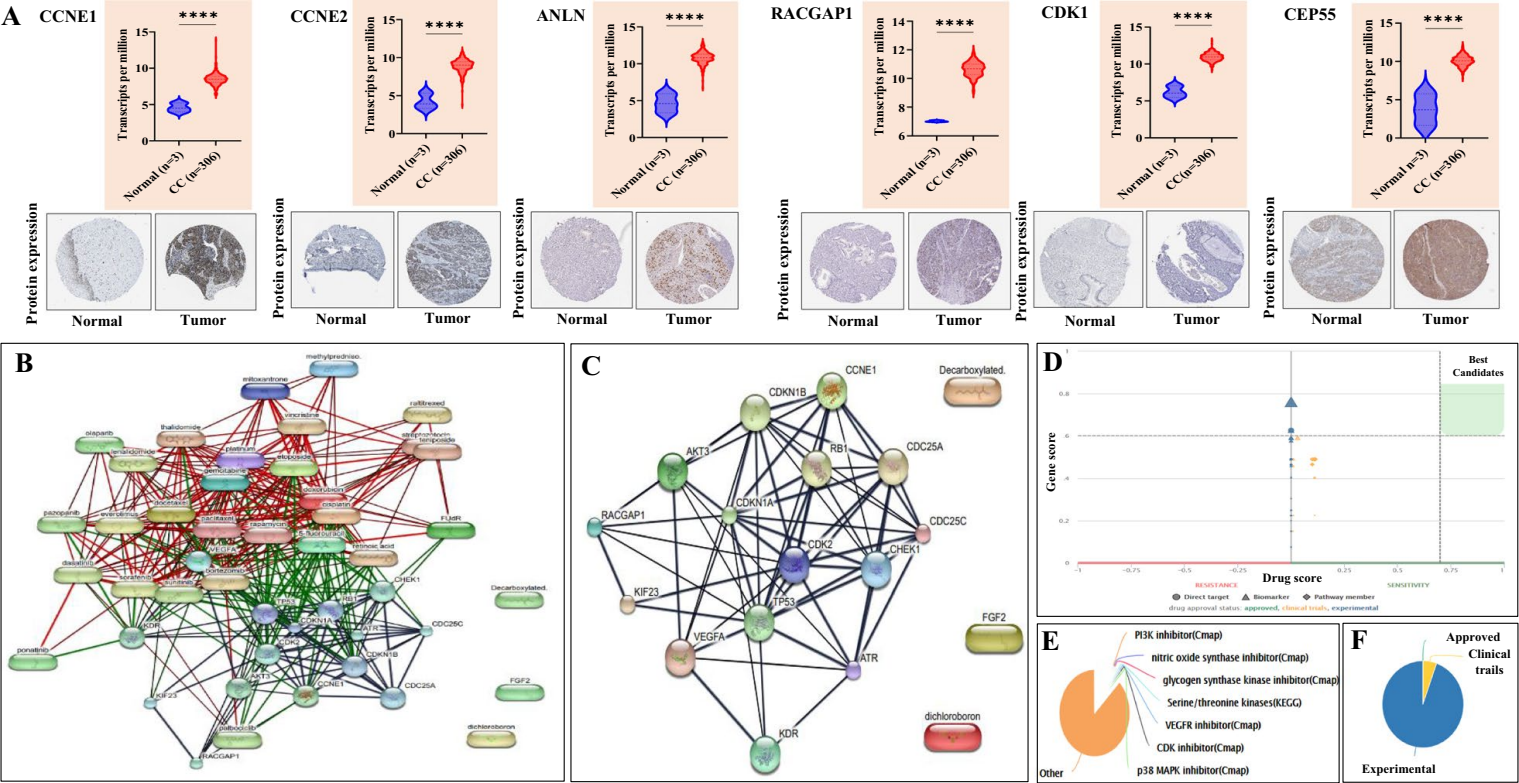
Identification of protein expression and drug-gene interaction network analysis. **A** Differential expression of Hub genes at the mRNA level is represented as violin plots, whereas the protein levels are represented as immunohistochemical images. **B** STITCH analysis depicting the interactions between the 8 target genes and 38 drugs. **C** STITCH analysis showing the drug-gene interactions of the 10 hub genes. **D** Gene-score vs. drug-score graph showing the status of drug regimens interacting with 8 target genes. **E** Classification of drug-target genes interacting by families. **F** Pie chart showing the drug approval status

SurvExpress analysis revealed that the expression of the 10 HGs can affect the OS of patients (p = 0.0005753 and hazard ratio = 3.18) (Fig. 4F, G). The expression of HGs at the protein level in normal and tumor samples was analysed with the HPA tool. Among the 10 HGs, protein expression data were available for only 6 genes (CCNE1, CCNE2, ANLN, RACGAP1, CDK1, and CEP55). The protein expression data were in concordance with the TCGA-CESC hub gene expression data (Fig. 5A). Furthermore, analysis of the role of HGs in immune infiltration using TIMER 2.0 revealed that abnormal expression of HGs may predict CD4 + T cell, CD8 + T cell, and neutrophil infiltration in CC (Supplementary Fig. 7 and Supplementary Table 7).

### Identification of drug–gene interactions

Drug-gene interaction analysis of 60 DETGs in the cluster revealed 38 drugs that interact with 8 target genes, namely, KDR, MYB, CCNE1, CHEK1, RACGAP1, AKT3, BCl2, and FGF2 (Supplementary Table 8). STITCH network analysis of all 8 target genes and interacting drugs revealed a network of 15 nodes and 64 edges (Fig. 5B). The 10 HGs, along with their interacting drug partners, provided 10 nodes and 22 edges (Fig. 5C). We found that few genes were targeted by multiple drugs, such as BCL2 (cisplatin, paclitaxel, etoposide, vincristine, and doxorubicin), CHEK1 (cisplatin, gemcitabine, etoposide, and palbociclib), and MYB (paclitaxel and doxorubicin). Among these drugs, only the gemcitabine-cisplatin combination was found to be approved for CC treatment. Consistent with this observation, PanDrugs analysis identified other potential drugs targeting these genes as either experimental drugs or drugs under clinical trials (Fig. 5D–F).

## Discussion

Despite advancements in early detection and availability of the HPV vaccine, CC is a leading gynecological cancer in many underdeveloped and developing countries [2, 4]. The high incidence and mortality of CC may be due to (i) a lack of active implementation of cancer screening programs for early detection, (ii) knowledge, attitudes, and practices related to CC, and (iii) molecular determinants responsible for CC [7]. The availability of genome-wide data in the public domain and advances in the field of bioinformatics have provided an opportunity to reanalyze these data to understand the critical genes and pathways responsible for carcinogenesis and to translate the findings for improved management of cancer patients.

MicroRNAs are a class of noncoding RNAs critical for regulating the expression of protein-coding genes. Several reports have shown that abnormal expression of miRNAs is a critical event during CC [22, 26]. Furthermore, profiling the abnormally expressed miRNAs can be used for diagnostic and prognostic applications in CC. Our previous study demonstrated that integrated bioinformatic analysis can identify miRNAs as diagnostic and prognostic biomarkers and revealed their role in disease-causing mechanisms in conditions such as cancers [19].

Our study showed that miR-497-5p and miR-195-5p were downregulated in CC and may promote cervical carcinogenesis by targeting cellular senescence, the p53 signaling pathway, the cell cycle, and EGFR tyrosine kinase inhibitor resistance. The downregulation of miR-497-5p expression correlated with poor OS. Our in silico analysis using three CC datasets followed by experimental validation from our small RNA-seq data confirmed previous findings that suggested the tumor-suppressor function of the cluster in CC [27–29]. However, HCMDB analysis revealed that miR-497/195 cluster expression was not significantly altered between metastatic and nonmetastatic CC. Thus, our in silico analysis identified several known and novel miR-497/195 cluster interactomes with diagnostic and prognostic potential in CC.

A study by Zhang et al. 2015 demonstrated that circulating miR-16–2*, miR-195, miR-2861, and miR-497 can be useful in distinguishing normal from cervical intraepithelial neoplasia (CIN) and CC [15]. This finding suggested that measuring the circulating levels of miRNAs in the cluster might be a reliable diagnostic tool. Although miR-195 and miR-497 are coexpressed, most studies have investigated their functions individually rather than as clusters. For example, a study by Chen and coworkers confirmed that miR-497-5p inhibits CC proliferation by inducing cell cycle arrest by targeting the CBX4 gene [27]. Previous studies using clinical samples and cell lines have reported the tumor growth suppressive functions of the miR-497/195 cluster in CC. In SiHa and HeLa cells, overexpression of miR-497 inhibited the growth, invasion, and migration of cells by activating caspase-mediated apoptosis and inhibiting the insulin-like growth factor 1 receptor [30]. Similarly, miR-497 is known to regulate the expression of FASN [31] and CBX4 [27] in CC. It has also been reported that the HPV-encoded E6 oncoprotein can target the KDM5C/lnc_000231/miR-497-5p/CCNE1 axis to stimulate CC progression [32]. Our in silico analysis of the miR-497/195 cluster interactome also identified CCNE1 as one of the HGs.

miR-195-5p is another downregulated member of the miR-497/195 cluster in CC. miR-195-5p targets MMP14 to suppress CC proliferation and invasion by inhibiting TNF signaling pathways [33]. Furthermore, miR-195-5p prevents malignant progression by inhibiting ATG9A [34] and YAP1-mediated EMT in CC [29]. Furthermore, modulation of the miR-195-5p/MAPK axis contributed to the proliferation and migration of CC cells [32]. Taken together, various functional studies indicate that the downregulation of miR-195-5p and miR-497-5p fuels the growth, migration, proliferation, and invasion of CC cells.

Our study identified 60 differentially expressed target genes and a subclass of hub genes whose upregulation predicted CC prognosis and metastasis. Our risk prediction model using the random forest approach successfully revealed that the 60 DETGs of the miR-497/195 cluster could differentiate 134 high-risk and 136 low-risk individuals with a test specificity of 0.92 and sensitivity of 0.94, suggesting that the DETGs could have a powerful prognostic function in determining high/low-risk CC. Among the 60 DETGs in the cluster, RCAN3, RECK, OSBPL3, ATD5, BCL2, and HIST1H3H affected the OS of CC patients, suggesting the prognostic significance of these genes in CC. Furthermore, analysis of the PPIN identified CCNE1, CCNE2, ANLN, RACGAP1, KIF23, CHEK1, CDC25A, E2F7, CDK1, and CEP55 as the key HGs. All the HGs were significantly upregulated in CC samples in the TCGA-CESC cohort. Notably, the overexpression of a few HGs was associated with OS and DFS as well as the induction of metastatic phenotypes. KM survival analysis revealed that the overexpression of HGs can significantly affect OS among high-risk and low-risk CC patients, with patients with lower HG expression exhibiting better survival than patients with higher HG expression. Thus, testing the expression of the miR-497/195 cluster and its HGs may be useful for predicting the diagnosis, prognosis, and metastasis of CC patients.

To further support our data, we performed a literature search to identify the interaction of miR-497-5p and miR-195-5p with target genes identified in the study. For example, targeting of CCNE1 by miR-497 to suppress cervical cancer cell proliferation is reported [32]. An indirect association between miR-497and CDC25A mediated by LNC00662 is reported by Wei et al. 2020 [35]. Likewise, a direct interaction between miR-195-5p and Clusterin (CLU) is reported in prostate cancer cells through luciferase reporter assay [36]. Similarly targeting of HOXA10 by miR-195-5p and an inverse relationship between them is reported in lung adenocarcinoma [37]. miR-195-5p is shown to regulate AXIN2 in colorectal cancer cells [38]. BCL2 targeting by miR-497 in A549 is reported in lung adenocarcinoma cells [39]. miR-195/ FGF axis and miR-195 as a negative regulator of FGF are reported in prostate cancer cells [40], hepatocellular carcinoma [41], and thyroid cancer [42]. We found a study that reported overexpression of miR-195 inhibits cervical cancer progression by targeting CCND1 and MYB [43]. While few studies support our target predictions with experimental validation, some of the DETGs identified in our study lack validation in cervical cancer. Collectively, our data suggests that miR-497/195 has the potential to target DETGs and requires further validation.

Resistance to therapy is a major concern in cancer treatment, and recurrence affects the quality of life of individuals [44, 45]. Thus, understanding the molecular mechanisms leading to drug resistance is critical for improving the management of CC patients. To identify druggable genes and repurpose drugs, we performed a drug‒gene interaction analysis and identified 38 potential drugs targeting 8 genes. Although several of these drugs, except for the gemcitabine-cisplatin combination, are approved for CC management, the nonconventional drugs identified either as experimental candidates or in clinical trials serve as targets for drug repurposing for better therapeutic outcomes in CC. Our study collectively identified the clinical relevance and significance of the miR-497/195 cluster and its target network in terms of its diagnostic and prognostic applications using an array of statistical tests and bioinformatic predictions, offering novel insights for developing functional studies.

## Limitations

• An independent validation of the miR-497/195 cluster was performed with only 15 normal and 15 tumor samples, and the TCGA CESC dataset included only 3 normal samples. Though these samples were able to capture the differences in cluster expressions, a more detailed investigation using a large number of clinical samples is needed before further conclusions are drawn.
• Our study used public datasets and web-based tools to perform the analysis. Although we used only those gene targets that were experimentally validated through different independent studies from target identification databases, many other target genes might be targeted by this miRNA cluster which might need further functional validations.
• While this study provides comprehensive details, limitations about the in-silico nature of the study should be considered by the scientific community to conduct specific studies to validate and confirm these results through further experimental validation.

### Supplementary Information

Additional file 1: Figure 1. miR-497 and miR-195 expression analysis was performed using TCGA-UALCAN. A) Expression analysis of miR-497 in normal tissues and CC tissues at cancer stages 1, 2, and 3. B) Different age groups. C) Ethnicity and race. D) Tumor grade. E) Expression analysis of miR-195 in normal tissues and CC tissues at cancer stages 1, 2, and 3. F) Different age groups. G) Ethnicity and race and H) tumor grade.

Additional file 2: Figure 2. Conservation and expression analysis of miR-497/195 cluster. A) miR-497 and miR-195 comprise UTRs (yellow blocks) across species. B) & C) Correlation analysis of miR-497 and miR-195 expression from TCGA-CESC datasets and clinical attributes.

Additional file 3: Figure 3. Functional enrichment analysis of miR-497/195 cluster target genes. A) Biological Processes, B) Cellular Components, and C) Molecular Functions.

Additional file 4: Figure 4. The survival plots of genes associated with A) Overall Survival B)&C) Disease Free Survival and D)& E) Cancer stages.

Additional file 5: Figure 5. Box plots of six metastatic genes in CC.

Additional file 6: Figure 6. Identification and characterization of Hub genes. A) Identification of Hub genes using the STRING database and 10 interacting genes (CCNE1, CCNE2, ANLN, RACGAP1, KIF23, CHEK1, CDC25A, E2F7, CDK1, and CEP55). B) Functional enrichment analysis of the hub genes. B) KEGG pathway analysis. GO enrichment of the component Hub genes (C) Cellular component (D) Molecular functions and (E) Biological processes.

Additional file 7: Figure 7. miR-497/195 cluster and its immune infiltrates. Spearman infiltration levels of CD8+ T cells, CD4+ T cells, and neutrophils.

Additional file 8. Supplementary Material 1: The materials and methods used in the present study.

Additional file 9. Supplementary Material 2: miRNA and mRNA expression values in normal and tumor samples of the TCGA-CESC dataset from the UCSC-Xena Browser.

Additional file 10: Table 1. List of differentially expressed miRNA cluster and its members in CC from small RNA sequencing data.

Additional file 11: Table 2. List of differentially expressed genes in CC from the TCGA-CESC dataset.

Additional file 12: Table 3. List of genes targeted by miRNA-497/195 cluster.

Additional file 13: Table 4. List of differentially expressed target genes.

Additional file 14: Table 5. The differentially expressed target genes of miR-497/195 cluster and their characteristic metastatic signatures.

Additional file 15: Table 6. The mir497/195 cluster and its experimentally validated gene target interactions from miRTarBase database.

Additional file 16: Table 7. Hub Genes and Immune Infiltrates.

Additional file 17: Table 8. The list of drugs and interacting genes.

## Data Availability

All the data generated or analysed during this study are included in this published article and its supplementary files. The TCGA-CESC miRNA and mRNA data are included in the Additional Data. No datasets were generated or analysed during the current study.

## Abbreviations

NGS: Next-generation sequencing
PI3/Akt: Phosphatidylinositol 3-kinase/protein kinase B
EGFR: Epidermal growth factor receptor
DNA: Deoxyribonucleic acid
*CCNE1*: G1/S-specific cyclin-E1
*CCNE2*: G1/S-specific cyclin-E2
*ANLN*: Anillin actin binding protein
*RACGAP1*: Rac GTPase activating protein 1
*KIF23*: Kinesin family member 23
*CHEK1*: Checkpoint kinase1
*CDC25A*: Cell division cycle25a
*E2F7*: E2F Transcription factor 7
*CDK1*: Cyclin-dependent kinase1
*CEP55*: Centrosomal Protein 55
*RCAN3*: RCAN family member3 < *RCAN*-regulator of calcineurin >
*RECK*: Reversion-inducing cysteine-rich *protein* with Kazal motifs
*OSBPL3*: Oxysterol-binding protein-related protein 3
*ATD5*: Asphyxiating thoracic dystrophy 5
*BCL2*: B-cell lymphoma 2
HIST1H3H: Histone cluster 1 H3 family member H
*HOXA10*: Homeobox A10
