# Supplementary figures and images for: Analysis of miR-497/195 cluster identifies new therapeutic targets in cervical cancer
Source: BMC Res Notes. 2024 Aug 2;17:217. doi: 10.1186/s13104-024-06876-8 (PMC11297691; doi:10.1186/s13104-024-06876-8)

## Slide 1
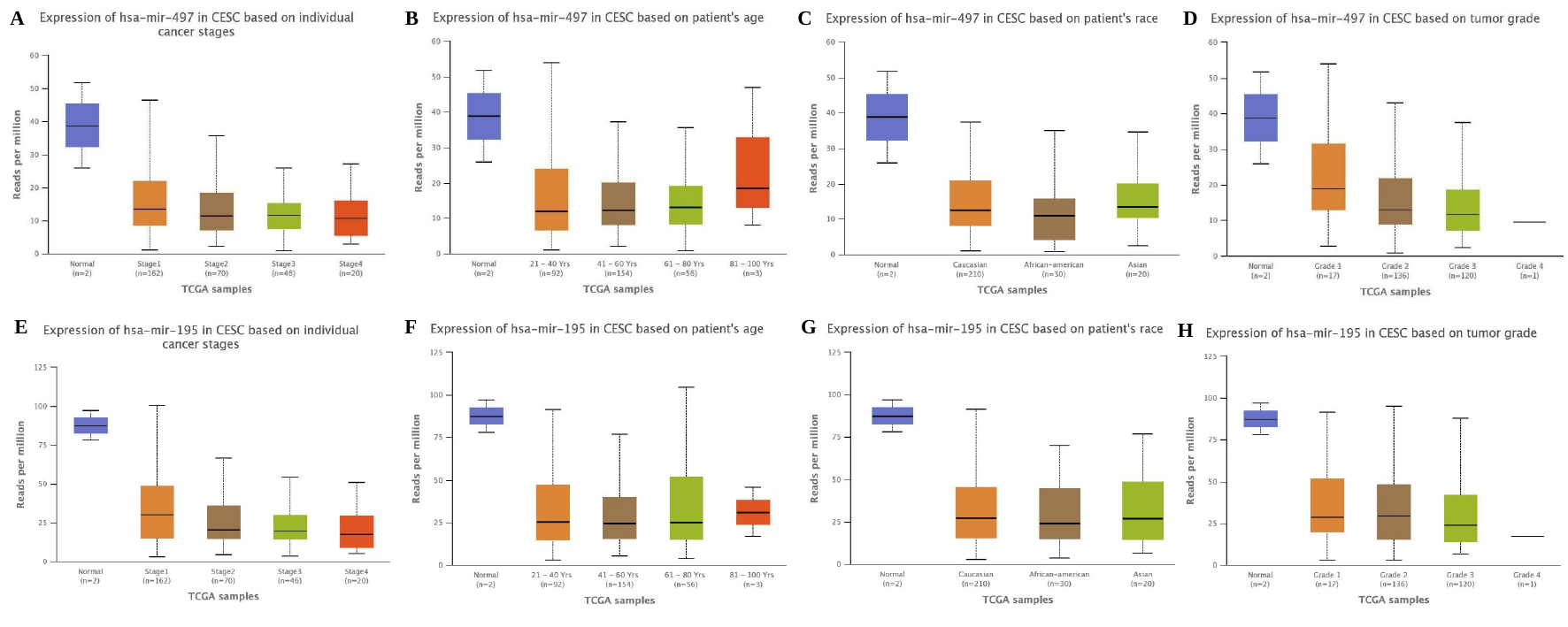

A
D
B
C
G
F
E
H

Supplement: Supplementary file 1 — Additional file 1: Figure 1. miR-497 and miR-195 expression analysis was performed using TCGA-UALCAN. A) Expression analysis of miR-497 in normal tissues and CC tissues at cancer stages 1, 2, and 3. B) Different age groups. C) Ethnicity and race. D) Tumor grade. E) Expression analysis of miR-195 in normal tissues and CC tissues at cancer stages 1, 2, and 3. F) Different age groups. G) Ethnicity and race and H) tumor grade. [file 13104_2024_6876_MOESM1_ESM.pptx]

## Slide 1
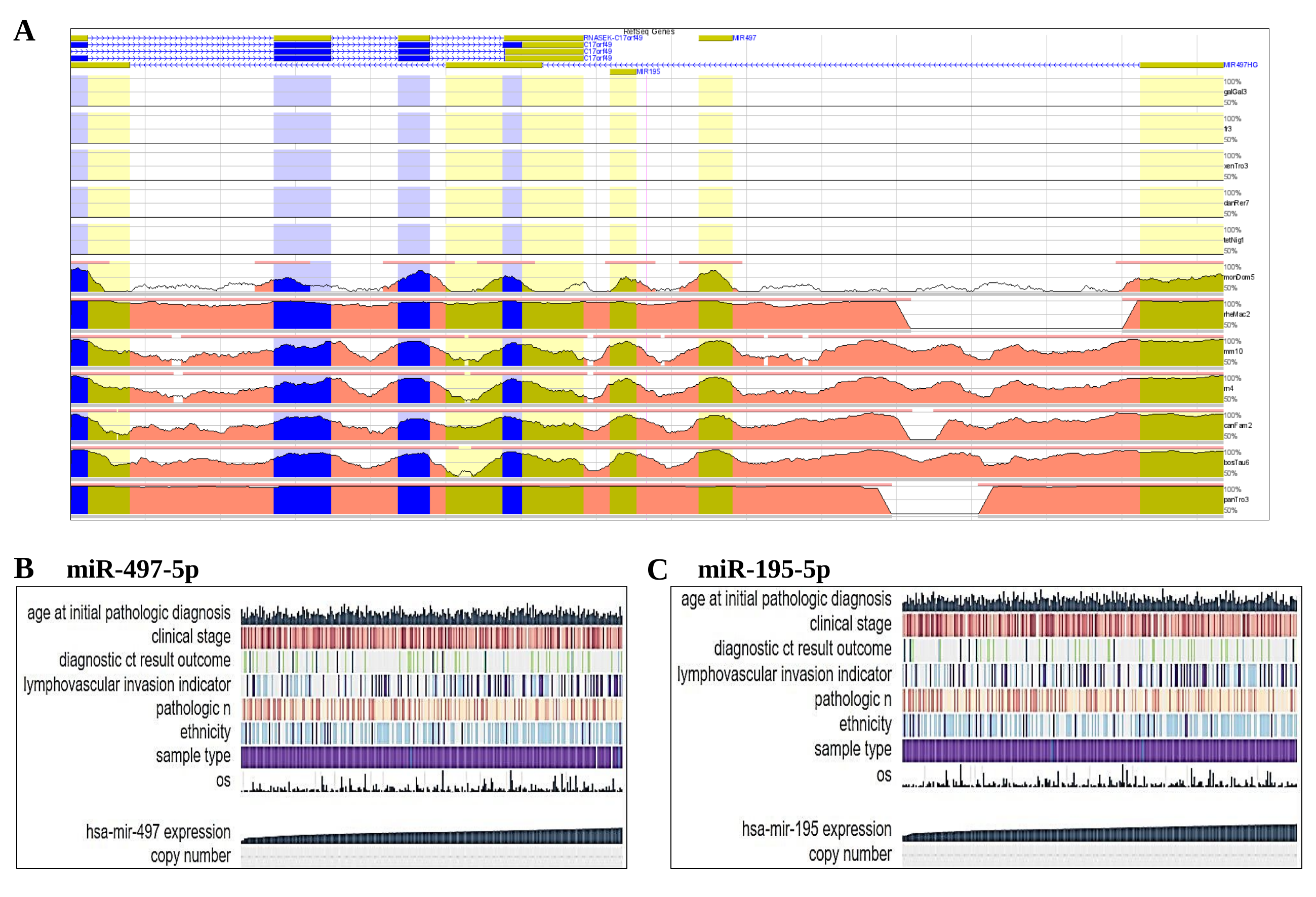

A
B
C
miR-497-5p
miR-195-5p

Supplement: Supplementary file 2 — Additional file 2: Figure 2. Conservation and expression analysis of miR-497/195 cluster. A) miR-497 and miR-195 comprise UTRs (yellow blocks) across species. B) & C) Correlation analysis of miR-497 and miR-195 expression from TCGA-CESC datasets and clinical attributes. [file 13104_2024_6876_MOESM2_ESM.pptx]

## Slide 1
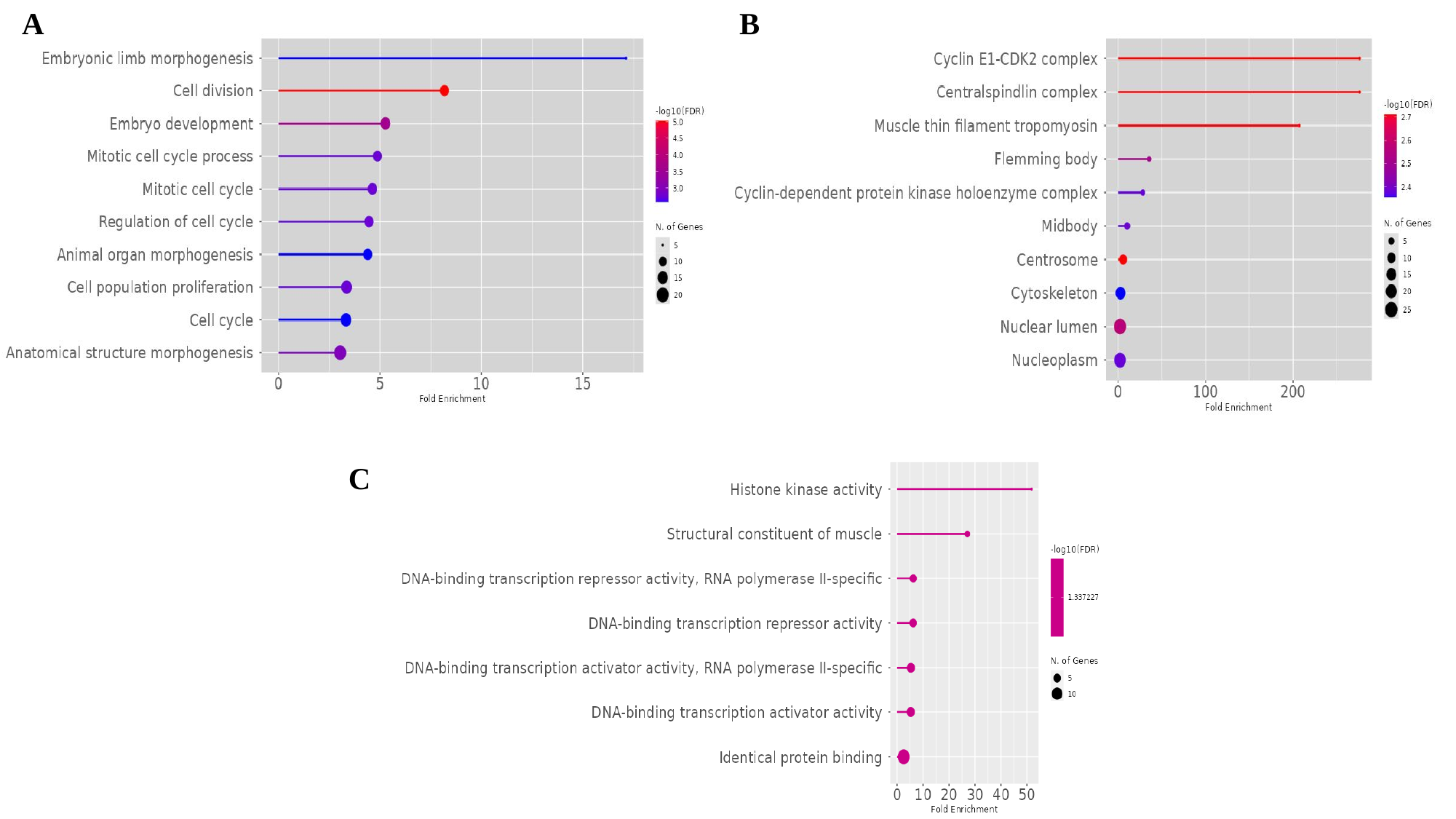

A
B
C

Supplement: Supplementary file 3 — Additional file 3: Figure 3. Functional enrichment analysis of miR-497/195 cluster target genes. A) Biological Processes, B) Cellular Components, and C) Molecular Functions. [file 13104_2024_6876_MOESM3_ESM.pptx]

## Slide 1
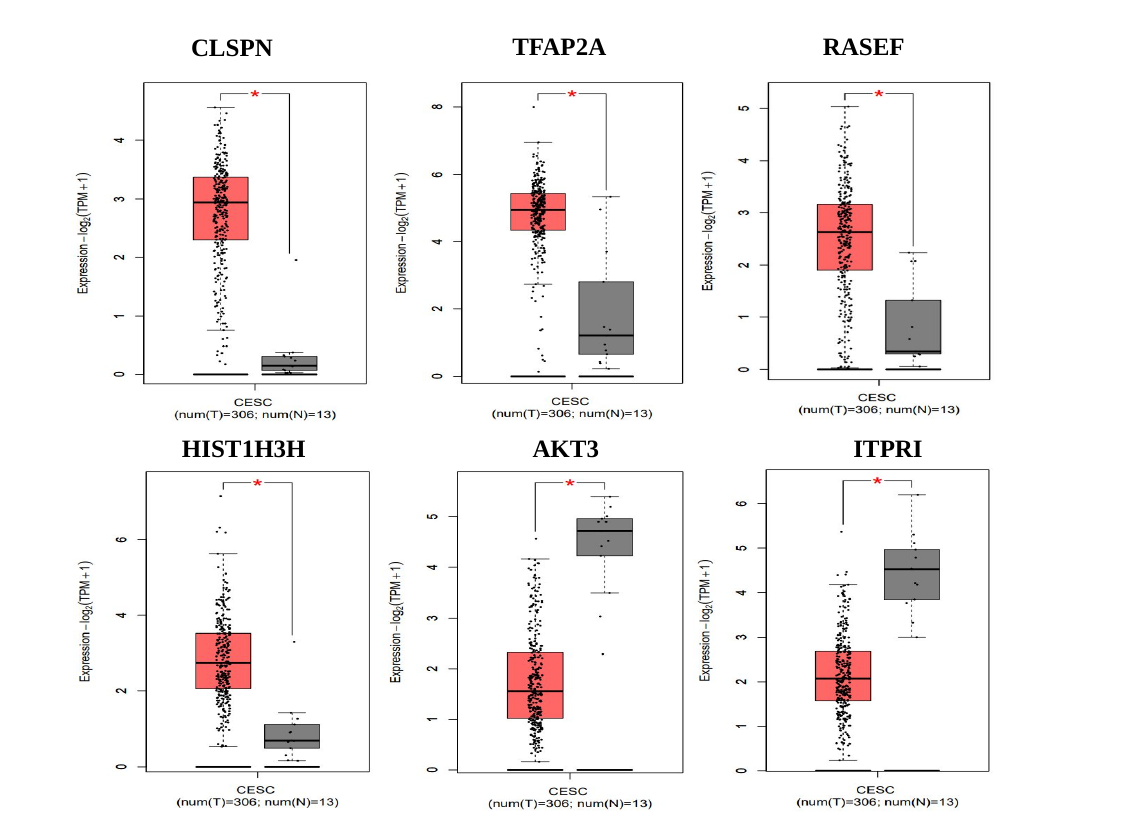

TFAP2A
RASEF
CLSPN
HIST1H3H
AKT3
ITPRI

Supplement: Supplementary file 5 — Additional file 5: Figure 5. Box plots of six metastatic genes in CC. [file 13104_2024_6876_MOESM5_ESM.pptx]

## Slide 1
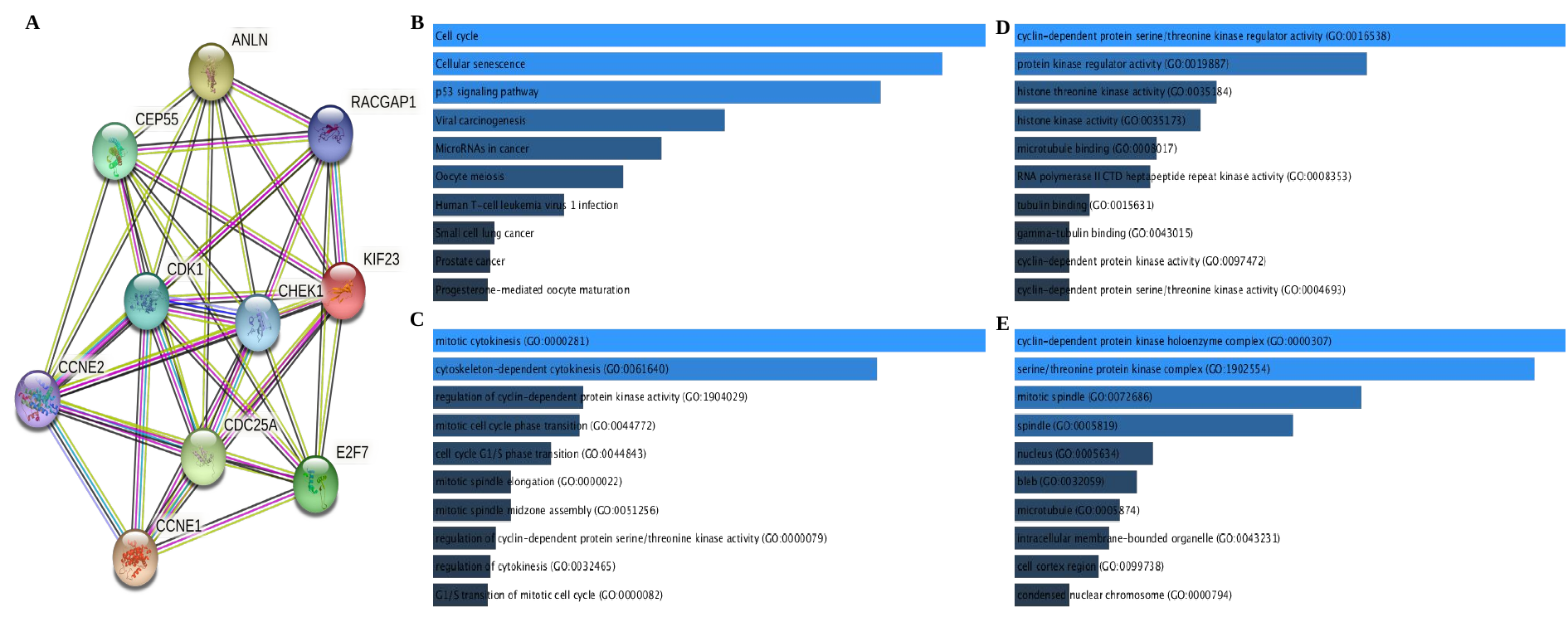

A
B
D
C
E

Supplement: Supplementary file 6 — Additional file 6: Figure 6. Identification and characterization of Hub genes. A) Identification of Hub genes using the STRING database and 10 interacting genes (CCNE1, CCNE2, ANLN, RACGAP1, KIF23, CHEK1, CDC25A, E2F7, CDK1, and CEP55). B) Functional enrichment analysis of the hub genes. B) KEGG pathway analysis. GO enrichment of the component Hub genes (C) Cellular component (D) Molecular functions and (E) Biological processes. [file 13104_2024_6876_MOESM6_ESM.pptx]

## Slide 1
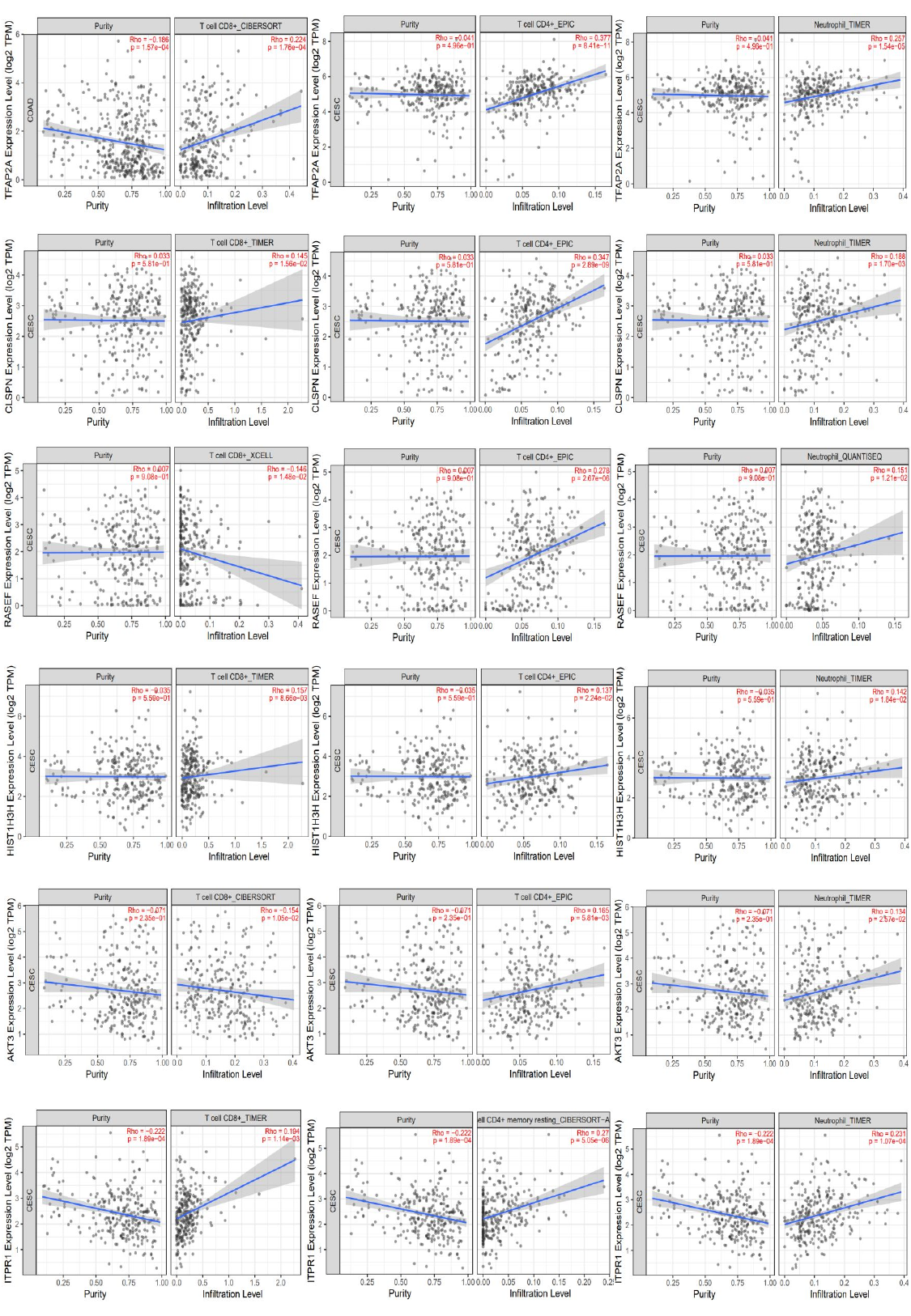

Supplement: Supplementary file 7 — Additional file 7: Figure 7. miR-497/195 cluster and its immune infiltrates. Spearman infiltration levels of CD8+ T cells, CD4+ T cells, and neutrophils. [file 13104_2024_6876_MOESM7_ESM.pptx]
