# Supplementary material for: Analysis of miR-497/195 cluster identifies new therapeutic targets in cervical cancer
Source: BMC Res Notes. 2024 Aug 2;17:217. doi: 10.1186/s13104-024-06876-8 (PMC11297691; doi:10.1186/s13104-024-06876-8)
Supplement: Supplementary file 4 — Additional file 4: Figure 4. The survival plots of genes associated with A) Overall Survival B)&C) Disease Free Survival and D)& E) Cancer stages. [file 13104_2024_6876_MOESM4_ESM.pptx]

## Slide 1
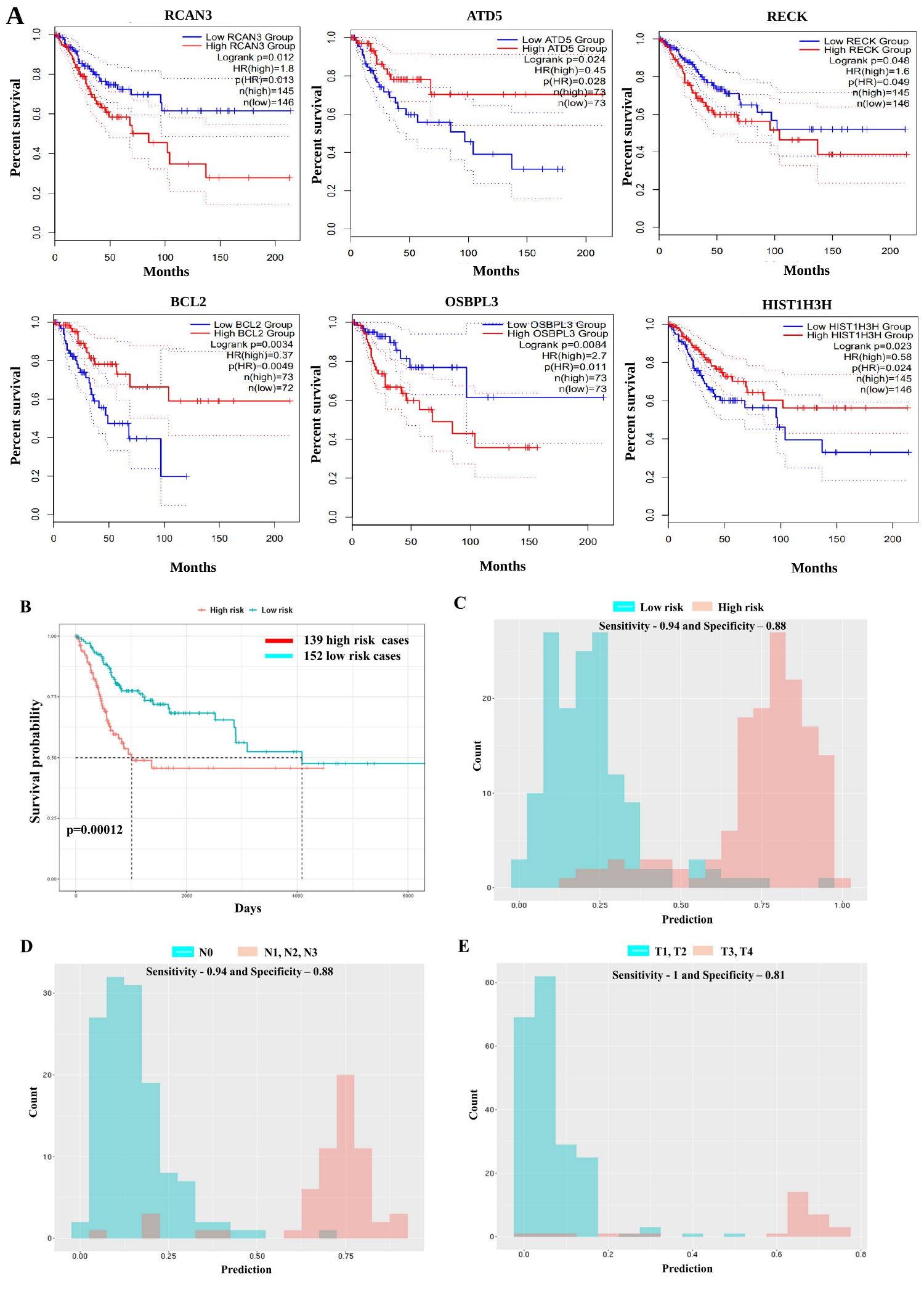

RCAN3
A
ATD5
RECK
Percent survival
Percent survival
Percent survival
HIST1H3H
Months
Months
Months
OSBPL3
BCL2
HIST1H3H
Percent survival
Percent survival
Percent survival
Months
Months
Months
