# Supplementary material for: Analysis of miR-497/195 cluster identifies new therapeutic targets in cervical cancer
Source: BMC Res Notes. 2024 Aug 2;17:217. doi: 10.1186/s13104-024-06876-8 (PMC11297691; doi:10.1186/s13104-024-06876-8)
Supplement: Supplementary file 8 — Additional file 8. Supplementary Material 1: The materials and methods used in the present study. [file 13104_2024_6876_MOESM8_ESM.docx]

**Materials and Methods**

**Prediction of miR-497/195 cluster target genes and expression analysis**

miR-497/195 cluster targets gene expression in TCGA-CESC data set were evaluated using the Transcriptome Alterations in Cancer Omnibus (TACCO: <http://tacco.life.nctu.edu.tw/>) tool. TACCO is an online tool that provides users with an interface to assess the transcriptome, pathways, and clinical outcomes in cancers based on differential gene expression in TCGA datasets [26]. miRTarBase was used to identify the miR-497/195 cluster and its experimentally validated gene target interactions [27]. Analysis of the TCGA-CESC dataset identified 2020 genes (DEGs) that were differentially expressed between normal and tumor samples (±2-fold, p<0.05). We then computed the overlaps between the cluster gene targets and the DEGs in the TCGA-CESC cohort to identify the differentially expressed target genes (DETGs).

**Network construction of miR-497/195 cluster and its targets**

The miR-497/195 cluster target network with other RNA species, such as long noncoding RNAs (lncRNAs), circular RNAs (circRNAs), and small nuclear RNAs (sncRNAs), was constructed using miRNet (<https://www.mirnet.ca/>) with default parameters [28]. The coexpressed members of the cluster and their miRNA‒miRNA interactions were predicted using coexpression meta-analysis of miRNA targets (CoMeTa: <https://cometa.tigem.it/>) [29]. The conservation of the miRNA cluster across multiple species was predicted using an ECR (https://ecrbrowser.dcode.org/) [25]. The association between miR-497/195 cluster expression and clinicopathological conditions was evaluated using MEXPRESS (https://mexpress.be/) [26].

**Pathway and functional enrichment analysis**

Gene set enrichment analysis (GSEA) of DETGs was performed for biological process (BP), cellular component (Cell Comp), and molecular function (MF) terms using ShinyGO v0.741 (<http://bioinformatics.sdstate.edu/go/>) [30]. Furthermore, pathway enrichment analysis was performed by comparing the DETGs against the Kyoto Encyclopedia of Genes and Genomes (KEGG: <https://www.genome.jp/kegg/>) database. All the analyses were performed using the default setting of the tools used. p<0.05 indicated statistical significance.

**Construction of the protein-protein interaction (PPIN) network**

The potential PPIN was constructed for miR-497/195 clusters target that is differentially expressed using the Search Tool for the Retrieval of Interacting Genes (STRING: (<https://string-db.org/>) (confidence value of 0.9 and minimum interaction of 2) [31]. Based on the maximum degree of connectivity, the top 10 HGs of the PPIN were identified and visualized using the cytoHubba V 0.1 plugin of Cytoscape 3.8.2 (<https://cytoscape.org/>) [32]. Furthermore, we analyzed the expression of the ten Hub Genes (HGs) at the protein level using the Human Protein Atlas (HPA: <https://www.proteinatlas.org/>) [33]

***Prognostic significance of the miR-497/195 cluster and its target genes***

The association of differentially expressed HGs with metastasis was computed using the Human Cancer Metastasis Database (HCMDB: <https://hcmdb.i-sanger.com/>) [34]. The members of the cluster and its DETGs were subjected to survival analysis by generating Kaplan‒Meier survival curves with the log-rank method using the UALCAN tool. The Gene Expression Profiling Interactive Analysis 2.0 (<http://gepia2.cancer-pku.cn/>) tool was used to identify the DETGs affecting overall survival (OS) and disease-free survival (DFS) [35]. The random forest approach was employed to construct a prognostic model for the target genes using TACCO [26]. The immune infiltrates associated with the HGs were analysed using Tumor Immune Estimation Resource 2.0 (TIMER: <http://timer.cistrome.org/>) [36]. Critical immune prognostic predictors, such as CD4+ T cell, CD8+ T cell, and neutrophil populations, in CC, were examined [37]. The neutrophil-to-lymphocyte ratio is an independent prognosticator for survival outcomes in CC patients [38]. Therefore, the correlations of CD4+ T cells, CD8+ T cells, and neutrophils with metastatic gene expression were analysed using TIMER 2.0.

***Drug-target gene interactions***

Drug-gene interaction analysis was performed using the Drug-Gene Interaction Database 3.0 (DGIdb: <https://www.dgidb.org/>) to predict potential drugs targeting the cluster gene network [39]. Furthermore, PanDrugs (<https://www.pandrugs.org/>) analysis was used to prioritize the candidate drugs [40], STITCH 5 (<http://stitch.embl.de/>) was to visualize the drug-gene interaction network [41].

***Statistical analysis***

All statistical analysis was performed using GraphPad Prism v9.5. For differential expression analyses, Mann-Whitney’s test was employed and comparisons were significant if *p ≤ 0.05, **p ≤ 0.01, ***p*≤ 0.001, and ****p≤ 0.0001 respectively.

**References**

26. Chou PH, Liao WC, Tsai KW, Chen KC, Yu JS, Chen TW: **TACCO, a Database Connecting Transcriptome Alterations, Pathway Alterations and Clinical Outcomes in Cancers**. *Sci Rep* 2019, **9**(1):3877.

27. Huang HY, Lin YC, Cui S, Huang Y, Tang Y, Xu J, Bao J, Li Y, Wen J, Zuo H *et al*: **miRTarBase update 2022: an informative resource for experimentally validated miRNA-target interactions**. *Nucleic Acids Res* 2022, **50**(D1):D222-D230.

28. Chang L, Zhou G, Soufan O, Xia J: **miRNet 2.0: network-based visual analytics for miRNA functional analysis and systems biology**. *Nucleic Acids Res* 2020, **48**(W1):W244-W251.

29. Gennarino VA, D'Angelo G, Dharmalingam G, Fernandez S, Russolillo G, Sanges R, Mutarelli M, Belcastro V, Ballabio A, Verde P *et al*: **Identification of microRNA-regulated gene networks by expression analysis of target genes**. *Genome Res* 2012, **22**(6):1163-1172.

30. Ge SX, Jung D, Yao R: **ShinyGO: a graphical gene-set enrichment tool for animals and plants**. *Bioinformatics* 2020, **36**(8):2628-2629.

31. Szklarczyk D, Gable AL, Lyon D, Junge A, Wyder S, Huerta-Cepas J, Simonovic M, Doncheva NT, Morris JH, Bork P *et al*: **STRING v11: protein-protein association networks with increased coverage, supporting functional discovery in genome-wide experimental datasets**. *Nucleic Acids Res* 2019, **47**(D1):D607-D613.

32. Chin CH, Chen SH, Wu HH, Ho CW, Ko MT, Lin CY: **cytoHubba: identifying hub objects and sub-networks from complex interactome**. *BMC Syst Biol* 2014, **8 Suppl 4**(Suppl 4):S11.

33. Thul PJ, Lindskog C: **The human protein atlas: A spatial map of the human proteome**. *Protein Sci* 2018, **27**(1):233-244.

34. Zheng G, Ma Y, Zou Y, Yin A, Li W, Dong D: **HCMDB: the human cancer metastasis database**. *Nucleic Acids Res* 2018, **46**(D1):D950-D955.

35. Tang Z, Kang B, Li C, Chen T, Zhang Z: **GEPIA2: an enhanced web server for large-scale expression profiling and interactive analysis**. *Nucleic Acids Res* 2019, **47**(W1):W556-W560.

36. Li T, Fu J, Zeng Z, Cohen D, Li J, Chen Q, Li B, Liu XS: **TIMER2.0 for analysis of tumor-infiltrating immune cells**. *Nucleic Acids Res* 2020, **48**(W1):W509-W514.

37. Litwin TR, Irvin SR, Chornock RL, Sahasrabuddhe VV, Stanley M, Wentzensen N: **Infiltrating T-cell markers in cervical carcinogenesis: a systematic review and meta-analysis**. *Br J Cancer* 2021, **124**(4):831-841.

38. Zou P, Yang E, Li Z: **Neutrophil-to-lymphocyte ratio is an independent predictor for survival outcomes in cervical cancer: a systematic review and meta-analysis**. *Sci Rep* 2020, **10**(1):21917.

39. Cotto KC, Wagner AH, Feng YY, Kiwala S, Coffman AC, Spies G, Wollam A, Spies NC, Griffith OL, Griffith M: **DGIdb 3.0: a redesign and expansion of the drug-gene interaction database**. *Nucleic Acids Res* 2018, **46**(D1):D1068-D1073.

40. Pineiro-Yanez E, Reboiro-Jato M, Gomez-Lopez G, Perales-Paton J, Troule K, Rodriguez JM, Tejero H, Shimamura T, Lopez-Casas PP, Carretero J *et al*: **PanDrugs: a novel method to prioritize anticancer drug treatments according to individual genomic data**. *Genome Med* 2018, **10**(1):41.

41. Szklarczyk D, Santos A, von Mering C, Jensen LJ, Bork P, Kuhn M: **STITCH 5: augmenting protein-chemical interaction networks with tissue and affinity data**. *Nucleic Acids Res* 2016, **44**(D1):D380-384.
