# Supplementary material for: Analysis of miR-497/195 cluster identifies new therapeutic targets in cervical cancer
Source: BMC Res Notes. 2024 Aug 2;17:217. doi: 10.1186/s13104-024-06876-8 (PMC11297691; doi:10.1186/s13104-024-06876-8)
Supplement: Supplementary file 10 — Additional file 10: Table 1. List of differentially expressed miRNA cluster and its members in CC from small RNA sequencing data. [file 13104_2024_6876_MOESM10_ESM.docx]

Supplementary Table 1: The list of differentially expressed miRNA cluster and its members in CC from small RNA sequencing data.

| **S.No** | **miRNA Clusters** | **Mature.ID** | **Expression in NGS** |
| --- | --- | --- | --- |
| 1 | miR-1/133a cluster | hsa-miR-1 | -3.689126575 |
| 2 |  | hsa-miR-133a | -3.239382214 |
| 3 | miR-100/7a cluster | hsa-miR-100 | -3.030421962 |
| 4 | miR-122/3591 cluster | hsa-miR-3591 | -2.61646554 |
| 5 | miR-137/2682 Cluster | hsa-miR-137 | -5.260708324 |
| 6 | miR-143/145 cluster | hsa-miR-143 | -2.892557312 |
| 7 |  | hsa-miR-145 | -2.00372615 |
| 8 | miR-1912/1264 cluster | hsa-miR-1264 | -2.453302114 |
| 9 |  | hsa-miR-1912 | -1.956530765 |
| 10 | miR-193b/365a cluster | hsa-miR-365a | -1.516852719 |
| 11 | miR-196 cluster | hsa-miR-196a | -0.956578957 |
| 12 | miR-199a/214 cluster | hsa-miR-199a | -2.719920556 |
| 13 |  | hsa-miR-214 | -3.0296726 |
| 14 | miR-206/133b cluster | hsa-miR-133b | -2.000553317 |
| 15 | miR-212/132 cluster | hsa-miR-212 | -1.648448345 |
| 16 |  | hsa-miR-132 | -1.343608931 |
| 17 | miR-298/296 cluster | hsa-miR-296 | -1.712724698 |
| 18 | miR-29a cluster | hsa-miR-29a | -1.664212669 |
| 19 | miR-342/151b cluster | hsa-miR-342 | -1.392115346 |
| 20 | miR-3618/1306 cluster | hsa-miR-1306 | -1.961048955 |
| 21 |  | hsa-miR-655 | -3.526941133 |
| 22 |  | hsa-miR-381 | -3.512721364 |
| 23 |  | hsa-miR-487b | -3.34895716 |
| 24 |  | hsa-miR-154 | -3.192471525 |
| 25 |  | hsa-miR-299 | -3.131033568 |
| 26 |  | hsa-miR-654 | -3.037223129 |
| 27 |  | hsa-miR-376a | -2.962153111 |
| 28 |  | hsa-miR-376b | -2.922663708 |
| 29 |  | hsa-miR-329 | -2.846835033 |
| 30 |  | hsa-miR-1185-1 | -2.843235883 |
| 31 |  | hsa-miR-376c | -2.825429917 |
| 32 |  | hsa-miR-495 | -2.817754993 |
| 33 |  | hsa-miR-656 | -2.694527214 |
| 34 |  | hsa-miR-134 | -2.692604775 |
| 35 | miR-379/656 cluster | hsa-miR-539 | -2.649274472 |
| 36 |  | hsa-miR-485 | -2.575265389 |
| 37 |  | hsa-miR-1185-2 | -2.514019055 |
| 38 |  | hsa-miR-379 | -2.509921273 |
| 39 |  | hsa-miR-758 | -2.46877805 |
| 40 |  | hsa-miR-409 | -2.383408664 |
| 41 |  | hsa-miR-487a | -2.3013612 |
| 42 |  | hsa-miR-376a-2 | -2.166274223 |
| 43 |  | hsa-miR-494 | -2.14532608 |
| 44 |  | hsa-miR-1185-1 | -2.051874898 |
| 45 |  | hsa-miR-380 | -2.027889514 |
| 46 |  | hsa-miR-377 | -1.905221755 |
| 47 |  | hsa-miR-323a | -1.878891262 |
| 48 |  | hsa-miR-543 | -1.824423002 |
| 49 |  | hsa-miR-382 | -1.818950835 |
| 50 |  | hsa-miR-411 | -1.736256464 |
| 51 |  | hsa-miR-369 | -1.630079406 |
| 52 | miR-3910 cluster | hsa-miR-3910 | -1.960703294 |
| 53 | miR-3960/2861 cluster | hsa-miR-3960 | -2.361139263 |
| 54 | miR-423/3184 cluster | hsa-miR-423 | -1.656084748 |
| 55 | miR-424/450b cluster | hsa-miR-450a-2 | -3.278706353 |
| 56 |  | hsa-miR-450b | -2.929365412 |
| 57 | miR-424/450b cluster | hsa-miR-424 | -2.598398537 |
| 58 |  | hsa-miR-450a | -2.362874149 |
| 59 |  | hsa-miR-542 | -1.631271823 |
| 60 |  | hsa-miR-503 | -1.404072598 |
| 61 | miR-4725/365b cluster | hsa-miR-365b | -1.516852719 |
| 62 | miR-489/653 cluster | hsa-miR-489 | -2.047553741 |
| 63 | miR-493/136 cluster | hsa-miR-433 | -3.991388891 |
| 64 |  | hsa-miR-337 | -3.632090346 |
| 65 |  | hsa-miR-432 | -3.494837875 |
| 66 |  | hsa-miR-136 | -3.322606206 |
| 67 |  | hsa-miR-665 | -2.468541222 |
| 68 |  | hsa-miR-127 | -2.428186539 |
| 69 |  | hsa-miR-493 | -1.653840687 |
| 70 | miR-497/ 195 cluster | hsa-miR-497 | -2.548056656 |
| 71 |  | hsa-miR-195 | -1.876138204 |
| 72 | miR-99b/125 cluster | hsa-miR-125a | -2.147805949 |
| 73 |  | hsa-let-7e | -1.897986717 |
| 74 |  | hsa-miR-99b | -1.74890862 |
| 75 | miR-106/363 cluster | hsa-miR-20b | 3.856484756 |
| 76 |  | hsa-miR-19b | 1.494672422 |
| 77 |  | hsa-miR-363 | 3.922960789 |
| 78 |  | hsa-miR-18b | 3.578053011 |
| 79 |  | hsa-miR-106a | 3.313067527 |
| 80 | miR-106b/25 Cluster | hsa-miR-106b | 1.948333524 |
| 81 |  | hsa-miR-25 | 1.57114627 |
| 82 |  | hsa-miR-93 | 1.187553109 |
| 83 | miR-1179/3529 cluster | hsa-miR-7 | 1.924654855 |
| 84 | miR-1250/338 cluster | hsa-miR-338 | 1.794769063 |
| 85 | miR-15a/16-1 cluster | hsa-miR-15a | 2.974560862 |
| 86 |  | hsa-miR-16-2 | 3.388717661 |
| 87 | miR-15b/16-2 cluster | hsa-miR-15b | 2.74657455 |
| 88 | miR-17/92 cluster | hsa-miR-18a | 2.372920556 |
| 89 |  | hsa-miR-20a | 2.185993915 |
| 90 |  | hsa-miR-92a | 1.701566011 |
| 91 |  | hsa-miR-19a | 1.529610582 |
| 92 |  | hsa-miR-19b-1 | 1.494672422 |
| 93 |  | hsa-miR-17 | 1.040909278 |
| 94 | miR-181 cluster | hsa-miR-181b | 1.722827952 |
| 95 |  | hsa-miR-181a-2 | 1.624846656 |
| 96 | miR-182/183 cluster | hsa-miR-96 | 2.896796113 |
| 97 |  | hsa-miR-183 | 2.489411009 |
| 98 |  | hsa-miR-182 | 2.031746189 |
| 99 | miR-191/425 cluster | hsa-miR-425 | 2.138260498 |
| 100 | miR-200a/429 cluster | hsa-miR-200a | 1.81389971 |
| 101 |  | hsa-miR-429 | 1.689364246 |
| 102 |  | hsa-miR-200b | 1.376302614 |
| 103 | miR-200c/141 cluster | hsa-miR-141 | 1.704453624 |
| 104 |  | hsa-miR-200c | 1.276244186 |
| 105 | miR-221/222 cluster | hsa-miR-222 | 1.334785224 |
| 106 |  | hsa-miR-221 | 1.18298287 |
| 107 | miR-301b/130b cluster | hsa-miR-130b | 2.035848397 |
| 108 | miR-33b/6777 cluster | hsa-miR-33b | 2.080321632 |
| 109 | miR-34 cluster | hsa-miR-34b | 2.697948794 |
| 110 |  | hsa-miR-34c | 2.849117111 |
| 111 | miR-3913 cluster | hsa-miR-3913 | 1.682126286 |
| 112 | miR-449 cluster | hsa-miR-449a | 1.775956174 |
| 113 | miR-452/224 cluster | hsa-miR-224 | 2.530517374 |
| 114 | miR-4732/451a cluster | hsa-miR-144 | 4.358115718 |
| 115 |  | hsa-miR-451a | 4.05567325 |
| 116 | miR-4736/142 cluster | hsa-miR-142 | 2.638348196 |
| 117 | miR-486 cluster | hsa-miR-486 | 5.193874562 |
