# Supplementary material for: Analysis of miR-497/195 cluster identifies new therapeutic targets in cervical cancer
Source: BMC Res Notes. 2024 Aug 2;17:217. doi: 10.1186/s13104-024-06876-8 (PMC11297691; doi:10.1186/s13104-024-06876-8)
Supplement: Supplementary file 11 — Additional file 11: Table 2. List of differentially expressed genes in CC from the TCGA-CESC dataset. [file 13104_2024_6876_MOESM11_ESM.docx]

**Supplementary Table 2: List of differentially expressed genes in CC from TCGA-CESC dataset.**

| **Gene** | **Fold change** | **log2 (Fold change)** | **Mean TPM* (tumor)** | **Mean TPM* (normal)** | **p-value** | **adjusted p-value** |
| --- | --- | --- | --- | --- | --- | --- |
| CNN1 | -191.82 | -7.58 | 310.51 | 59562.65 | 0.00291 | 0.0418 |
| DES | -191.27 | -7.58 | 500.12 | 95655.66 | 0.00291 | 0.0418 |
| PTGER3 | -170.68 | -7.42 | 29.31 | 5002.59 | 0.00323 | 0.0418 |
| ACTG2 | -168.17 | -7.39 | 464.45 | 78108.93 | 0.00297 | 0.0418 |
| DPP6 | -160.82 | -7.33 | 15.13 | 2434.09 | 0.00295 | 0.0418 |
| PI16 | -148.14 | -7.21 | 35.66 | 5283.2 | 0.00648 | 0.043 |
| GPM6A | -143.52 | -7.17 | 5.3 | 761.2 | 0.00272 | 0.0418 |
| CHRDL1 | -142.76 | -7.16 | 34.92 | 4985.04 | 0.00292 | 0.0418 |
| PGM5 | -131.88 | -7.04 | 75.54 | 9962.04 | 0.00291 | 0.0418 |
| OGN | -129.51 | -7.02 | 42.09 | 5451.9 | 0.00303 | 0.0418 |
| MYH11 | -122.14 | -6.93 | 1125.38 | 137458.64 | 0.00291 | 0.0418 |
| ANGPTL1 | -118.57 | -6.89 | 15.12 | 1793.38 | 0.00303 | 0.0418 |
| SGCA | -115.65 | -6.85 | 5.98 | 691.54 | 0.00297 | 0.0418 |
| CRHBP | -114.48 | -6.84 | 2.12 | 242.47 | 0.003 | 0.0418 |
| NBLA00301 | -111.68 | -6.8 | 12.55 | 1401.58 | 0.00297 | 0.0418 |
| HRNBP3 | -109.62 | -6.78 | 3.31 | 362.72 | 0.00303 | 0.0418 |
| PLN | -108.15 | -6.76 | 29.64 | 3205.34 | 0.00291 | 0.0418 |
| CHRDL2 | -107.36 | -6.75 | 35.13 | 3771.61 | 0.00309 | 0.0418 |
| ZCCHC12 | -107.2 | -6.74 | 15.75 | 1688.7 | 0.00297 | 0.0418 |
| C7 | -98.61 | -6.62 | 64.5 | 6360.49 | 0.00296 | 0.0418 |
| CASQ2 | -93.43 | -6.55 | 5.41 | 505.8 | 0.00287 | 0.0418 |
| ADH1B | -93.19 | -6.54 | 38.13 | 3553.24 | 0.00294 | 0.0418 |
| PTGIS | -93.23 | -6.54 | 87.35 | 8143.88 | 0.00304 | 0.0418 |
| CASP12 | -91.12 | -6.51 | 0.58 | 52.52 | 0.00197 | 0.0418 |
| CCL14 | -91.15 | -6.51 | 58.07 | 5293.05 | 0.00291 | 0.0418 |
| GPIHBP1 | -89.31 | -6.48 | 5.03 | 449.14 | 0.0029 | 0.0418 |
| FXYD1 | -89.43 | -6.48 | 23.45 | 2097.07 | 0.0029 | 0.0418 |
| GSTM5 | -89.48 | -6.48 | 20.91 | 1871.07 | 0.00291 | 0.0418 |
| LMOD1 | -88.2 | -6.46 | 206.69 | 18230.18 | 0.00291 | 0.0418 |
| TRPC4 | -88.16 | -6.46 | 9.41 | 830.1 | 0.00297 | 0.0418 |
| PEG3 | -85.79 | -6.42 | 21.43 | 1838.92 | 0.0031 | 0.0418 |
| NCAM1 | -84.22 | -6.4 | 58.24 | 4905.16 | 0.00291 | 0.0418 |
| HSPB7 | -83.19 | -6.38 | 32.3 | 2686.81 | 0.00291 | 0.0418 |
| JPH4 | -82.42 | -6.36 | 14.73 | 1214.34 | 0.0031 | 0.0418 |
| LEFTY2 | -82.38 | -6.36 | 33.05 | 2722.86 | 0.00317 | 0.0418 |
| HAND2 | -79.38 | -6.31 | 33.82 | 2684.78 | 0.00304 | 0.0418 |
| CSDC2 | -74.86 | -6.23 | 31.33 | 2345.65 | 0.00291 | 0.0418 |
| HSPB6 | -71.6 | -6.16 | 204.76 | 14660.89 | 0.00291 | 0.0418 |
| DPT | -71.05 | -6.15 | 91.53 | 6503.49 | 0.00291 | 0.0418 |
| MYOCD | -70.6 | -6.14 | 9.25 | 653.41 | 0.00301 | 0.0418 |
| PRND | -69.74 | -6.12 | 2.6 | 181.51 | 0.00269 | 0.0418 |
| TCEAL2 | -69.4 | -6.12 | 18.52 | 1285.47 | 0.00303 | 0.0418 |
| OPCML | -69.07 | -6.11 | 1.47 | 101.54 | 0.00257 | 0.0418 |
| VEGFD | -67.51 | -6.08 | 5.59 | 377.6 | 0.0029 | 0.0418 |
| ABCA8 | -66.98 | -6.07 | 15.19 | 1017.18 | 0.00289 | 0.0418 |
| BPI | -66.54 | -6.06 | 1.48 | 98.79 | 0.00375 | 0.0418 |
| DARC | -65.13 | -6.03 | 183.82 | 11972.46 | 0.0031 | 0.0418 |
| SCN7A | -64.72 | -6.02 | 2.19 | 141.5 | 0.00224 | 0.0418 |
| MASP1 | -64.23 | -6.01 | 95.56 | 6137.27 | 0.00291 | 0.0418 |
| FAM180B | -64.4 | -6.01 | 0.52 | 33.57 | 0.00325 | 0.0418 |
| RBM24 | -64.08 | -6 | 13.75 | 881.52 | 0.00359 | 0.0418 |
| ACTA2 | -62.63 | -5.97 | 2693.67 | 168693 | 0.00291 | 0.0418 |
| KCNB1 | -59.59 | -5.9 | 1.28 | 76.51 | 0.00179 | 0.0418 |
| RSPO1 | -57.94 | -5.86 | 15.25 | 883.9 | 0.00296 | 0.0418 |
| KLHL4 | -58.21 | -5.86 | 4.51 | 262.84 | 0.0032 | 0.0418 |
| PLAC9 | -57.79 | -5.85 | 16.82 | 972.33 | 0.0031 | 0.0418 |
| PGM5P2 | -55.32 | -5.79 | 1.52 | 84.11 | 0.00337 | 0.0418 |
| ADAMTS5 | -54.69 | -5.77 | 52.68 | 2880.96 | 0.00323 | 0.0418 |
| LINGO2 | -54.01 | -5.76 | 5.69 | 307.54 | 0.00754 | 0.0445 |
| SYT9 | -53.54 | -5.74 | 1.41 | 75.55 | 0.00271 | 0.0418 |
| ZBTB16 | -52.93 | -5.73 | 5.75 | 304.62 | 0.003 | 0.0418 |
| DGKB | -52.3 | -5.71 | 3.82 | 199.83 | 0.00266 | 0.0418 |
| LONRF2 | -51.51 | -5.69 | 60.46 | 3114.33 | 0.00316 | 0.0418 |
| KCNMB1 | -50.92 | -5.67 | 81.06 | 4127.28 | 0.00291 | 0.0418 |
| PSD | -50.53 | -5.66 | 61.46 | 3105.59 | 0.00304 | 0.0418 |
| WT1 | -50.73 | -5.66 | 53.65 | 2722.08 | 0.00323 | 0.0418 |
| BNC2 | -49.46 | -5.63 | 78.51 | 3883.45 | 0.00304 | 0.0418 |
| CD300LG | -47.82 | -5.58 | 3.09 | 147.82 | 0.00272 | 0.0418 |
| CLEC3B | -46.34 | -5.53 | 60.07 | 2783.81 | 0.00291 | 0.0418 |
| PRUNE2 | -45.83 | -5.52 | 81.61 | 3740.78 | 0.00304 | 0.0418 |
| TCF21 | -45.18 | -5.5 | 12.12 | 547.44 | 0.00289 | 0.0418 |
| MAMDC2 | -45.25 | -5.5 | 50.93 | 2304.62 | 0.00303 | 0.0418 |
| TAGLN | -45.08 | -5.49 | 4248.07 | 191501.22 | 0.00291 | 0.0418 |
| KCNMA1 | -43.94 | -5.46 | 165.36 | 7266.34 | 0.00291 | 0.0418 |
| ANGPTL7 | -43.19 | -5.43 | 1.39 | 59.9 | 0.00464 | 0.0418 |
| GPR133 | -42.9 | -5.42 | 15.71 | 674.12 | 0.00304 | 0.0418 |
| PCP4 | -42.39 | -5.41 | 50.24 | 2129.79 | 0.00359 | 0.0418 |
| TNXB | -42.17 | -5.4 | 272.09 | 11474 | 0.00291 | 0.0418 |
| MRGPRF | -42 | -5.39 | 139.16 | 5845.29 | 0.00291 | 0.0418 |
| PLP1 | -42.01 | -5.39 | 7.19 | 302.03 | 0.00327 | 0.0418 |
| FHL1 | -41.7 | -5.38 | 377.38 | 15738.46 | 0.00291 | 0.0418 |
| CACNB2 | -40.72 | -5.35 | 12.04 | 490.34 | 0.00291 | 0.0418 |
| TCEAL7 | -40.17 | -5.33 | 17.86 | 717.43 | 0.00291 | 0.0418 |
| C2orf40 | -40.15 | -5.33 | 9.96 | 399.92 | 0.00305 | 0.0418 |
| RSPO3 | -40.16 | -5.33 | 62.44 | 2507.39 | 0.00575 | 0.0421 |
| C11orf96 | -39.9 | -5.32 | 262.63 | 10479.06 | 0.00291 | 0.0418 |
| AOC3 | -39.96 | -5.32 | 132.55 | 5296.28 | 0.00304 | 0.0418 |
| SORBS1 | -39.63 | -5.31 | 221.91 | 8793.32 | 0.00304 | 0.0418 |
| LRRN4CL | -39.51 | -5.3 | 38.84 | 1534.4 | 0.00291 | 0.0418 |
| SPARCL1 | -38.72 | -5.28 | 1703.17 | 65951.29 | 0.00291 | 0.0418 |
| SMOC2 | -38.62 | -5.27 | 266.77 | 10303.54 | 0.00291 | 0.0418 |
| PGR | -38.44 | -5.26 | 209.65 | 8059.15 | 0.0033 | 0.0418 |
| ATRNL1 | -38.41 | -5.26 | 16.94 | 650.62 | 0.005 | 0.0418 |
| FHL5 | -37.67 | -5.24 | 6.05 | 227.95 | 0.00303 | 0.0418 |
| LIMS2 | -37.62 | -5.23 | 205 | 7712.62 | 0.00291 | 0.0418 |
| RERGL | -37.64 | -5.23 | 8.3 | 312.55 | 0.00313 | 0.0418 |
| TRH | -37.53 | -5.23 | 7.1 | 266.63 | 0.00413 | 0.0418 |
| DACT3 | -37.28 | -5.22 | 62.35 | 2324.11 | 0.00291 | 0.0418 |
| PLCL1 | -37.32 | -5.22 | 35.33 | 1318.64 | 0.00375 | 0.0418 |
| TRIM63 | -36.79 | -5.2 | 1.57 | 57.64 | 0.0024 | 0.0418 |
| MFAP5 | -36.7 | -5.2 | 346.39 | 12712.35 | 0.0082 | 0.0456 |
| LOC145820 | -36.06 | -5.17 | 1.55 | 55.95 | 0.00274 | 0.0418 |
| ADGRB3 | -35.85 | -5.16 | 1.76 | 63.26 | 0.00211 | 0.0418 |
| CTSG | -35.73 | -5.16 | 9.46 | 337.94 | 0.00289 | 0.0418 |
| CACNA1C | -35.56 | -5.15 | 74.57 | 2651.24 | 0.00304 | 0.0418 |
| BCHE | -34.92 | -5.13 | 19.29 | 673.59 | 0.00315 | 0.0418 |
| CXCL12 | -34.77 | -5.12 | 304.74 | 10594.83 | 0.00291 | 0.0418 |
| KLHL38 | -34.69 | -5.12 | 1.21 | 42.02 | 0.00601 | 0.0423 |
| LOC728264 | -34.63 | -5.11 | 80.83 | 2799.6 | 0.00291 | 0.0418 |
| RPRM | -34.45 | -5.11 | 27.09 | 933.29 | 0.00291 | 0.0418 |
| C1QTNF7 | -34.19 | -5.1 | 16.76 | 573.19 | 0.00291 | 0.0418 |
| F10 | -34.21 | -5.1 | 25.87 | 885.2 | 0.00304 | 0.0418 |
| MYLK | -33.31 | -5.06 | 983.48 | 32763.28 | 0.00304 | 0.0418 |
| RERG | -33.07 | -5.05 | 122.77 | 4059.65 | 0.00291 | 0.0418 |
| OMD | -33.24 | -5.05 | 12.17 | 404.54 | 0.00302 | 0.0418 |
| HPSE2 | -33.13 | -5.05 | 3.75 | 124.13 | 0.00325 | 0.0418 |
| EMCN | -32.72 | -5.03 | 66.91 | 2189.38 | 0.00291 | 0.0418 |
| CCDC85A | -32.62 | -5.03 | 17.43 | 568.71 | 0.0049 | 0.0418 |
| ABCC9 | -32.41 | -5.02 | 36.04 | 1167.98 | 0.00297 | 0.0418 |
| MEOX2 | -32.43 | -5.02 | 5.42 | 175.65 | 0.00331 | 0.0418 |
| LYVE1 | -32.03 | -5 | 18.56 | 594.34 | 0.00383 | 0.0418 |
| PYGM | -31.5 | -4.98 | 10.88 | 342.77 | 0.00304 | 0.0418 |
| TNFAIP8L3 | -31.28 | -4.97 | 101.75 | 3183.37 | 0.00304 | 0.0418 |
| CACNA1H | -31.45 | -4.97 | 126.75 | 3985.9 | 0.0031 | 0.0418 |
| ABCG2 | -31.17 | -4.96 | 34.14 | 1064.25 | 0.00291 | 0.0418 |
| LCN10 | -31.13 | -4.96 | 9.51 | 296.23 | 0.00323 | 0.0418 |
| PTGFR | -31.17 | -4.96 | 24.37 | 759.64 | 0.00323 | 0.0418 |
| HFM1 | -30.87 | -4.95 | 1.77 | 54.76 | 0.00243 | 0.0418 |
| GREM2 | -30.65 | -4.94 | 18.76 | 574.99 | 0.00359 | 0.0418 |
| JAM2 | -30.45 | -4.93 | 31.44 | 957.32 | 0.00291 | 0.0418 |
| SCRG1 | -30.22 | -4.92 | 3.22 | 97.37 | 0.0024 | 0.0418 |
| RGS22 | -30.36 | -4.92 | 3.45 | 104.62 | 0.00271 | 0.0418 |
| NOVA1 | -29.61 | -4.89 | 11.97 | 354.54 | 0.00303 | 0.0418 |
| CYTL1 | -29.45 | -4.88 | 15.02 | 442.37 | 0.00352 | 0.0418 |
| MYL9 | -29.17 | -4.87 | 2641.86 | 77070.17 | 0.00291 | 0.0418 |
| PI15 | -29.2 | -4.87 | 58.46 | 1707.36 | 0.00399 | 0.0418 |
| SYNPO2 | -28.78 | -4.85 | 410.38 | 11810.74 | 0.0033 | 0.0418 |
| MYOZ2 | -28.64 | -4.84 | 0.82 | 23.53 | 0.0019 | 0.0418 |
| MMRN1 | -28.54 | -4.83 | 55.68 | 1589.08 | 0.00291 | 0.0418 |
| CCDC181 | -28.37 | -4.83 | 3.63 | 102.88 | 0.00303 | 0.0418 |
| HTR2B | -28.44 | -4.83 | 40.18 | 1142.66 | 0.00304 | 0.0418 |
| GPR22 | -27.97 | -4.81 | 1.47 | 41.01 | 0.00474 | 0.0418 |
| NAP1L2 | -27.91 | -4.8 | 9.23 | 257.65 | 0.00323 | 0.0418 |
| JPH2 | -27.7 | -4.79 | 90.67 | 2511.95 | 0.00291 | 0.0418 |
| LDB2 | -27.66 | -4.79 | 107.56 | 2974.84 | 0.00291 | 0.0418 |
| PDZRN3 | -27.44 | -4.78 | 116.32 | 3191.95 | 0.00291 | 0.0418 |
| PDLIM3 | -27.47 | -4.78 | 167.04 | 4589.1 | 0.00323 | 0.0418 |
| HSPB2 | -26.88 | -4.75 | 24.12 | 648.43 | 0.00291 | 0.0418 |
| PDE2A | -26.94 | -4.75 | 63.95 | 1722.8 | 0.00291 | 0.0418 |
| STAB2 | -26.87 | -4.75 | 1.79 | 48.1 | 0.0039 | 0.0418 |
| FBXL22 | -26.64 | -4.74 | 16.22 | 432.03 | 0.00304 | 0.0418 |
| NXPH3 | -26.81 | -4.74 | 57.83 | 1550.55 | 0.00304 | 0.0418 |
| WIT1 | -26.72 | -4.74 | 12.24 | 327 | 0.0032 | 0.0418 |
| C1QTNF9 | -26.47 | -4.73 | 1.26 | 33.32 | 0.00239 | 0.0418 |
| SCN2B | -26.46 | -4.73 | 4.44 | 117.48 | 0.00327 | 0.0418 |
| ABCA9 | -26.34 | -4.72 | 16.31 | 429.56 | 0.00291 | 0.0418 |
| GDAP1L1 | -25.88 | -4.69 | 0.89 | 22.99 | 0.00247 | 0.0418 |
| DMRTC1B | -25.9 | -4.69 | 1.48 | 38.37 | 0.00248 | 0.0418 |
| CDO1 | -25.86 | -4.69 | 12.06 | 311.81 | 0.00329 | 0.0418 |
| RAI2 | -25.69 | -4.68 | 85.71 | 2201.51 | 0.00291 | 0.0418 |
| SNTG2 | -25.27 | -4.66 | 3.16 | 79.91 | 0.00262 | 0.0418 |
| SLC5A4 | -25.1 | -4.65 | 0.78 | 19.66 | 0.0025 | 0.0418 |
| DCN | -25.12 | -4.65 | 2508.82 | 63030.25 | 0.00291 | 0.0418 |
| FGF7 | -25.14 | -4.65 | 73.22 | 1840.59 | 0.00291 | 0.0418 |
| ATOH8 | -24.93 | -4.64 | 56.44 | 1407.17 | 0.00291 | 0.0418 |
| RASL12 | -24.97 | -4.64 | 91.54 | 2285.89 | 0.00291 | 0.0418 |
| AQP8 | -24.9 | -4.64 | 1.63 | 40.65 | 0.00566 | 0.042 |
| SLC7A3 | -24.85 | -4.63 | 6.32 | 157.04 | 0.00285 | 0.0418 |
| MYL3 | -24.65 | -4.62 | 1.19 | 29.41 | 0.00258 | 0.0418 |
| ATP1B2 | -24.55 | -4.62 | 22.17 | 544.25 | 0.0033 | 0.0418 |
| KIAA1644 | -24.46 | -4.61 | 91.14 | 2229.18 | 0.00297 | 0.0418 |
| NDP | -24.46 | -4.61 | 16.55 | 404.81 | 0.00604 | 0.0424 |
| FAM110D | -24.21 | -4.6 | 19.69 | 476.76 | 0.00291 | 0.0418 |
| **Gene** | **Fold change** | **log2 (Fold change)** | **Mean TPM* (tumor)** | **Mean TPM* (normal)** | **p-value** | **adjusted p-value** |
| TMEM132C | -24.22 | -4.6 | 15.91 | 385.4 | 0.00331 | 0.0418 |
| NECAB1 | -24.03 | -4.59 | 29.22 | 702.35 | 0.00291 | 0.0418 |
| RUNDC3B | -24.03 | -4.59 | 12.57 | 302.2 | 0.00304 | 0.0418 |
| KCTD8 | -23.91 | -4.58 | 1.88 | 44.95 | 0.00275 | 0.0418 |
| GPRASP1 | -23.93 | -4.58 | 67.18 | 1607.52 | 0.00291 | 0.0418 |
| RAMP1 | -23.97 | -4.58 | 403.71 | 9676.74 | 0.00297 | 0.0418 |
| MGARP | -23.89 | -4.58 | 5.81 | 138.79 | 0.00367 | 0.0418 |
| HRC | -23.69 | -4.57 | 6.39 | 151.39 | 0.0031 | 0.0418 |
| MSRB3 | -23.57 | -4.56 | 266.01 | 6269.47 | 0.00291 | 0.0418 |
| PODN | -23.58 | -4.56 | 207.6 | 4894.7 | 0.00291 | 0.0418 |
| TACR1 | -23.36 | -4.55 | 19.15 | 447.26 | 0.00297 | 0.0418 |
| FLNC | -23.39 | -4.55 | 423.31 | 9901.94 | 0.00323 | 0.0418 |
| ADAMTSL1 | -23.45 | -4.55 | 56.11 | 1315.85 | 0.00345 | 0.0418 |
| ITGB1BP2 | -23.28 | -4.54 | 10.82 | 251.8 | 0.00291 | 0.0418 |
| ARHGAP6 | -23.17 | -4.53 | 29.13 | 675.14 | 0.00291 | 0.0418 |
| CPED1 | -23.14 | -4.53 | 50.34 | 1164.72 | 0.00291 | 0.0418 |
| CAVIN2 | -23.14 | -4.53 | 151.16 | 3498.29 | 0.00323 | 0.0418 |
| ITIH3 | -23.04 | -4.53 | 7.25 | 167.13 | 0.00432 | 0.0418 |
| PABPC5 | -22.97 | -4.52 | 3.7 | 85.01 | 0.00297 | 0.0418 |
| INMT | -22.75 | -4.51 | 33.03 | 751.43 | 0.00317 | 0.0418 |
| PIANP | -22.8 | -4.51 | 25.89 | 590.24 | 0.00337 | 0.0418 |
| ADAM33 | -22.41 | -4.49 | 86.28 | 1933.64 | 0.00297 | 0.0418 |
| CXorf36 | -22.39 | -4.48 | 114.5 | 2563.39 | 0.00291 | 0.0418 |
| PPAPDC3 | -22.26 | -4.48 | 12.14 | 270.12 | 0.00291 | 0.0418 |
| NPAS4 | -22.26 | -4.48 | 2.11 | 47.05 | 0.0031 | 0.0418 |
| PTH1R | -22.38 | -4.48 | 15.38 | 344.24 | 0.0031 | 0.0418 |
| PDK4 | -22.34 | -4.48 | 189.85 | 4240.78 | 0.00317 | 0.0418 |
| COL14A1 | -22.26 | -4.48 | 360.03 | 8014.59 | 0.00323 | 0.0418 |
| ANGPT4 | -22.24 | -4.47 | 0.56 | 12.51 | 0.0016 | 0.0418 |
| NDN | -22.15 | -4.47 | 140.51 | 3112.38 | 0.00291 | 0.0418 |
| AGTR1 | -22.12 | -4.47 | 4.69 | 103.84 | 0.00338 | 0.0418 |
| FXYD6 | -21.92 | -4.45 | 378.87 | 8305.73 | 0.00297 | 0.0418 |
| KCNE4 | -21.63 | -4.43 | 44.82 | 969.45 | 0.00304 | 0.0418 |
| LGI4 | -21.36 | -4.42 | 48.17 | 1029.09 | 0.00291 | 0.0418 |
| NTRK3 | -21.37 | -4.42 | 8.79 | 187.98 | 0.00325 | 0.0418 |
| GFRA1 | -21.07 | -4.4 | 58.25 | 1227.39 | 0.00337 | 0.0418 |
| WSCD2 | -21.15 | -4.4 | 22.75 | 481.18 | 0.0035 | 0.0418 |
| SLC35F1 | -21.18 | -4.4 | 5.37 | 113.82 | 0.00359 | 0.0418 |
| PLIN4 | -20.93 | -4.39 | 80.83 | 1691.94 | 0.00323 | 0.0418 |
| FREM1 | -20.9 | -4.39 | 43.09 | 900.56 | 0.00407 | 0.0418 |
| GNG11 | -20.78 | -4.38 | 187.51 | 3896.43 | 0.00291 | 0.0418 |
| MEIS3 | -20.84 | -4.38 | 75.79 | 1579.55 | 0.00304 | 0.0418 |
| ASPA | -20.89 | -4.38 | 3.77 | 78.69 | 0.00313 | 0.0418 |
| NRXN3 | -20.85 | -4.38 | 29.94 | 624.18 | 0.00788 | 0.0452 |
| NAP1L3 | -20.69 | -4.37 | 17.96 | 371.56 | 0.00297 | 0.0418 |
| PCDHGA2 | -20.69 | -4.37 | 4.92 | 101.9 | 0.00478 | 0.0418 |
| CILP | -20.61 | -4.37 | 57.83 | 1192.05 | 0.00759 | 0.0445 |
| PRELP | -20.56 | -4.36 | 409.8 | 8427.44 | 0.00291 | 0.0418 |
| TCEAL6 | -20.51 | -4.36 | 22.03 | 451.94 | 0.00291 | 0.0418 |
| EMX2 | -20.49 | -4.36 | 136.74 | 2801.41 | 0.00391 | 0.0418 |
| ITGA11 | -20.42 | -4.35 | 238.8 | 4875.82 | 0.00304 | 0.0418 |
| MEOX1 | -20.45 | -4.35 | 38.8 | 793.57 | 0.00317 | 0.0418 |
| WNT9B | -20.3 | -4.34 | 1.27 | 25.81 | 0.00245 | 0.0418 |
| CNRIP1 | -20.29 | -4.34 | 61.68 | 1251.66 | 0.00291 | 0.0418 |
| TCEAL5 | -20.3 | -4.34 | 10.39 | 211.03 | 0.00297 | 0.0418 |
| CLIP3 | -20.32 | -4.34 | 156.27 | 3175.49 | 0.00304 | 0.0418 |
| ENPP2 | -20.28 | -4.34 | 250.55 | 5081.47 | 0.0031 | 0.0418 |
| EMX2OS | -20.06 | -4.33 | 148.51 | 2979.77 | 0.00352 | 0.0418 |
| ASPN | -20.02 | -4.32 | 182.78 | 3659.78 | 0.00291 | 0.0418 |
| MRVI1 | -19.98 | -4.32 | 179.65 | 3589.58 | 0.00291 | 0.0418 |
| MUSTN1 | -20.01 | -4.32 | 33.14 | 662.97 | 0.00291 | 0.0418 |
| ANK2 | -19.98 | -4.32 | 63.17 | 1262.19 | 0.00304 | 0.0418 |
| FAM180A | -19.86 | -4.31 | 6.75 | 134.14 | 0.00309 | 0.0418 |
| MFAP4 | -19.73 | -4.3 | 514.17 | 10142.88 | 0.0031 | 0.0418 |
| PURG | -19.65 | -4.3 | 2.95 | 58.02 | 0.0038 | 0.0418 |
| PLK5P | -19.64 | -4.3 | 3.26 | 64.05 | 0.00437 | 0.0418 |
| OLFML3 | -19.55 | -4.29 | 351.3 | 6869.64 | 0.00291 | 0.0418 |
| SELP | -19.59 | -4.29 | 50.16 | 982.57 | 0.00317 | 0.0418 |
| LOC134466 | -19.59 | -4.29 | 7.82 | 153.09 | 0.0033 | 0.0418 |
| GALNT17 | -19.54 | -4.29 | 15.33 | 299.6 | 0.00385 | 0.0418 |
| FBXL7 | -19.39 | -4.28 | 84.56 | 1639.61 | 0.00291 | 0.0418 |
| ENPP1 | -19.41 | -4.28 | 91.6 | 1777.67 | 0.0031 | 0.0418 |
| C20orf200 | -19.41 | -4.28 | 3.91 | 75.9 | 0.00321 | 0.0418 |
| CCDC89 | -19.4 | -4.28 | 5.19 | 100.68 | 0.00367 | 0.0418 |
| MAP6 | -19.32 | -4.27 | 24.44 | 472.04 | 0.00291 | 0.0418 |
| CA4 | -19.32 | -4.27 | 4.45 | 85.95 | 0.00304 | 0.0418 |
| LOC26102 | -19.18 | -4.26 | 0.55 | 10.59 | 0.00188 | 0.0418 |
| FBLN5 | -19.14 | -4.26 | 430.91 | 8247.25 | 0.00291 | 0.0418 |
| ENPEP | -19.2 | -4.26 | 61.39 | 1178.89 | 0.00836 | 0.046 |
| MGP | -18.88 | -4.24 | 2195.11 | 41451.58 | 0.00383 | 0.0418 |
| PPP1R14A | -18.81 | -4.23 | 50.51 | 949.9 | 0.00304 | 0.0418 |
| LRRC2 | -18.75 | -4.23 | 18.6 | 348.77 | 0.00359 | 0.0418 |
| SERPIND1 | -18.82 | -4.23 | 5.47 | 103.05 | 0.00719 | 0.0439 |
| C1orf70 | -18.67 | -4.22 | 6.42 | 119.89 | 0.00291 | 0.0418 |
| CCL23 | -18.59 | -4.22 | 5.15 | 95.68 | 0.00315 | 0.0418 |
| ATP1A2 | -18.59 | -4.22 | 19.67 | 365.56 | 0.00329 | 0.0418 |
| JAM3 | -18.51 | -4.21 | 166.55 | 3082.3 | 0.00291 | 0.0418 |
| CRISPLD2 | -18.25 | -4.19 | 500.46 | 9133.06 | 0.00424 | 0.0418 |
| LOC284276 | -18.25 | -4.19 | 11.26 | 205.39 | 0.00442 | 0.0418 |
| RBPMS2 | -18.07 | -4.18 | 65.61 | 1185.58 | 0.00291 | 0.0418 |
| MYCT1 | -18.03 | -4.17 | 44.11 | 795.17 | 0.00297 | 0.0418 |
| FILIP1 | -18.02 | -4.17 | 21.67 | 390.4 | 0.005 | 0.0418 |
| EBF1 | -17.9 | -4.16 | 45.98 | 823.23 | 0.00291 | 0.0418 |
| PPP1R12B | -17.89 | -4.16 | 953.46 | 17054.92 | 0.00291 | 0.0418 |
| HSPA12B | -17.72 | -4.15 | 54.54 | 966.42 | 0.00291 | 0.0418 |
| CLDN5 | -17.62 | -4.14 | 160.81 | 2832.91 | 0.0033 | 0.0418 |
| NEGR1 | -17.68 | -4.14 | 47.8 | 845.36 | 0.00337 | 0.0418 |
| CD34 | -17.47 | -4.13 | 433.42 | 7570.87 | 0.00291 | 0.0418 |
| EMILIN1 | -17.49 | -4.13 | 607.12 | 10615.9 | 0.00291 | 0.0418 |
| ITM2A | -17.57 | -4.13 | 153.03 | 2688.33 | 0.00291 | 0.0418 |
| CSRP1 | -17.42 | -4.12 | 4631.89 | 80707.8 | 0.00291 | 0.0418 |
| TPM2 | -17.34 | -4.12 | 3576.25 | 62026.96 | 0.00304 | 0.0418 |
| MAOB | -17.39 | -4.12 | 253.89 | 4415.1 | 0.0031 | 0.0418 |
| ZFPM2 | -17.43 | -4.12 | 39.33 | 685.47 | 0.00391 | 0.0418 |
| TGFBR3 | -17.11 | -4.1 | 260.11 | 4450.71 | 0.0031 | 0.0418 |
| PRTG | -17.17 | -4.1 | 13.19 | 226.6 | 0.00661 | 0.043 |
| EDNRB | -17.04 | -4.09 | 53.35 | 909.26 | 0.0031 | 0.0418 |
| GNA14 | -17.06 | -4.09 | 34.09 | 581.64 | 0.00399 | 0.0418 |
| SRPX | -16.92 | -4.08 | 84.37 | 1427.61 | 0.00317 | 0.0418 |
| PDZRN4 | -16.94 | -4.08 | 4.39 | 74.39 | 0.00347 | 0.0418 |
| LRRC4B | -16.94 | -4.08 | 30.26 | 512.74 | 0.00359 | 0.0418 |
| NKAPL | -16.79 | -4.07 | 1.94 | 32.52 | 0.00301 | 0.0418 |
| NPY1R | -16.81 | -4.07 | 18.75 | 315.06 | 0.00389 | 0.0418 |
| KCNJ8 | -16.64 | -4.06 | 47.14 | 784.59 | 0.00291 | 0.0418 |
| SLC8A1 | -16.57 | -4.05 | 103.87 | 1720.75 | 0.00291 | 0.0418 |
| JCAD | -16.47 | -4.04 | 201.94 | 3325.99 | 0.00323 | 0.0418 |
| ST6GALNAC3 | -16.31 | -4.03 | 13.56 | 221.18 | 0.0033 | 0.0418 |
| ACTC1 | -16.28 | -4.03 | 17.89 | 291.33 | 0.00358 | 0.0418 |
| PDE1A | -16.27 | -4.02 | 19.52 | 317.55 | 0.00304 | 0.0418 |
| LRCH2 | -16.18 | -4.02 | 25.45 | 411.94 | 0.00317 | 0.0418 |
| NR4A3 | -16.21 | -4.02 | 124.19 | 2013.17 | 0.00992 | 0.0488 |
| TNS1 | -16.12 | -4.01 | 1284.49 | 20710.9 | 0.00291 | 0.0418 |
| ACOX2 | -16.08 | -4.01 | 23.41 | 376.49 | 0.0033 | 0.0418 |
| MITF | -15.97 | -4 | 192.71 | 3077.16 | 0.00291 | 0.0418 |
| IL17B | -16.01 | -4 | 1.67 | 26.8 | 0.00317 | 0.0418 |
| FILIP1L | -15.99 | -4 | 364.65 | 5832.13 | 0.0033 | 0.0418 |
| SLC2A4 | -15.89 | -3.99 | 20.38 | 323.85 | 0.00291 | 0.0418 |
| SVEP1 | -15.92 | -3.99 | 152.02 | 2420.65 | 0.0033 | 0.0418 |
| TEK | -15.76 | -3.98 | 56.92 | 897.3 | 0.00291 | 0.0418 |
| CCDC80 | -15.76 | -3.98 | 1776.77 | 28006.28 | 0.00345 | 0.0418 |
| CTTNBP2 | -15.77 | -3.98 | 53.93 | 850.43 | 0.00399 | 0.0418 |
| ABCB1 | -15.62 | -3.97 | 56.93 | 889.27 | 0.00337 | 0.0418 |
| SLC45A1 | -15.53 | -3.96 | 16.69 | 259.26 | 0.00291 | 0.0418 |
| MYRIP | -15.57 | -3.96 | 26.63 | 414.69 | 0.0033 | 0.0418 |
| SLC35F4 | -15.53 | -3.96 | 0.69 | 10.68 | 0.00332 | 0.0418 |
| RUNX1T1 | -15.58 | -3.96 | 5.87 | 91.41 | 0.00497 | 0.0418 |
| DIXDC1 | -15.41 | -3.95 | 138.73 | 2137.64 | 0.00291 | 0.0418 |
| ARHGEF25 | -15.46 | -3.95 | 164.41 | 2542.32 | 0.00291 | 0.0418 |
| TSPAN2 | -15.43 | -3.95 | 111.33 | 1717.66 | 0.0031 | 0.0418 |
| SERP2 | -15.37 | -3.94 | 11.46 | 176.16 | 0.00291 | 0.0418 |
| ECM2 | -15.32 | -3.94 | 74.17 | 1136.58 | 0.00291 | 0.0418 |
| USHBP1 | -15.38 | -3.94 | 16.83 | 258.77 | 0.00304 | 0.0418 |
| SSC5D | -15.39 | -3.94 | 186.19 | 2865.79 | 0.0033 | 0.0418 |
| FMN2 | -15.21 | -3.93 | 3.88 | 59.12 | 0.00243 | 0.0418 |
| TGFB1I1 | -15.23 | -3.93 | 423.45 | 6448.96 | 0.00291 | 0.0418 |
| ZCCHC24 | -15.22 | -3.93 | 254.24 | 3869.64 | 0.00291 | 0.0418 |
| ZNF781 | -15.23 | -3.93 | 6.82 | 103.92 | 0.0031 | 0.0418 |
| APOD | -15.22 | -3.93 | 282.55 | 4299.83 | 0.00367 | 0.0418 |
| ZEB1 | -15.13 | -3.92 | 222.22 | 3362.37 | 0.00297 | 0.0418 |
| ADAMTS1 | -15.09 | -3.92 | 538.62 | 8126.06 | 0.00304 | 0.0418 |
| PTCHD1 | -15.18 | -3.92 | 8.02 | 121.82 | 0.00344 | 0.0418 |
| ZNF835 | -15.15 | -3.92 | 2.64 | 40.04 | 0.00389 | 0.0418 |
| WDR17 | -15.11 | -3.92 | 3.08 | 46.53 | 0.00417 | 0.0418 |
| SLIT3 | -15.05 | -3.91 | 144.41 | 2172.97 | 0.00337 | 0.0418 |
| ZNF208 | -14.96 | -3.9 | 6.77 | 101.24 | 0.00351 | 0.0418 |
| ALKAL2 | -14.89 | -3.9 | 27.61 | 411.04 | 0.00518 | 0.0418 |
| PCDHA3 | -14.87 | -3.89 | 8.97 | 133.39 | 0.00322 | 0.0418 |
| PDE7B | -14.87 | -3.89 | 48.02 | 713.96 | 0.00375 | 0.0418 |
| APBB1 | -14.76 | -3.88 | 190.47 | 2811.99 | 0.00291 | 0.0418 |
| KLF15 | -14.76 | -3.88 | 31.26 | 461.31 | 0.0031 | 0.0418 |
| OSR2 | -14.73 | -3.88 | 327.25 | 4819.92 | 0.0031 | 0.0418 |
| GNG7 | -14.71 | -3.88 | 39.55 | 581.73 | 0.00317 | 0.0418 |
| METTL24 | -14.69 | -3.88 | 2.54 | 37.36 | 0.00333 | 0.0418 |
| DIRAS1 | -14.68 | -3.88 | 46.06 | 675.99 | 0.00345 | 0.0418 |
| ZNF677 | -14.59 | -3.87 | 6.36 | 92.81 | 0.00323 | 0.0418 |
| CHRD | -14.49 | -3.86 | 115.77 | 1677.39 | 0.00304 | 0.0418 |
| ANTXR2 | -14.48 | -3.86 | 456.58 | 6610.03 | 0.0031 | 0.0418 |
| KDR | -14.48 | -3.86 | 214.01 | 3099.07 | 0.0048 | 0.0418 |
| CAMK2A | -14.43 | -3.85 | 15.45 | 222.96 | 0.00531 | 0.0418 |
| ARHGEF15 | -14.36 | -3.84 | 77.55 | 1113.83 | 0.00291 | 0.0418 |
| DENND2A | -14.29 | -3.84 | 94.26 | 1347.4 | 0.00291 | 0.0418 |
| FERMT2 | -14.29 | -3.84 | 388.94 | 5556.14 | 0.00291 | 0.0418 |
| AVPR1A | -14.35 | -3.84 | 7.86 | 112.75 | 0.00396 | 0.0418 |
| FAM13C | -14.21 | -3.83 | 20.56 | 292.15 | 0.0051 | 0.0418 |
| PRKG1 | -14.22 | -3.83 | 11.18 | 158.92 | 0.00884 | 0.0471 |
| **Gene** | **Fold change** | **log2 (Fold change)** | **Mean TPM* (tumor)** | **Mean TPM* (normal)** | **p-value** | **adjusted p-value** |
| RHOJ | -14.14 | -3.82 | 41.71 | 589.89 | 0.00297 | 0.0418 |
| CADM3 | -14.1 | -3.82 | 24.93 | 351.61 | 0.00366 | 0.0418 |
| DUSP26 | -14.16 | -3.82 | 7.2 | 101.97 | 0.00898 | 0.0474 |
| RSPH9 | -14.04 | -3.81 | 4.49 | 63.09 | 0.00344 | 0.0418 |
| POPDC2 | -14.06 | -3.81 | 32.22 | 453.05 | 0.00391 | 0.0418 |
| CPXM2 | -14.01 | -3.81 | 101.95 | 1428.79 | 0.00424 | 0.0418 |
| NEXN | -13.93 | -3.8 | 168.3 | 2345.11 | 0.0033 | 0.0418 |
| MAGEL2 | -13.95 | -3.8 | 6.59 | 91.87 | 0.00336 | 0.0418 |
| COX7A1 | -13.94 | -3.8 | 59.32 | 826.62 | 0.00337 | 0.0418 |
| OLFML1 | -13.86 | -3.79 | 73.14 | 1013.84 | 0.00291 | 0.0418 |
| FAXDC2 | -13.88 | -3.79 | 156.76 | 2175.92 | 0.00304 | 0.0418 |
| GRASP | -13.82 | -3.79 | 96.25 | 1330.69 | 0.00304 | 0.0418 |
| KCNN2 | -13.83 | -3.79 | 5.34 | 73.89 | 0.00374 | 0.0418 |
| TMEM200B | -13.69 | -3.78 | 184.45 | 2525.23 | 0.00291 | 0.0418 |
| NACAD | -13.71 | -3.78 | 44.06 | 604.09 | 0.00304 | 0.0418 |
| GRID1 | -13.76 | -3.78 | 18.71 | 257.47 | 0.00323 | 0.0418 |
| LMO3 | -13.72 | -3.78 | 109.44 | 1501.37 | 0.00375 | 0.0418 |
| C16orf45 | -13.64 | -3.77 | 128.11 | 1747.83 | 0.00291 | 0.0418 |
| ABCA6 | -13.62 | -3.77 | 17.69 | 240.85 | 0.0031 | 0.0418 |
| AQP1 | -13.59 | -3.76 | 1149.2 | 15620.02 | 0.0031 | 0.0418 |
| NUDT10 | -13.52 | -3.76 | 20.06 | 271.31 | 0.00337 | 0.0418 |
| NAALAD2 | -13.43 | -3.75 | 10.05 | 134.9 | 0.00304 | 0.0418 |
| ZNF135 | -13.5 | -3.75 | 19.37 | 261.49 | 0.00399 | 0.0418 |
| SOD3 | -13.35 | -3.74 | 241.92 | 3230.39 | 0.00304 | 0.0418 |
| ABI3BP | -13.37 | -3.74 | 154.43 | 2064.46 | 0.00345 | 0.0418 |
| DCLK1 | -13.36 | -3.74 | 49.63 | 663.07 | 0.00542 | 0.0418 |
| RECK | -13.25 | -3.73 | 82.6 | 1094.31 | 0.00304 | 0.0418 |
| KCNMB2 | -13.25 | -3.73 | 7.89 | 104.51 | 0.0031 | 0.0418 |
| SYPL2 | -13.25 | -3.73 | 11.83 | 156.71 | 0.00317 | 0.0418 |
| LOC401093 | -13.27 | -3.73 | 92.88 | 1232.45 | 0.00317 | 0.0418 |
| ZNF521 | -13.26 | -3.73 | 58.32 | 773.56 | 0.00383 | 0.0418 |
| RCVRN | -13.21 | -3.72 | 1.04 | 13.81 | 0.00303 | 0.0418 |
| FAM107A | -13.18 | -3.72 | 218.75 | 2882.45 | 0.00433 | 0.0418 |
| RAB3IL1 | -13.12 | -3.71 | 142.71 | 1872.19 | 0.00291 | 0.0418 |
| ADGRA2 | -13.07 | -3.71 | 381.7 | 4988.49 | 0.00304 | 0.0418 |
| GLB1L3 | -12.96 | -3.7 | 3.55 | 45.99 | 0.00698 | 0.0434 |
| GATA5 | -12.89 | -3.69 | 8.84 | 113.88 | 0.00284 | 0.0418 |
| PCDHGA6 | -12.88 | -3.69 | 8.38 | 107.88 | 0.00531 | 0.0418 |
| PDE1B | -12.84 | -3.68 | 33.39 | 428.75 | 0.0031 | 0.0418 |
| ITPR1 | -12.78 | -3.68 | 272.91 | 3489.21 | 0.00317 | 0.0418 |
| RIPOR2 | -12.77 | -3.68 | 89.27 | 1140.39 | 0.00367 | 0.0418 |
| RNF180 | -12.83 | -3.68 | 20.11 | 258 | 0.00367 | 0.0418 |
| TMEM200A | -12.81 | -3.68 | 93.27 | 1194.65 | 0.00367 | 0.0418 |
| TIMP3 | -12.77 | -3.67 | 2956.31 | 37757.31 | 0.00291 | 0.0418 |
| IL17D | -12.69 | -3.67 | 10.68 | 135.49 | 0.0033 | 0.0418 |
| LOC283174 | -12.73 | -3.67 | 36.34 | 462.55 | 0.00367 | 0.0418 |
| FGF2 | -12.76 | -3.67 | 97.57 | 1245.48 | 0.00367 | 0.0418 |
| KLF17 | -12.61 | -3.66 | 2.04 | 25.72 | 0.00273 | 0.0418 |
| CCM2L | -12.66 | -3.66 | 36.04 | 456.26 | 0.00297 | 0.0418 |
| FAM218A | -12.63 | -3.66 | 2.54 | 32.08 | 0.00352 | 0.0418 |
| S1PR1 | -12.58 | -3.65 | 136.91 | 1722.32 | 0.00291 | 0.0418 |
| TMEM88 | -12.59 | -3.65 | 25.86 | 325.64 | 0.00323 | 0.0418 |
| KCNA5 | -12.52 | -3.65 | 5.15 | 64.54 | 0.00344 | 0.0418 |
| ITGA7 | -12.57 | -3.65 | 174.6 | 2195.41 | 0.00359 | 0.0418 |
| PDE11A | -12.52 | -3.65 | 9.57 | 119.78 | 0.0057 | 0.0421 |
| NOSTRIN | -12.51 | -3.64 | 60.77 | 760.01 | 0.0033 | 0.0418 |
| TXLNB | -12.43 | -3.64 | 15.45 | 192.08 | 0.00359 | 0.0418 |
| EFHA2 | -12.44 | -3.64 | 14.88 | 185.14 | 0.00383 | 0.0418 |
| LEPR | -12.36 | -3.63 | 162.55 | 2009.06 | 0.00291 | 0.0418 |
| LIFR | -12.35 | -3.63 | 336.34 | 4152.96 | 0.0033 | 0.0418 |
| RGN | -12.39 | -3.63 | 23.43 | 290.28 | 0.00337 | 0.0418 |
| HOXA11 | -12.39 | -3.63 | 153.22 | 1899.18 | 0.00352 | 0.0418 |
| BEX1 | -12.4 | -3.63 | 15.97 | 198.07 | 0.00451 | 0.0418 |
| EGFL7 | -12.3 | -3.62 | 317.27 | 3903.39 | 0.00304 | 0.0418 |
| PRKN | -12.3 | -3.62 | 9.87 | 121.31 | 0.0031 | 0.0418 |
| ESR1 | -12.31 | -3.62 | 696.25 | 8570.12 | 0.00352 | 0.0418 |
| EFNB3 | -12.27 | -3.62 | 38.83 | 476.34 | 0.00542 | 0.0418 |
| KY | -12.31 | -3.62 | 11.49 | 141.42 | 0.00701 | 0.0434 |
| COLEC12 | -12.23 | -3.61 | 97.92 | 1197.49 | 0.0033 | 0.0418 |
| EPO | -12.17 | -3.61 | 2.59 | 31.52 | 0.0038 | 0.0418 |
| CYS1 | -12.08 | -3.59 | 86.52 | 1045.08 | 0.00337 | 0.0418 |
| KLF2 | -12.07 | -3.59 | 237.14 | 2861.63 | 0.00337 | 0.0418 |
| AFF3 | -12.01 | -3.59 | 30.9 | 370.94 | 0.00352 | 0.0418 |
| LOC399959 | -12.08 | -3.59 | 121.71 | 1470.14 | 0.00375 | 0.0418 |
| ITIH5 | -12.04 | -3.59 | 207.52 | 2497.61 | 0.00391 | 0.0418 |
| HAPLN2 | -12.02 | -3.59 | 1.88 | 22.64 | 0.00554 | 0.0419 |
| OR51E2 | -12.08 | -3.59 | 4.17 | 50.32 | 0.00892 | 0.0474 |
| TSPAN18 | -11.94 | -3.58 | 190.36 | 2273.58 | 0.0051 | 0.0418 |
| LOC144571 | -11.84 | -3.57 | 13.36 | 158.24 | 0.00291 | 0.0418 |
| CYYR1 | -11.84 | -3.57 | 110.71 | 1311.23 | 0.00297 | 0.0418 |
| MATN2 | -11.87 | -3.57 | 873.97 | 10372.85 | 0.00297 | 0.0418 |
| ZSCAN18 | -11.91 | -3.57 | 212.49 | 2530.56 | 0.0031 | 0.0418 |
| PCDHGA5 | -11.9 | -3.57 | 5.62 | 66.88 | 0.00358 | 0.0418 |
| C16orf89 | -11.85 | -3.57 | 42.29 | 501.24 | 0.00535 | 0.0418 |
| DDR2 | -11.9 | -3.57 | 34.32 | 408.39 | 0.00623 | 0.0425 |
| KANK2 | -11.81 | -3.56 | 1272.31 | 15023.77 | 0.00291 | 0.0418 |
| PDGFRA | -11.78 | -3.56 | 397.19 | 4680.81 | 0.00304 | 0.0418 |
| CTGF | -11.76 | -3.56 | 2218.83 | 26103.73 | 0.0033 | 0.0418 |
| GRIK5 | -11.81 | -3.56 | 23.98 | 283.2 | 0.0033 | 0.0418 |
| TM4SF18 | -11.77 | -3.56 | 17.28 | 203.39 | 0.00352 | 0.0418 |
| FAM110B | -11.77 | -3.56 | 37.98 | 446.91 | 0.00367 | 0.0418 |
| EXTL1 | -11.81 | -3.56 | 4.02 | 47.51 | 0.00382 | 0.0418 |
| RYR3 | -11.76 | -3.56 | 56.69 | 666.61 | 0.00542 | 0.0418 |
| ART5 | -11.83 | -3.56 | 8.36 | 98.95 | 0.00588 | 0.0421 |
| CALD1 | -11.71 | -3.55 | 2685.57 | 31437.01 | 0.00291 | 0.0418 |
| C18orf34 | -11.7 | -3.55 | 2.93 | 34.3 | 0.00359 | 0.0418 |
| C15orf51 | -11.64 | -3.54 | 1.49 | 17.33 | 0.00287 | 0.0418 |
| TMEM47 | -11.61 | -3.54 | 253.97 | 2947.87 | 0.00304 | 0.0418 |
| CCDC36 | -11.64 | -3.54 | 2.59 | 30.1 | 0.00479 | 0.0418 |
| SLIT2 | -11.67 | -3.54 | 63.28 | 738.55 | 0.00599 | 0.0421 |
| KIAA1755 | -11.52 | -3.53 | 38.3 | 441.16 | 0.00297 | 0.0418 |
| PMP22 | -11.58 | -3.53 | 563.76 | 6527.9 | 0.0031 | 0.0418 |
| ZNF385D | -11.57 | -3.53 | 3.74 | 43.25 | 0.00395 | 0.0418 |
| NRXN2 | -11.5 | -3.52 | 66.77 | 767.6 | 0.00323 | 0.0418 |
| MUM1L1 | -11.48 | -3.52 | 69.43 | 796.93 | 0.0033 | 0.0418 |
| ECSCR | -11.48 | -3.52 | 40.62 | 466.31 | 0.00345 | 0.0418 |
| ACSS3 | -11.5 | -3.52 | 33 | 379.59 | 0.00407 | 0.0418 |
| FGF13 | -11.48 | -3.52 | 35.51 | 407.57 | 0.00461 | 0.0418 |
| TIE1 | -11.41 | -3.51 | 170.41 | 1943.91 | 0.00291 | 0.0418 |
| EPDR1 | -11.41 | -3.51 | 130.57 | 1489.33 | 0.00304 | 0.0418 |
| NPR1 | -11.37 | -3.51 | 168.1 | 1911.8 | 0.00416 | 0.0418 |
| TCEAL1 | -11.33 | -3.5 | 231.59 | 2624.45 | 0.00291 | 0.0418 |
| YPEL4 | -11.25 | -3.49 | 15.2 | 170.99 | 0.00317 | 0.0418 |
| LDB3 | -11.25 | -3.49 | 29.6 | 332.9 | 0.00383 | 0.0418 |
| FLJ36777 | -11.14 | -3.48 | 2.83 | 31.53 | 0.00297 | 0.0418 |
| VIP | -11.15 | -3.48 | 1.18 | 13.14 | 0.00322 | 0.0418 |
| TPM1 | -11.19 | -3.48 | 3494.68 | 39107.13 | 0.0033 | 0.0418 |
| MEF2C | -11.09 | -3.47 | 144.99 | 1608.24 | 0.00291 | 0.0418 |
| CLEC14A | -11.08 | -3.47 | 155.35 | 1720.59 | 0.00297 | 0.0418 |
| ZNF483 | -11.09 | -3.47 | 6.68 | 74.11 | 0.00375 | 0.0418 |
| PRR16 | -11.07 | -3.47 | 31.5 | 348.73 | 0.00399 | 0.0418 |
| ADRA2C | -11.06 | -3.47 | 170.8 | 1889.48 | 0.00407 | 0.0418 |
| PLCXD3 | -11 | -3.46 | 14.86 | 163.46 | 0.00296 | 0.0418 |
| SMTN | -11.03 | -3.46 | 1033.75 | 11405.18 | 0.00297 | 0.0418 |
| CGNL1 | -11.01 | -3.46 | 211.13 | 2323.94 | 0.00323 | 0.0418 |
| HEYL | -11.02 | -3.46 | 167.86 | 1849.77 | 0.00345 | 0.0418 |
| PCDHB5 | -10.99 | -3.46 | 20.11 | 221.09 | 0.00391 | 0.0418 |
| ADRA1D | -11.01 | -3.46 | 7.88 | 86.84 | 0.00398 | 0.0418 |
| HSD17B6 | -11.02 | -3.46 | 85.22 | 939.34 | 0.00399 | 0.0418 |
| PLA2G5 | -11.01 | -3.46 | 4.29 | 47.21 | 0.00482 | 0.0418 |
| VIPR2 | -10.99 | -3.46 | 48.63 | 534.22 | 0.00563 | 0.0419 |
| MORN5 | -10.9 | -3.45 | 8.39 | 91.49 | 0.00633 | 0.0427 |
| ARMCX1 | -10.83 | -3.44 | 122.25 | 1323.86 | 0.00291 | 0.0418 |
| NRIP2 | -10.86 | -3.44 | 36.48 | 396.35 | 0.00304 | 0.0418 |
| PCDHB4 | -10.84 | -3.44 | 24.51 | 265.57 | 0.00383 | 0.0418 |
| CNTN4 | -10.85 | -3.44 | 36.44 | 395.51 | 0.00416 | 0.0418 |
| ROBO4 | -10.78 | -3.43 | 160.73 | 1731.87 | 0.00291 | 0.0418 |
| MMRN2 | -10.72 | -3.42 | 271.24 | 2908.03 | 0.00297 | 0.0418 |
| SYNE1 | -10.72 | -3.42 | 315.83 | 3386.62 | 0.00297 | 0.0418 |
| C1orf229 | -10.72 | -3.42 | 2.52 | 26.99 | 0.00356 | 0.0418 |
| RGS2 | -10.72 | -3.42 | 766.15 | 8214.36 | 0.00359 | 0.0418 |
| GALNTL2 | -10.7 | -3.42 | 33.35 | 356.79 | 0.00416 | 0.0418 |
| REM1 | -10.6 | -3.41 | 20.91 | 221.59 | 0.00304 | 0.0418 |
| BMX | -10.6 | -3.41 | 11.37 | 120.46 | 0.00337 | 0.0418 |
| VSTM4 | -10.66 | -3.41 | 125.16 | 1334.79 | 0.00367 | 0.0418 |
| ZNF626 | -10.63 | -3.41 | 24.94 | 265.23 | 0.00399 | 0.0418 |
| ADGRL4 | -10.57 | -3.4 | 143.02 | 1511.85 | 0.00297 | 0.0418 |
| PKNOX2 | -10.57 | -3.4 | 20.46 | 216.29 | 0.00433 | 0.0418 |
| NAALADL1 | -10.45 | -3.39 | 36.64 | 383.03 | 0.00291 | 0.0418 |
| TUB | -10.48 | -3.39 | 95.99 | 1005.92 | 0.00323 | 0.0418 |
| TAL1 | -10.49 | -3.39 | 24.03 | 252.23 | 0.0033 | 0.0418 |
| PCDHB7 | -10.5 | -3.39 | 30.23 | 317.43 | 0.00352 | 0.0418 |
| HIGD1B | -10.39 | -3.38 | 7.63 | 79.3 | 0.00304 | 0.0418 |
| IGFBP5 | -10.41 | -3.38 | 4005.65 | 41704.91 | 0.00442 | 0.0418 |
| FMOD | -10.32 | -3.37 | 596.48 | 6154.05 | 0.00317 | 0.0418 |
| DSCAML1 | -10.33 | -3.37 | 13.2 | 136.38 | 0.00787 | 0.0452 |
| GALNT13 | -10.36 | -3.37 | 5.08 | 52.59 | 0.00855 | 0.0466 |
| ADCY4 | -10.27 | -3.36 | 79.51 | 816.91 | 0.00304 | 0.0418 |
| DCHS1 | -10.23 | -3.36 | 334.59 | 3423.6 | 0.00304 | 0.0418 |
| ITGA9 | -10.27 | -3.36 | 36.63 | 376.05 | 0.00359 | 0.0418 |
| STK33 | -10.27 | -3.36 | 26.21 | 269.28 | 0.00415 | 0.0418 |
| GREB1 | -10.26 | -3.36 | 233.62 | 2396.19 | 0.00416 | 0.0418 |
| KANK3 | -10.22 | -3.35 | 64.46 | 658.98 | 0.00291 | 0.0418 |
| HIPK4 | -10.23 | -3.35 | 2.03 | 20.74 | 0.00309 | 0.0418 |
| FAM198A | -10.2 | -3.35 | 36.44 | 371.59 | 0.00367 | 0.0418 |
| PHF21B | -10.19 | -3.35 | 8.63 | 87.96 | 0.00548 | 0.0418 |
| LHFPL6 | -10.15 | -3.34 | 289.61 | 2939.2 | 0.00291 | 0.0418 |
| FAM162B | -10.1 | -3.34 | 11.87 | 119.9 | 0.00323 | 0.0418 |
| SELM | -10.16 | -3.34 | 747.02 | 7588.16 | 0.00352 | 0.0418 |
| PID1 | -10.13 | -3.34 | 103.45 | 1048.28 | 0.00407 | 0.0418 |
| KLF9 | -10.03 | -3.33 | 496.23 | 4979.3 | 0.00291 | 0.0418 |
| ZNF423 | -10.05 | -3.33 | 48.88 | 491.19 | 0.00391 | 0.0418 |
| LRFN5 | -10.03 | -3.33 | 12.06 | 120.91 | 0.0045 | 0.0418 |
| PRLR | -10.06 | -3.33 | 185.39 | 1864.89 | 0.00451 | 0.0418 |
| HSD17B13 | -9.98 | -3.32 | 14.53 | 145.01 | 0.00398 | 0.0418 |
| **Gene** | **Fold change** | **log2 (Fold change)** | **Mean TPM* (tumor)** | **Mean TPM* (normal)** | **p-value** | **adjusted p-value** |
| KERA | -9.98 | -3.32 | 0.9 | 8.99 | 0.00621 | 0.0425 |
| TNS2 | -9.94 | -3.31 | 611.5 | 6078.82 | 0.00291 | 0.0418 |
| CFL2 | -9.91 | -3.31 | 401.73 | 3981.47 | 0.00297 | 0.0418 |
| PHYHIP | -9.91 | -3.31 | 48.63 | 482.08 | 0.00352 | 0.0418 |
| EDA2R | -9.9 | -3.31 | 13.28 | 131.48 | 0.00451 | 0.0418 |
| ATP6V1G2 | -9.92 | -3.31 | 8.1 | 80.34 | 0.0052 | 0.0418 |
| CLU | -9.82 | -3.3 | 5259.55 | 51672.5 | 0.00359 | 0.0418 |
| ERVFRDE1 | -9.87 | -3.3 | 1.26 | 12.41 | 0.00402 | 0.0418 |
| STUM | -9.82 | -3.3 | 6.04 | 59.31 | 0.00592 | 0.0421 |
| GYPC | -9.76 | -3.29 | 189.23 | 1847.19 | 0.00291 | 0.0418 |
| SERPINF1 | -9.78 | -3.29 | 1238.42 | 12117.4 | 0.00297 | 0.0418 |
| STON1 | -9.78 | -3.29 | 210.85 | 2062.49 | 0.00317 | 0.0418 |
| LOC100128239 | -9.75 | -3.29 | 2.08 | 20.28 | 0.00342 | 0.0418 |
| PCDHGA12 | -9.78 | -3.29 | 7.59 | 74.27 | 0.00499 | 0.0418 |
| C13orf33 | -9.76 | -3.29 | 76.5 | 747.01 | 0.00937 | 0.0479 |
| EFEMP2 | -9.69 | -3.28 | 469.93 | 4552.28 | 0.00291 | 0.0418 |
| LTBP4 | -9.72 | -3.28 | 2267.6 | 22042.88 | 0.00291 | 0.0418 |
| NPR2 | -9.74 | -3.28 | 96.64 | 941.26 | 0.00297 | 0.0418 |
| ZNF418 | -9.68 | -3.28 | 10.11 | 97.88 | 0.00416 | 0.0418 |
| PCDH20 | -9.71 | -3.28 | 13.81 | 134.16 | 0.00469 | 0.0418 |
| LRRC32 | -9.63 | -3.27 | 268.66 | 2586.23 | 0.00317 | 0.0418 |
| NGF | -9.64 | -3.27 | 12.61 | 121.63 | 0.00415 | 0.0418 |
| A2M | -9.59 | -3.26 | 3456.41 | 33134.77 | 0.0031 | 0.0418 |
| PCDHGB7 | -9.56 | -3.26 | 27.52 | 263.24 | 0.00442 | 0.0418 |
| PAMR1 | -9.57 | -3.26 | 176.43 | 1687.91 | 0.00531 | 0.0418 |
| SGCD | -9.51 | -3.25 | 69.85 | 664.38 | 0.00416 | 0.0418 |
| ZC3H12B | -9.42 | -3.24 | 14.95 | 140.83 | 0.00291 | 0.0418 |
| ATP8B2 | -9.48 | -3.24 | 346.87 | 3288.35 | 0.00345 | 0.0418 |
| DAAM2 | -9.44 | -3.24 | 92 | 868.08 | 0.00359 | 0.0418 |
| PGCP | -9.29 | -3.22 | 210.83 | 1957.81 | 0.00291 | 0.0418 |
| HIC1 | -9.31 | -3.22 | 94.87 | 883.27 | 0.0031 | 0.0418 |
| C20orf203 | -9.34 | -3.22 | 0.96 | 8.96 | 0.00347 | 0.0418 |
| RAB9B | -9.32 | -3.22 | 4.45 | 41.46 | 0.00916 | 0.0476 |
| PKIG | -9.25 | -3.21 | 381.25 | 3525.82 | 0.00291 | 0.0418 |
| RTL3 | -9.27 | -3.21 | 0.83 | 7.74 | 0.00296 | 0.0418 |
| JAZF1 | -9.28 | -3.21 | 253.25 | 2351.3 | 0.00297 | 0.0418 |
| SNED1 | -9.25 | -3.21 | 151.96 | 1405.41 | 0.00337 | 0.0418 |
| STXBP5L | -9.26 | -3.21 | 13.48 | 124.86 | 0.00874 | 0.047 |
| SGCE | -9.19 | -3.2 | 186.41 | 1713.04 | 0.00291 | 0.0418 |
| TCEAL3 | -9.22 | -3.2 | 373.9 | 3447.03 | 0.00297 | 0.0418 |
| PCDHB15 | -9.17 | -3.2 | 20.79 | 190.63 | 0.0033 | 0.0418 |
| RCAN2 | -9.2 | -3.2 | 89.49 | 822.99 | 0.00367 | 0.0418 |
| SRL | -9.18 | -3.2 | 22.83 | 209.68 | 0.00399 | 0.0418 |
| C7orf51 | -9.18 | -3.2 | 16.85 | 154.58 | 0.00442 | 0.0418 |
| ACSM1 | -9.16 | -3.2 | 5.8 | 53.14 | 0.00499 | 0.0418 |
| GPR146 | -9.1 | -3.19 | 55.27 | 503.22 | 0.00291 | 0.0418 |
| FGD5 | -9.13 | -3.19 | 163.14 | 1489.18 | 0.00337 | 0.0418 |
| LOC283731 | -9.12 | -3.19 | 3.06 | 27.9 | 0.00495 | 0.0418 |
| SOCS2 | -9.04 | -3.18 | 325.37 | 2940.29 | 0.00317 | 0.0418 |
| SCN4B | -9.09 | -3.18 | 38.07 | 346.26 | 0.00407 | 0.0418 |
| LGI2 | -9.09 | -3.18 | 70.4 | 639.6 | 0.0051 | 0.0418 |
| TIMP2 | -9.02 | -3.17 | 3719.55 | 33544.17 | 0.00291 | 0.0418 |
| TMEM204 | -9.03 | -3.17 | 149.24 | 1347.15 | 0.00291 | 0.0418 |
| HLF | -8.97 | -3.17 | 138.32 | 1241.05 | 0.00304 | 0.0418 |
| ABCA10 | -8.99 | -3.17 | 19.11 | 171.81 | 0.00323 | 0.0418 |
| GCOM1 | -9.01 | -3.17 | 121.23 | 1092.09 | 0.00416 | 0.0418 |
| DIRC3 | -9.03 | -3.17 | 5.12 | 46.27 | 0.00469 | 0.0418 |
| APOLD1 | -8.94 | -3.16 | 229.28 | 2049.79 | 0.0031 | 0.0418 |
| CYP46A1 | -8.93 | -3.16 | 4.84 | 43.23 | 0.00359 | 0.0418 |
| DMD | -8.94 | -3.16 | 172.79 | 1543.93 | 0.00433 | 0.0418 |
| LOC728392 | -8.94 | -3.16 | 141.44 | 1264.43 | 0.00564 | 0.0419 |
| PCDHGA3 | -8.92 | -3.16 | 2.9 | 25.9 | 0.00796 | 0.0454 |
| NAV3 | -8.93 | -3.16 | 16.02 | 143.12 | 0.00804 | 0.0454 |
| EDNRA | -8.88 | -3.15 | 244.25 | 2167.86 | 0.00323 | 0.0418 |
| LAMA4 | -8.9 | -3.15 | 611.1 | 5435.85 | 0.00323 | 0.0418 |
| MYADM | -8.87 | -3.15 | 1892.19 | 16774.89 | 0.0033 | 0.0418 |
| CCIN | -8.91 | -3.15 | 3.55 | 31.62 | 0.00861 | 0.0467 |
| MXRA7 | -8.83 | -3.14 | 1182.91 | 10447.09 | 0.00304 | 0.0418 |
| ISLR | -8.78 | -3.14 | 1304.62 | 11460.99 | 0.00323 | 0.0418 |
| FAM198B | -8.79 | -3.14 | 338.89 | 2977.57 | 0.00345 | 0.0418 |
| GADD45G | -8.83 | -3.14 | 154.63 | 1365.94 | 0.00375 | 0.0418 |
| PPFIA2 | -8.8 | -3.14 | 2.45 | 21.58 | 0.00402 | 0.0418 |
| MCHR1 | -8.79 | -3.14 | 4.6 | 40.48 | 0.00449 | 0.0418 |
| PCDHA10 | -8.82 | -3.14 | 16.74 | 147.67 | 0.00456 | 0.0418 |
| ZNF853 | -8.75 | -3.13 | 106.88 | 935.45 | 0.00304 | 0.0418 |
| ISLR2 | -8.74 | -3.13 | 28.85 | 252.06 | 0.00399 | 0.0418 |
| GUCY1A2 | -8.67 | -3.12 | 11.72 | 101.62 | 0.00451 | 0.0418 |
| TIMP4 | -8.68 | -3.12 | 8.31 | 72.18 | 0.0068 | 0.0433 |
| SYTL4 | -8.61 | -3.11 | 336.46 | 2898.34 | 0.00291 | 0.0418 |
| PTGER2 | -8.65 | -3.11 | 53.61 | 463.8 | 0.00317 | 0.0418 |
| TRPC1 | -8.66 | -3.11 | 66.15 | 572.88 | 0.00323 | 0.0418 |
| BAG2 | -8.61 | -3.11 | 254.23 | 2188.95 | 0.00407 | 0.0418 |
| FAM19A5 | -8.64 | -3.11 | 44.13 | 381.2 | 0.00868 | 0.0467 |
| SLC24A3 | -8.6 | -3.1 | 298.46 | 2567.63 | 0.00291 | 0.0418 |
| CALHM2 | -8.58 | -3.1 | 276.62 | 2374.02 | 0.00297 | 0.0418 |
| DLC1 | -8.57 | -3.1 | 202.38 | 1733.98 | 0.00323 | 0.0418 |
| FRZB | -8.55 | -3.1 | 174.45 | 1490.86 | 0.00352 | 0.0418 |
| CABP1 | -8.6 | -3.1 | 4.64 | 39.94 | 0.00367 | 0.0418 |
| ZNF582 | -8.6 | -3.1 | 8.22 | 70.68 | 0.00367 | 0.0418 |
| CBX7 | -8.5 | -3.09 | 600.89 | 5106.33 | 0.00291 | 0.0418 |
| ESAM | -8.54 | -3.09 | 268.77 | 2295.97 | 0.00297 | 0.0418 |
| NOVA2 | -8.52 | -3.09 | 14.1 | 120.13 | 0.0031 | 0.0418 |
| TMOD1 | -8.54 | -3.09 | 323.15 | 2758.6 | 0.00367 | 0.0418 |
| GLT1D1 | -8.54 | -3.09 | 14.52 | 124.05 | 0.00407 | 0.0418 |
| PCDH18 | -8.49 | -3.09 | 140.11 | 1189.23 | 0.00407 | 0.0418 |
| VWF | -8.48 | -3.08 | 1743.7 | 14778.85 | 0.00304 | 0.0418 |
| THSD7A | -8.46 | -3.08 | 10.88 | 92.03 | 0.00433 | 0.0418 |
| PTPRD | -8.46 | -3.08 | 52.19 | 441.41 | 0.0052 | 0.0418 |
| ANO4 | -8.47 | -3.08 | 17.03 | 144.23 | 0.0097 | 0.0487 |
| STARD8 | -8.4 | -3.07 | 58.11 | 488.33 | 0.00297 | 0.0418 |
| ENOX1 | -8.39 | -3.07 | 21.22 | 178.1 | 0.0033 | 0.0418 |
| LOC339524 | -8.42 | -3.07 | 19.02 | 160.14 | 0.00391 | 0.0418 |
| ABCB5 | -8.41 | -3.07 | 1.6 | 13.5 | 0.00409 | 0.0418 |
| JAKMIP2 | -8.38 | -3.07 | 10.75 | 90.14 | 0.00804 | 0.0454 |
| PKD2 | -8.32 | -3.06 | 404.49 | 3367.23 | 0.00291 | 0.0418 |
| FKBP7 | -8.35 | -3.06 | 110.92 | 926.18 | 0.00304 | 0.0418 |
| ADAMTS8 | -8.35 | -3.06 | 27.34 | 228.25 | 0.00359 | 0.0418 |
| ACVRL1 | -8.28 | -3.05 | 244.42 | 2022.84 | 0.0031 | 0.0418 |
| P2RX1 | -8.29 | -3.05 | 25.58 | 212.14 | 0.0049 | 0.0418 |
| SFRP5 | -8.29 | -3.05 | 8.78 | 72.74 | 0.0053 | 0.0418 |
| OSR1 | -8.3 | -3.05 | 60.18 | 499.51 | 0.00553 | 0.0418 |
| RASA4 | -8.25 | -3.04 | 155.79 | 1285.23 | 0.00291 | 0.0418 |
| RADIL | -8.2 | -3.04 | 29.07 | 238.35 | 0.00359 | 0.0418 |
| AOX1 | -8.24 | -3.04 | 52.89 | 435.78 | 0.00416 | 0.0418 |
| AKAP12 | -8.23 | -3.04 | 495.36 | 4075.77 | 0.00433 | 0.0418 |
| LOC55908 | -8.23 | -3.04 | 0.48 | 4 | 0.0058 | 0.0421 |
| FLT4 | -8.16 | -3.03 | 89.22 | 727.75 | 0.00297 | 0.0418 |
| GEM | -8.19 | -3.03 | 277.19 | 2270.49 | 0.00383 | 0.0418 |
| C10orf107 | -8.18 | -3.03 | 4.41 | 36.13 | 0.00397 | 0.0418 |
| ZC4H2 | -8.15 | -3.03 | 33.39 | 272.07 | 0.00451 | 0.0418 |
| PRSS35 | -8.17 | -3.03 | 6.68 | 54.55 | 0.00476 | 0.0418 |
| SSPN | -8.13 | -3.02 | 220.01 | 1789.07 | 0.00304 | 0.0418 |
| RASGRP2 | -8.11 | -3.02 | 62.82 | 509.39 | 0.00337 | 0.0418 |
| FABP3 | -8.12 | -3.02 | 109.91 | 892.69 | 0.0049 | 0.0418 |
| ALDH1A2 | -8.12 | -3.02 | 329.47 | 2675.46 | 0.00599 | 0.0421 |
| LAMA2 | -8.12 | -3.02 | 187.92 | 1525.23 | 0.00773 | 0.0449 |
| TSPAN7 | -8.04 | -3.01 | 363.31 | 2921.53 | 0.0048 | 0.0418 |
| SYT11 | -7.98 | -3 | 203.17 | 1621.99 | 0.00317 | 0.0418 |
| PRICKLE2 | -8 | -3 | 195.69 | 1564.59 | 0.00323 | 0.0418 |
| PALM | -8.01 | -3 | 278.83 | 2232.98 | 0.00337 | 0.0418 |
| SNCA | -7.98 | -3 | 110.48 | 881.67 | 0.00804 | 0.0454 |
| BCL6B | -7.97 | -2.99 | 124.66 | 993.33 | 0.00304 | 0.0418 |
| PPM1K | -7.96 | -2.99 | 136.3 | 1085.41 | 0.0031 | 0.0418 |
| NOS3 | -7.94 | -2.99 | 126.31 | 1003 | 0.00337 | 0.0418 |
| MXRA8 | -7.92 | -2.99 | 757.44 | 6000.86 | 0.00352 | 0.0418 |
| SEMA3G | -7.95 | -2.99 | 76.22 | 605.79 | 0.00352 | 0.0418 |
| AMPH | -7.93 | -2.99 | 21.31 | 169.08 | 0.00359 | 0.0418 |
| FNDC5 | -7.95 | -2.99 | 18.46 | 146.66 | 0.00391 | 0.0418 |
| GDF10 | -7.94 | -2.99 | 11.4 | 90.47 | 0.00394 | 0.0418 |
| COL6A2 | -7.95 | -2.99 | 5488.43 | 43630.85 | 0.00399 | 0.0418 |
| DTNA | -7.93 | -2.99 | 38.11 | 302.13 | 0.00416 | 0.0418 |
| GIMAP8 | -7.95 | -2.99 | 124.28 | 987.64 | 0.00442 | 0.0418 |
| MAP1A | -7.95 | -2.99 | 136.1 | 1081.39 | 0.0047 | 0.0418 |
| DHH | -7.95 | -2.99 | 3.07 | 24.39 | 0.00509 | 0.0418 |
| STAC2 | -7.94 | -2.99 | 30.07 | 238.62 | 0.0051 | 0.0418 |
| CALCRL | -7.94 | -2.99 | 187.38 | 1488.43 | 0.00542 | 0.0418 |
| TCEAL4 | -7.89 | -2.98 | 1503.55 | 11866.15 | 0.00291 | 0.0418 |
| KLHL10 | -7.9 | -2.98 | 0.81 | 6.39 | 0.00313 | 0.0418 |
| HSD17B14 | -7.82 | -2.97 | 78.29 | 612.07 | 0.00317 | 0.0418 |
| KCNAB1 | -7.84 | -2.97 | 27.02 | 211.91 | 0.00317 | 0.0418 |
| IL17RD | -7.81 | -2.97 | 312.14 | 2438.69 | 0.00367 | 0.0418 |
| ZFP2 | -7.85 | -2.97 | 13.2 | 103.63 | 0.00383 | 0.0418 |
| GPR21 | -7.84 | -2.97 | 1.16 | 9.11 | 0.00777 | 0.0451 |
| LRRC70 | -7.8 | -2.96 | 20.73 | 161.59 | 0.00291 | 0.0418 |
| PECAM1 | -7.78 | -2.96 | 536.37 | 4170.93 | 0.00297 | 0.0418 |
| LOC728819 | -7.77 | -2.96 | 12.01 | 93.28 | 0.00366 | 0.0418 |
| BOC | -7.8 | -2.96 | 354.18 | 2761.83 | 0.0047 | 0.0418 |
| TSPYL5 | -7.79 | -2.96 | 132.53 | 1032.28 | 0.0051 | 0.0418 |
| GARNL3 | -7.71 | -2.95 | 47.67 | 367.48 | 0.00297 | 0.0418 |
| PCDHB18 | -7.72 | -2.95 | 4.43 | 34.24 | 0.00352 | 0.0418 |
| FAT4 | -7.75 | -2.95 | 55.48 | 430.09 | 0.00359 | 0.0418 |
| C14orf139 | -7.73 | -2.95 | 46.51 | 359.66 | 0.00367 | 0.0418 |
| MYOZ3 | -7.75 | -2.95 | 6.76 | 52.4 | 0.00424 | 0.0418 |
| PTCH2 | -7.75 | -2.95 | 14.49 | 112.32 | 0.00424 | 0.0418 |
| PACRG | -7.73 | -2.95 | 9.67 | 74.74 | 0.00556 | 0.0419 |
| DLG2 | -7.75 | -2.95 | 20.36 | 157.67 | 0.00575 | 0.0421 |
| ARHGAP20 | -7.72 | -2.95 | 22.5 | 173.74 | 0.00804 | 0.0454 |
| SCARA3 | -7.7 | -2.94 | 963.11 | 7411.77 | 0.00337 | 0.0418 |
| ACR | -7.7 | -2.94 | 1.62 | 12.52 | 0.00343 | 0.0418 |
| MAPK10 | -7.66 | -2.94 | 101.44 | 777.52 | 0.00352 | 0.0418 |
| RBP5 | -7.69 | -2.94 | 42.57 | 327.18 | 0.00367 | 0.0418 |
| RAB23 | -7.64 | -2.93 | 422.21 | 3226.53 | 0.00304 | 0.0418 |
| PEAR1 | -7.64 | -2.93 | 94.43 | 721.44 | 0.00352 | 0.0418 |
| IGF1 | -7.64 | -2.93 | 271.79 | 2075.98 | 0.00661 | 0.043 |
| RAMP2 | -7.56 | -2.92 | 208.16 | 1572.93 | 0.0031 | 0.0418 |
| **Gene** | **Fold change** | **log2 (Fold change)** | **Mean TPM* (tumor)** | **Mean TPM* (normal)** | **p-value** | **adjusted p-value** |
| AP1S2 | -7.59 | -2.92 | 243.93 | 1852.33 | 0.00345 | 0.0418 |
| PRDM8 | -7.58 | -2.92 | 49.32 | 373.66 | 0.00352 | 0.0418 |
| RNASE4 | -7.58 | -2.92 | 458.95 | 3478.3 | 0.00399 | 0.0418 |
| RGAG4 | -7.57 | -2.92 | 91.16 | 690.13 | 0.00416 | 0.0418 |
| SNAP91 | -7.59 | -2.92 | 2.72 | 20.68 | 0.00437 | 0.0418 |
| ADCY5 | -7.55 | -2.92 | 47.93 | 362.06 | 0.00552 | 0.0418 |
| DIO3OS | -7.55 | -2.92 | 4.64 | 35.04 | 0.00588 | 0.0421 |
| ITGA8 | -7.56 | -2.92 | 21.51 | 162.63 | 0.00598 | 0.0421 |
| PRPH2 | -7.59 | -2.92 | 11.58 | 87.92 | 0.00701 | 0.0434 |
| CD93 | -7.53 | -2.91 | 526.73 | 3966 | 0.00304 | 0.0418 |
| SPEG | -7.51 | -2.91 | 357.56 | 2684.68 | 0.00345 | 0.0418 |
| ZNF665 | -7.5 | -2.91 | 9.71 | 72.82 | 0.00432 | 0.0418 |
| CYBRD1 | -7.46 | -2.9 | 1272.88 | 9492.49 | 0.00291 | 0.0418 |
| RAMP3 | -7.47 | -2.9 | 192.04 | 1433.88 | 0.00352 | 0.0418 |
| NR2F1 | -7.44 | -2.9 | 159.68 | 1187.83 | 0.00433 | 0.0418 |
| ZNF578 | -7.46 | -2.9 | 11.78 | 87.96 | 0.0052 | 0.0418 |
| CCDC184 | -7.46 | -2.9 | 44.46 | 331.51 | 0.00648 | 0.043 |
| SOGA3 | -7.37 | -2.88 | 18.34 | 135.07 | 0.00352 | 0.0418 |
| GATA6 | -7.34 | -2.88 | 150.47 | 1104.95 | 0.00424 | 0.0418 |
| ZNF660 | -7.39 | -2.88 | 2.92 | 21.54 | 0.00769 | 0.0449 |
| CAPN11 | -7.38 | -2.88 | 2.63 | 19.44 | 0.00902 | 0.0474 |
| GPRASP2 | -7.29 | -2.87 | 151.77 | 1106.02 | 0.00304 | 0.0418 |
| GLT8D2 | -7.31 | -2.87 | 122.41 | 894.57 | 0.0031 | 0.0418 |
| TRHDE | -7.31 | -2.87 | 16.84 | 123.1 | 0.00618 | 0.0425 |
| C10orf10 | -7.26 | -2.86 | 732.69 | 5316.76 | 0.0033 | 0.0418 |
| NLGN3 | -7.26 | -2.86 | 23.03 | 167.27 | 0.00383 | 0.0418 |
| SMIM10 | -7.21 | -2.85 | 59.79 | 430.97 | 0.00304 | 0.0418 |
| PLPP3 | -7.22 | -2.85 | 812.29 | 5862.23 | 0.00323 | 0.0418 |
| MEIS2 | -7.22 | -2.85 | 273.1 | 1971.49 | 0.00359 | 0.0418 |
| CD79B | -7.22 | -2.85 | 67.76 | 488.88 | 0.00407 | 0.0418 |
| HEPH | -7.21 | -2.85 | 210.51 | 1517.06 | 0.00407 | 0.0418 |
| PDGFD | -7.23 | -2.85 | 105.97 | 766.28 | 0.00433 | 0.0418 |
| SPON1 | -7.19 | -2.85 | 310.66 | 2235.13 | 0.00553 | 0.0418 |
| MSTN | -7.22 | -2.85 | 3.83 | 27.67 | 0.00711 | 0.0437 |
| C17orf108 | -7.18 | -2.84 | 86.29 | 619.91 | 0.00291 | 0.0418 |
| NFATC4 | -7.14 | -2.84 | 400.13 | 2855.8 | 0.00291 | 0.0418 |
| CC2D2B | -7.14 | -2.84 | 3.06 | 21.83 | 0.0031 | 0.0418 |
| C3orf18 | -7.14 | -2.84 | 51.63 | 368.82 | 0.00345 | 0.0418 |
| TEKT3 | -7.14 | -2.84 | 2.72 | 19.39 | 0.00441 | 0.0418 |
| AKAP6 | -7.17 | -2.84 | 52.86 | 378.96 | 0.0052 | 0.0418 |
| ADCYAP1 | -7.1 | -2.83 | 11.45 | 81.32 | 0.00361 | 0.0418 |
| THBS1 | -7.1 | -2.83 | 3029.87 | 21526.23 | 0.00375 | 0.0418 |
| ADGRF5 | -7.1 | -2.83 | 176.83 | 1255.4 | 0.00416 | 0.0418 |
| RIC3 | -7.09 | -2.83 | 24.89 | 176.52 | 0.00451 | 0.0418 |
| PDZD4 | -7.11 | -2.83 | 72.41 | 515.21 | 0.0049 | 0.0418 |
| TMEM35A | -7.09 | -2.83 | 27.77 | 196.96 | 0.00725 | 0.0439 |
| TBC1D1 | -7.06 | -2.82 | 1081.55 | 7632.59 | 0.00291 | 0.0418 |
| GJA4 | -7.05 | -2.82 | 80.7 | 569.21 | 0.0033 | 0.0418 |
| CTSK | -7.07 | -2.82 | 877.12 | 6197.4 | 0.00359 | 0.0418 |
| LPAR4 | -7.07 | -2.82 | 1.24 | 8.76 | 0.00443 | 0.0418 |
| ZNF471 | -7.08 | -2.82 | 26.94 | 190.8 | 0.00688 | 0.0433 |
| TMEM158 | -7.06 | -2.82 | 196.61 | 1389.01 | 0.00955 | 0.0482 |
| AKT3 | -7.01 | -2.81 | 234.93 | 1646.68 | 0.0033 | 0.0418 |
| CA11 | -7.02 | -2.81 | 253.26 | 1778.45 | 0.00383 | 0.0418 |
| CORO2B | -7 | -2.81 | 38.9 | 272.36 | 0.0052 | 0.0418 |
| THBS4 | -7 | -2.81 | 56.55 | 396.11 | 0.0092 | 0.0476 |
| SH3BP5 | -6.96 | -2.8 | 642.75 | 4471.31 | 0.00317 | 0.0418 |
| DCLK2 | -6.95 | -2.8 | 42.25 | 293.66 | 0.00352 | 0.0418 |
| ANGPTL2 | -6.97 | -2.8 | 506.68 | 3532.26 | 0.00375 | 0.0418 |
| FAM171B | -6.97 | -2.8 | 77.49 | 539.92 | 0.00383 | 0.0418 |
| GSPT2 | -6.97 | -2.8 | 52.89 | 368.72 | 0.00433 | 0.0418 |
| GIMAP6 | -6.96 | -2.8 | 192.05 | 1336.71 | 0.00451 | 0.0418 |
| SHE | -6.97 | -2.8 | 92 | 641.62 | 0.00599 | 0.0421 |
| COL6A3 | -6.96 | -2.8 | 4490.05 | 31265.45 | 0.00688 | 0.0433 |
| ZDHHC15 | -6.99 | -2.8 | 18.17 | 126.97 | 0.007 | 0.0434 |
| FRY | -6.92 | -2.79 | 154.02 | 1066.16 | 0.00304 | 0.0418 |
| KCTD12 | -6.92 | -2.79 | 987.71 | 6838.95 | 0.0031 | 0.0418 |
| GYPE | -6.9 | -2.79 | 2.04 | 14.06 | 0.00334 | 0.0418 |
| SLC1A7 | -6.92 | -2.79 | 15.25 | 105.59 | 0.00449 | 0.0418 |
| XPNPEP2 | -6.89 | -2.79 | 9.01 | 62.15 | 0.00677 | 0.0433 |
| GNAZ | -6.86 | -2.78 | 108.85 | 747.05 | 0.00359 | 0.0418 |
| CLEC1A | -6.88 | -2.78 | 39.87 | 274.16 | 0.00383 | 0.0418 |
| TWIST2 | -6.87 | -2.78 | 42.44 | 291.42 | 0.00399 | 0.0418 |
| LUM | -6.86 | -2.78 | 4226.47 | 28972.97 | 0.00433 | 0.0418 |
| RARRES2 | -6.88 | -2.78 | 999.23 | 6873.97 | 0.005 | 0.0418 |
| LIN7A | -6.89 | -2.78 | 8.23 | 56.72 | 0.00505 | 0.0418 |
| PCOLCE | -6.85 | -2.78 | 749.16 | 5128.5 | 0.0092 | 0.0476 |
| CD200 | -6.84 | -2.77 | 119.66 | 817.96 | 0.00451 | 0.0418 |
| TPSAB1 | -6.82 | -2.77 | 304.42 | 2076.45 | 0.0052 | 0.0418 |
| ACCN1 | -6.81 | -2.77 | 7.93 | 54.02 | 0.00592 | 0.0421 |
| NR2F2 | -6.77 | -2.76 | 815.59 | 5519.75 | 0.00337 | 0.0418 |
| CYR61 | -6.75 | -2.76 | 2418.75 | 16330.12 | 0.00391 | 0.0418 |
| RASSF8 | -6.79 | -2.76 | 206.63 | 1403.38 | 0.00442 | 0.0418 |
| KLHL13 | -6.75 | -2.76 | 207.11 | 1398.27 | 0.00611 | 0.0424 |
| RBPMS | -6.75 | -2.75 | 660.24 | 4454.17 | 0.0031 | 0.0418 |
| C3orf70 | -6.71 | -2.75 | 115.02 | 771.39 | 0.00399 | 0.0418 |
| SLC43A1 | -6.71 | -2.75 | 84.68 | 568.62 | 0.00424 | 0.0418 |
| EXOC3L2 | -6.74 | -2.75 | 40.06 | 270.19 | 0.00587 | 0.0421 |
| HRASLS5 | -6.74 | -2.75 | 11.99 | 80.83 | 0.00679 | 0.0433 |
| DACT1 | -6.71 | -2.75 | 89.2 | 598.75 | 0.00701 | 0.0434 |
| CLMP | -6.71 | -2.75 | 89.99 | 603.46 | 0.00773 | 0.0449 |
| TACC1 | -6.69 | -2.74 | 1169.89 | 7823.83 | 0.00291 | 0.0418 |
| THRA | -6.69 | -2.74 | 420.52 | 2812.78 | 0.00317 | 0.0418 |
| C1orf133 | -6.7 | -2.74 | 47.32 | 316.89 | 0.00399 | 0.0418 |
| SDC2 | -6.67 | -2.74 | 605.39 | 4035.84 | 0.00461 | 0.0418 |
| ILK | -6.62 | -2.73 | 1960.29 | 12969.26 | 0.00291 | 0.0418 |
| GSN | -6.62 | -2.73 | 10109.39 | 66966.5 | 0.00297 | 0.0418 |
| RGS9 | -6.61 | -2.73 | 23.6 | 156.11 | 0.00442 | 0.0418 |
| ZNF880 | -6.61 | -2.73 | 26.01 | 172.08 | 0.00461 | 0.0418 |
| ADAMTS9 | -6.62 | -2.73 | 163.97 | 1085.59 | 0.0049 | 0.0418 |
| RNF150 | -6.64 | -2.73 | 29.87 | 198.21 | 0.00575 | 0.0421 |
| ZNF610 | -6.62 | -2.73 | 15.21 | 100.65 | 0.00789 | 0.0452 |
| TNFSF12 | -6.57 | -2.72 | 273.66 | 1798.04 | 0.00291 | 0.0418 |
| PRX | -6.6 | -2.72 | 101.64 | 670.84 | 0.0031 | 0.0418 |
| FLNA | -6.58 | -2.72 | 21849.85 | 143719.14 | 0.00352 | 0.0418 |
| PTPRB | -6.6 | -2.72 | 266.82 | 1761.57 | 0.00359 | 0.0418 |
| ANO2 | -6.59 | -2.72 | 11.03 | 72.66 | 0.00424 | 0.0418 |
| MSX1 | -6.59 | -2.72 | 207.11 | 1365.7 | 0.00575 | 0.0421 |
| CD248 | -6.54 | -2.71 | 440.68 | 2880.95 | 0.00359 | 0.0418 |
| NHLRC4 | -6.54 | -2.71 | 10.39 | 67.95 | 0.00433 | 0.0418 |
| CYP21A2 | -6.56 | -2.71 | 26 | 170.52 | 0.00461 | 0.0418 |
| VIM | -6.49 | -2.7 | 8890.6 | 57659.08 | 0.00352 | 0.0418 |
| MPDZ | -6.5 | -2.7 | 175.06 | 1137.14 | 0.00391 | 0.0418 |
| AVPR2 | -6.49 | -2.7 | 22.42 | 145.52 | 0.00451 | 0.0418 |
| SALL1 | -6.49 | -2.7 | 90.42 | 587.19 | 0.00623 | 0.0425 |
| C2orf58 | -6.51 | -2.7 | 3.49 | 22.74 | 0.00667 | 0.0432 |
| PDGFRL | -6.48 | -2.7 | 106.94 | 693.38 | 0.0073 | 0.0439 |
| CD302 | -6.47 | -2.69 | 242.13 | 1565.42 | 0.00291 | 0.0418 |
| POU6F1 | -6.44 | -2.69 | 95.53 | 614.93 | 0.00291 | 0.0418 |
| THPO | -6.45 | -2.69 | 6.22 | 40.15 | 0.00406 | 0.0418 |
| FOXF1 | -6.45 | -2.69 | 64.93 | 419 | 0.0051 | 0.0418 |
| MYOM1 | -6.45 | -2.69 | 39.06 | 252.14 | 0.00531 | 0.0418 |
| RELN | -6.48 | -2.69 | 19 | 123.04 | 0.00815 | 0.0456 |
| ANKRD53 | -6.39 | -2.68 | 6.7 | 42.82 | 0.00352 | 0.0418 |
| HTR2A | -6.41 | -2.68 | 1.2 | 7.71 | 0.00441 | 0.0418 |
| C21orf34 | -6.39 | -2.68 | 27.45 | 175.51 | 0.0046 | 0.0418 |
| SOX10 | -6.41 | -2.68 | 5.32 | 34.1 | 0.00481 | 0.0418 |
| DOK6 | -6.4 | -2.68 | 9.57 | 61.21 | 0.00611 | 0.0424 |
| DCHS2 | -6.42 | -2.68 | 17.8 | 114.2 | 0.00686 | 0.0433 |
| LIX1L | -6.36 | -2.67 | 327.02 | 2080.64 | 0.00291 | 0.0418 |
| CAVIN1 | -6.36 | -2.67 | 5097.32 | 32436.32 | 0.0031 | 0.0418 |
| RFTN2 | -6.34 | -2.66 | 23.07 | 146.21 | 0.00291 | 0.0418 |
| DKFZp779M0652 | -6.34 | -2.66 | 1.79 | 11.34 | 0.00418 | 0.0418 |
| ACTN2 | -6.31 | -2.66 | 3.82 | 24.13 | 0.00484 | 0.0418 |
| ADCY2 | -6.33 | -2.66 | 39.83 | 252.09 | 0.00529 | 0.0418 |
| SORCS1 | -6.34 | -2.66 | 8.73 | 55.33 | 0.00597 | 0.0421 |
| PCDHB6 | -6.3 | -2.66 | 11.89 | 74.99 | 0.00599 | 0.0421 |
| PDE8B | -6.33 | -2.66 | 72.3 | 457.75 | 0.00636 | 0.0427 |
| RGS5 | -6.26 | -2.65 | 832.86 | 5217 | 0.00375 | 0.0418 |
| ROR2 | -6.29 | -2.65 | 262.25 | 1650.22 | 0.00407 | 0.0418 |
| SPRY1 | -6.29 | -2.65 | 368.49 | 2318.65 | 0.00416 | 0.0418 |
| PPP1R1A | -6.27 | -2.65 | 12.51 | 78.46 | 0.00907 | 0.0476 |
| ANXA6 | -6.23 | -2.64 | 1283.66 | 7991.61 | 0.00323 | 0.0418 |
| ICAM2 | -6.23 | -2.64 | 208.02 | 1296.29 | 0.00359 | 0.0418 |
| GPR17 | -6.22 | -2.64 | 2.96 | 18.42 | 0.00446 | 0.0418 |
| FGFR1 | -6.23 | -2.64 | 865.04 | 5384.91 | 0.0047 | 0.0418 |
| LOC389705 | -6.22 | -2.64 | 3.3 | 20.52 | 0.0052 | 0.0418 |
| PIP4P2 | -6.21 | -2.63 | 76.89 | 477.22 | 0.00317 | 0.0418 |
| MAGEH1 | -6.19 | -2.63 | 177.23 | 1096.95 | 0.0033 | 0.0418 |
| CNTNAP1 | -6.21 | -2.63 | 153.97 | 956.19 | 0.00367 | 0.0418 |
| BCL2 | -6.17 | -2.63 | 313.22 | 1933.45 | 0.00442 | 0.0418 |
| ADAMTS3 | -6.2 | -2.63 | 18.45 | 114.46 | 0.00992 | 0.0488 |
| ARID5A | -6.15 | -2.62 | 524.4 | 3226.03 | 0.0033 | 0.0418 |
| CCDC81 | -6.13 | -2.62 | 10.16 | 62.27 | 0.00337 | 0.0418 |
| CAB39L | -6.15 | -2.62 | 172.72 | 1061.85 | 0.00359 | 0.0418 |
| TUBA1A | -6.15 | -2.62 | 2478.31 | 15231.02 | 0.00367 | 0.0418 |
| ANGPT1 | -6.15 | -2.62 | 32.59 | 200.51 | 0.0047 | 0.0418 |
| MOXD1 | -6.16 | -2.62 | 366.93 | 2260.79 | 0.0049 | 0.0418 |
| EFHD1 | -6.16 | -2.62 | 309.6 | 1906.9 | 0.00587 | 0.0421 |
| STK32B | -6.17 | -2.62 | 30.26 | 186.55 | 0.00623 | 0.0425 |
| WFS1 | -6.1 | -2.61 | 716.52 | 4368.58 | 0.00317 | 0.0418 |
| GJA5 | -6.09 | -2.61 | 76.36 | 464.68 | 0.0051 | 0.0418 |
| ZFP28 | -6.11 | -2.61 | 48.47 | 296.18 | 0.0051 | 0.0418 |
| EFCAB1 | -6.09 | -2.61 | 35.24 | 214.68 | 0.00661 | 0.043 |
| PALM2-AKAP2 | -6.05 | -2.6 | 471.49 | 2854 | 0.00323 | 0.0418 |
| S1PR3 | -6.05 | -2.6 | 238.42 | 1441.65 | 0.00383 | 0.0418 |
| PLPP1 | -6.06 | -2.6 | 575.81 | 3491.6 | 0.00399 | 0.0418 |
| GIMAP7 | -6.05 | -2.6 | 146.05 | 883.67 | 0.0051 | 0.0418 |
| GLIPR1L2 | -6.04 | -2.59 | 7.74 | 46.75 | 0.00345 | 0.0418 |
| ZNF826 | -6 | -2.59 | 8.99 | 53.93 | 0.00552 | 0.0418 |
| ASTN1 | -6 | -2.59 | 7.93 | 47.58 | 0.00692 | 0.0434 |
| GRIA3 | -6.01 | -2.59 | 6.38 | 38.34 | 0.00758 | 0.0445 |
| CTSF | -5.98 | -2.58 | 877.05 | 5244.89 | 0.00337 | 0.0418 |
| DLG4 | -5.99 | -2.58 | 132.62 | 794.45 | 0.00337 | 0.0418 |
| SLC22A17 | -5.96 | -2.58 | 217.17 | 1294.27 | 0.00399 | 0.0418 |
| **Gene** | **Fold change** | **log2 (Fold change)** | **Mean TPM* (tumor)** | **Mean TPM* (normal)** | **p-value** | **adjusted p-value** |
| SAMD4A | -6 | -2.58 | 257.67 | 1545.44 | 0.00424 | 0.0418 |
| PTPRM | -5.94 | -2.57 | 325.41 | 1932.88 | 0.00531 | 0.0418 |
| C14orf132 | -5.96 | -2.57 | 288.97 | 1721.12 | 0.00599 | 0.0421 |
| MAGI2 | -5.89 | -2.56 | 60.35 | 355.72 | 0.00304 | 0.0418 |
| GRIK1 | -5.88 | -2.56 | 1.8 | 10.6 | 0.00395 | 0.0418 |
| HIF3A | -5.89 | -2.56 | 83.84 | 493.61 | 0.005 | 0.0418 |
| KCNIP1 | -5.9 | -2.56 | 7.46 | 44.02 | 0.00743 | 0.0442 |
| BEX5 | -5.91 | -2.56 | 30.49 | 180.17 | 0.00868 | 0.0467 |
| TTC28 | -5.86 | -2.55 | 249.12 | 1460.38 | 0.0033 | 0.0418 |
| TSHZ3 | -5.84 | -2.55 | 199.93 | 1168.5 | 0.00352 | 0.0418 |
| CAP2 | -5.85 | -2.55 | 153.33 | 896.64 | 0.00359 | 0.0418 |
| P3H3 | -5.85 | -2.55 | 429.77 | 2513.78 | 0.00359 | 0.0418 |
| PCDH11X | -5.85 | -2.55 | 1.94 | 11.34 | 0.00401 | 0.0418 |
| DPYSL3 | -5.86 | -2.55 | 1746.87 | 10238.25 | 0.0052 | 0.0418 |
| RAPGEF4 | -5.84 | -2.55 | 53.81 | 314.1 | 0.0073 | 0.0439 |
| STARD13 | -5.8 | -2.54 | 164.29 | 952.71 | 0.00297 | 0.0418 |
| MCAM | -5.81 | -2.54 | 1403.4 | 8160.07 | 0.00407 | 0.0418 |
| CYP1B1 | -5.82 | -2.54 | 308.98 | 1799.17 | 0.00442 | 0.0418 |
| GJC2 | -5.83 | -2.54 | 56.38 | 328.6 | 0.00442 | 0.0418 |
| PALLD | -5.77 | -2.53 | 3408.86 | 19656.4 | 0.00291 | 0.0418 |
| ZDHHC14 | -5.78 | -2.53 | 178.39 | 1031.56 | 0.00297 | 0.0418 |
| TBX2 | -5.76 | -2.53 | 142.52 | 821.6 | 0.00433 | 0.0418 |
| CKMT2 | -5.77 | -2.53 | 13.29 | 76.71 | 0.00468 | 0.0418 |
| REV3L | -5.79 | -2.53 | 470.73 | 2724.68 | 0.0047 | 0.0418 |
| PRRT2 | -5.76 | -2.53 | 46.41 | 267.47 | 0.0051 | 0.0418 |
| NCRNA00087 | -5.79 | -2.53 | 26.79 | 155.09 | 0.0052 | 0.0418 |
| NME5 | -5.78 | -2.53 | 14.28 | 82.5 | 0.00819 | 0.0456 |
| IGFBP4 | -5.73 | -2.52 | 7613.8 | 43651.45 | 0.0033 | 0.0418 |
| TBXA2R | -5.74 | -2.52 | 30.39 | 174.58 | 0.00359 | 0.0418 |
| IGSF9B | -5.75 | -2.52 | 8.17 | 46.95 | 0.00753 | 0.0445 |
| KNDC1 | -5.72 | -2.52 | 43.91 | 251.01 | 0.00804 | 0.0454 |
| RASL11A | -5.7 | -2.51 | 125.91 | 717.48 | 0.0048 | 0.0418 |
| IGF2 | -5.68 | -2.51 | 1311.15 | 7448.98 | 0.005 | 0.0418 |
| CALHM5 | -5.68 | -2.51 | 28 | 159.05 | 0.0051 | 0.0418 |
| REEP1 | -5.71 | -2.51 | 115.58 | 660.23 | 0.00542 | 0.0418 |
| BHMT2 | -5.71 | -2.51 | 37.66 | 214.84 | 0.00599 | 0.0421 |
| MFGE8 | -5.65 | -2.5 | 1614.58 | 9129.97 | 0.0031 | 0.0418 |
| ADGRE5 | -5.66 | -2.5 | 1428.34 | 8087.62 | 0.0033 | 0.0418 |
| CEP112 | -5.64 | -2.5 | 71.72 | 404.77 | 0.00337 | 0.0418 |
| BST1 | -5.67 | -2.5 | 31.07 | 176.17 | 0.00367 | 0.0418 |
| NR5A2 | -5.67 | -2.5 | 60.23 | 341.26 | 0.00367 | 0.0418 |
| PCDHB12 | -5.64 | -2.5 | 19.74 | 111.37 | 0.00461 | 0.0418 |
| SCUBE2 | -5.64 | -2.5 | 142.11 | 802.05 | 0.0048 | 0.0418 |
| SH3BGRL | -5.61 | -2.49 | 1157.4 | 6493.38 | 0.00291 | 0.0418 |
| FOXO1 | -5.6 | -2.49 | 613.75 | 3437.83 | 0.00297 | 0.0418 |
| PDGFRB | -5.6 | -2.49 | 1249.94 | 7000.86 | 0.00337 | 0.0418 |
| GAS7 | -5.62 | -2.49 | 323.21 | 1816.29 | 0.00424 | 0.0418 |
| CA3 | -5.61 | -2.49 | 18.8 | 105.53 | 0.0052 | 0.0418 |
| GPR162 | -5.6 | -2.49 | 26.26 | 147.12 | 0.0052 | 0.0418 |
| SYNM | -5.63 | -2.49 | 475.55 | 2675.45 | 0.00636 | 0.0427 |
| IGFBP6 | -5.61 | -2.49 | 653.78 | 3665.57 | 0.00773 | 0.0449 |
| ERG | -5.58 | -2.48 | 208.11 | 1161.13 | 0.0033 | 0.0418 |
| HNMT | -5.59 | -2.48 | 258.84 | 1446.23 | 0.00359 | 0.0418 |
| HOXA11AS | -5.59 | -2.48 | 71.62 | 400.68 | 0.00367 | 0.0418 |
| CDH5 | -5.57 | -2.48 | 310.86 | 1731.83 | 0.00424 | 0.0418 |
| FBN1 | -5.58 | -2.48 | 750.67 | 4185.33 | 0.00789 | 0.0452 |
| MYOT | -5.59 | -2.48 | 4.05 | 22.66 | 0.00798 | 0.0454 |
| CD81 | -5.54 | -2.47 | 6692.25 | 37092.99 | 0.00291 | 0.0418 |
| VAMP2 | -5.52 | -2.47 | 954.62 | 5271.99 | 0.00291 | 0.0418 |
| KIF17 | -5.55 | -2.47 | 24.88 | 138.05 | 0.00345 | 0.0418 |
| LOC257358 | -5.56 | -2.47 | 1.43 | 7.95 | 0.00427 | 0.0418 |
| GALNT16 | -5.55 | -2.47 | 67.63 | 375.19 | 0.00992 | 0.0488 |
| TGFBR2 | -5.49 | -2.46 | 1125.62 | 6181.51 | 0.00317 | 0.0418 |
| ITPKB | -5.51 | -2.46 | 855.11 | 4713.04 | 0.0033 | 0.0418 |
| CAV1 | -5.48 | -2.46 | 2589.4 | 14199.36 | 0.00461 | 0.0418 |
| EFHB | -5.5 | -2.46 | 3.55 | 19.54 | 0.00484 | 0.0418 |
| MYOZ1 | -5.49 | -2.46 | 7.41 | 40.73 | 0.00648 | 0.043 |
| ACACB | -5.46 | -2.45 | 262.55 | 1432.49 | 0.00317 | 0.0418 |
| C14orf28 | -5.46 | -2.45 | 76.88 | 420.07 | 0.00317 | 0.0418 |
| GATA2 | -5.45 | -2.45 | 352.8 | 1921.7 | 0.00391 | 0.0418 |
| PRRX1 | -5.45 | -2.45 | 790.41 | 4306.07 | 0.00424 | 0.0418 |
| CRY2 | -5.42 | -2.44 | 460.06 | 2491.94 | 0.00297 | 0.0418 |
| GAB2 | -5.41 | -2.44 | 299.37 | 1620.21 | 0.00352 | 0.0418 |
| FLJ42709 | -5.44 | -2.44 | 56.58 | 307.85 | 0.00407 | 0.0418 |
| ZNF583 | -5.44 | -2.44 | 17.24 | 93.84 | 0.00688 | 0.0433 |
| MAB21L1 | -5.41 | -2.44 | 1.53 | 8.3 | 0.00775 | 0.045 |
| LOC100128640 | -5.42 | -2.44 | 16.14 | 87.55 | 0.00868 | 0.0467 |
| LOC286367 | -5.39 | -2.43 | 26.69 | 143.94 | 0.0031 | 0.0418 |
| GULP1 | -5.39 | -2.43 | 165.38 | 890.68 | 0.00399 | 0.0418 |
| COL6A1 | -5.37 | -2.43 | 4850.14 | 26065.17 | 0.0048 | 0.0418 |
| C6orf217 | -5.38 | -2.43 | 1.36 | 7.33 | 0.00597 | 0.0421 |
| GLI1 | -5.38 | -2.43 | 25.35 | 136.38 | 0.00611 | 0.0424 |
| SLITRK2 | -5.39 | -2.43 | 7.58 | 40.84 | 0.00764 | 0.0448 |
| SFRP1 | -5.39 | -2.43 | 1445.09 | 7784.46 | 0.00773 | 0.0449 |
| CPA3 | -5.37 | -2.43 | 127.42 | 684.88 | 0.00789 | 0.0452 |
| ART4 | -5.37 | -2.42 | 2.31 | 12.42 | 0.00296 | 0.0418 |
| GIMAP5 | -5.36 | -2.42 | 259.42 | 1391.63 | 0.00383 | 0.0418 |
| SLC6A1 | -5.33 | -2.42 | 7.16 | 38.19 | 0.00407 | 0.0418 |
| RAB11FIP2 | -5.33 | -2.41 | 223.96 | 1192.78 | 0.00304 | 0.0418 |
| SPTBN4 | -5.32 | -2.41 | 26.21 | 139.49 | 0.00564 | 0.0419 |
| IGF2-AS | -5.33 | -2.41 | 3.63 | 19.33 | 0.00605 | 0.0424 |
| NRK | -5.31 | -2.41 | 24.1 | 127.94 | 0.00685 | 0.0433 |
| SPEF2 | -5.3 | -2.41 | 65.9 | 349.12 | 0.00715 | 0.0437 |
| HABP4 | -5.29 | -2.4 | 167.34 | 884.88 | 0.00352 | 0.0418 |
| CCDC48 | -5.28 | -2.4 | 34.97 | 184.81 | 0.00531 | 0.0418 |
| IGFN1 | -5.29 | -2.4 | 9.79 | 51.77 | 0.00611 | 0.0424 |
| AXIN2 | -5.25 | -2.39 | 255.45 | 1340.7 | 0.00587 | 0.0421 |
| KIF19 | -5.24 | -2.39 | 7.72 | 40.46 | 0.00622 | 0.0425 |
| NCRNA00219 | -5.22 | -2.39 | 246.66 | 1288.78 | 0.00744 | 0.0442 |
| ZNF25 | -5.21 | -2.38 | 132.19 | 688.51 | 0.00291 | 0.0418 |
| FAM66C | -5.2 | -2.38 | 9.95 | 51.79 | 0.00391 | 0.0418 |
| ZCCHC18 | -5.19 | -2.38 | 9.82 | 51.01 | 0.00424 | 0.0418 |
| COX4I2 | -5.2 | -2.38 | 19.73 | 102.53 | 0.00451 | 0.0418 |
| PBX1 | -5.22 | -2.38 | 1869.04 | 9754.28 | 0.00461 | 0.0418 |
| CDKL1 | -5.21 | -2.38 | 19.62 | 102.26 | 0.0047 | 0.0418 |
| PDLIM7 | -5.18 | -2.37 | 1726.62 | 8950.48 | 0.00352 | 0.0418 |
| EML1 | -5.18 | -2.37 | 155.52 | 804.86 | 0.0052 | 0.0418 |
| RGS13 | -5.18 | -2.37 | 4.98 | 25.81 | 0.00674 | 0.0432 |
| LMCD1 | -5.12 | -2.36 | 413.42 | 2118.45 | 0.0033 | 0.0418 |
| CDH19 | -5.12 | -2.36 | 2.29 | 11.74 | 0.00345 | 0.0418 |
| FAP | -5.13 | -2.36 | 305.37 | 1566.83 | 0.0049 | 0.0418 |
| GIMAP1 | -5.13 | -2.36 | 79.05 | 405.21 | 0.0049 | 0.0418 |
| ZIK1 | -5.14 | -2.36 | 13.77 | 70.86 | 0.00599 | 0.0421 |
| SLC27A6 | -5.14 | -2.36 | 8.35 | 42.96 | 0.00914 | 0.0476 |
| SAMD5 | -5.15 | -2.36 | 63.79 | 328.44 | 0.0092 | 0.0476 |
| C11orf95 | -5.1 | -2.35 | 314.63 | 1604.26 | 0.00323 | 0.0418 |
| PRICKLE1 | -5.11 | -2.35 | 121.49 | 621.28 | 0.00391 | 0.0418 |
| SNPH | -5.11 | -2.35 | 69.88 | 357.37 | 0.00451 | 0.0418 |
| CTXN1 | -5.1 | -2.35 | 203.75 | 1039.23 | 0.0047 | 0.0418 |
| MEG3 | -5.09 | -2.35 | 69.3 | 352.8 | 0.0047 | 0.0418 |
| AEBP1 | -5.1 | -2.35 | 3989.11 | 20344.36 | 0.0048 | 0.0418 |
| PLVAP | -5.08 | -2.35 | 949.16 | 4823.73 | 0.005 | 0.0418 |
| SCARF1 | -5.05 | -2.34 | 111.01 | 560.38 | 0.00304 | 0.0418 |
| MAP3K20 | -5.06 | -2.34 | 1200.25 | 6074.2 | 0.0031 | 0.0418 |
| CCDC69 | -5.07 | -2.34 | 416.99 | 2113.97 | 0.0033 | 0.0418 |
| LRRK2 | -5.07 | -2.34 | 101.2 | 512.75 | 0.0051 | 0.0418 |
| KIAA1683 | -5.07 | -2.34 | 81.72 | 413.95 | 0.00564 | 0.0419 |
| TMEM108 | -5.07 | -2.34 | 12.05 | 61.17 | 0.00622 | 0.0425 |
| NRXN1 | -5.07 | -2.34 | 11.67 | 59.17 | 0.00755 | 0.0445 |
| PARVA | -5.02 | -2.33 | 927.69 | 4660.25 | 0.00317 | 0.0418 |
| KLHDC1 | -5.04 | -2.33 | 27.01 | 136.02 | 0.00611 | 0.0424 |
| ADGRL3 | -5.03 | -2.33 | 51.4 | 258.45 | 0.00623 | 0.0425 |
| MMP2 | -5.03 | -2.33 | 3842.02 | 19324.13 | 0.00661 | 0.043 |
| TXNIP | -5.01 | -2.32 | 8294.19 | 41512.67 | 0.00337 | 0.0418 |
| IGDCC4 | -5 | -2.32 | 32.11 | 160.37 | 0.00424 | 0.0418 |
| LOC158376 | -5.01 | -2.32 | 5.61 | 28.1 | 0.0047 | 0.0418 |
| RASGRF2 | -5.01 | -2.32 | 27.61 | 138.29 | 0.0047 | 0.0418 |
| IGFBP7 | -4.99 | -2.32 | 3491.97 | 17424.01 | 0.00587 | 0.0421 |
| ZBTB47 | -4.97 | -2.31 | 325.59 | 1618.75 | 0.00304 | 0.0418 |
| LOH12CR2 | -4.95 | -2.31 | 10.83 | 53.59 | 0.00407 | 0.0418 |
| PARM1 | -4.97 | -2.31 | 481.84 | 2397.14 | 0.0049 | 0.0418 |
| PCBP3 | -4.95 | -2.31 | 25.41 | 125.89 | 0.00599 | 0.0421 |
| DNAJC18 | -4.91 | -2.3 | 78.68 | 386.5 | 0.00291 | 0.0418 |
| LAMB2 | -4.92 | -2.3 | 2127.22 | 10466.08 | 0.0031 | 0.0418 |
| HSD17B11 | -4.94 | -2.3 | 481.75 | 2379.47 | 0.00352 | 0.0418 |
| FZD4 | -4.91 | -2.3 | 365.02 | 1792.5 | 0.0048 | 0.0418 |
| SHF | -4.93 | -2.3 | 96.15 | 474.46 | 0.00553 | 0.0418 |
| PAR5 | -4.91 | -2.3 | 4.58 | 22.5 | 0.00564 | 0.0419 |
| FLT1 | -4.89 | -2.29 | 497.68 | 2432.03 | 0.00391 | 0.0418 |
| BEX4 | -4.88 | -2.29 | 420.01 | 2050.34 | 0.00599 | 0.0421 |
| EPS8 | -4.88 | -2.29 | 710.23 | 3466.88 | 0.00636 | 0.0427 |
| FOXS1 | -4.88 | -2.29 | 29.39 | 143.4 | 0.00636 | 0.0427 |
| ATP2B2 | -4.89 | -2.29 | 12.69 | 62.05 | 0.00743 | 0.0442 |
| TMEM130 | -4.88 | -2.29 | 35.59 | 173.6 | 0.0082 | 0.0456 |
| BEST1 | -4.84 | -2.28 | 19.95 | 96.64 | 0.00323 | 0.0418 |
| LGALS1 | -4.85 | -2.28 | 4820.2 | 23399.53 | 0.00433 | 0.0418 |
| SCARF2 | -4.87 | -2.28 | 142.97 | 696.75 | 0.00575 | 0.0421 |
| SLC4A3 | -4.84 | -2.28 | 130.74 | 633.33 | 0.00623 | 0.0425 |
| SULT1C4 | -4.85 | -2.28 | 13.16 | 63.89 | 0.00758 | 0.0445 |
| MAMSTR | -4.87 | -2.28 | 59.49 | 289.9 | 0.00885 | 0.0471 |
| MFRP | -4.83 | -2.27 | 271.03 | 1310.03 | 0.00391 | 0.0418 |
| FYN | -4.83 | -2.27 | 452.33 | 2185.71 | 0.0049 | 0.0418 |
| TNNI3K | -4.84 | -2.27 | 6.59 | 31.91 | 0.0055 | 0.0418 |
| PCDHGA11 | -4.83 | -2.27 | 6.89 | 33.3 | 0.00622 | 0.0425 |
| IPW | -4.84 | -2.27 | 146.09 | 706.5 | 0.00701 | 0.0434 |
| DMGDH | -4.84 | -2.27 | 11.43 | 55.32 | 0.00819 | 0.0456 |
| VGLL3 | -4.84 | -2.27 | 162.28 | 784.63 | 0.00852 | 0.0464 |
| LOC284440 | -4.78 | -2.26 | 38.45 | 183.7 | 0.0031 | 0.0418 |
| GPR4 | -4.8 | -2.26 | 75.4 | 361.85 | 0.00337 | 0.0418 |
| IFFO1 | -4.78 | -2.26 | 124.95 | 596.9 | 0.00352 | 0.0418 |
| GUCY1A3 | -4.8 | -2.26 | 208.34 | 999.13 | 0.00407 | 0.0418 |
| ARMC9 | -4.79 | -2.26 | 140.87 | 674.97 | 0.0048 | 0.0418 |
| ATP2B4 | -4.78 | -2.26 | 3517.74 | 16801.85 | 0.0052 | 0.0418 |
| BMPER | -4.78 | -2.26 | 27.56 | 131.74 | 0.00647 | 0.043 |
| SOBP | -4.77 | -2.26 | 93.91 | 448.43 | 0.0073 | 0.0439 |
| **Gene** | **Fold change** | **log2 (Fold change)** | **Mean TPM* (tumor)** | **Mean TPM* (normal)** | **p-value** | **adjusted p-value** |
| CAPN6 | -4.79 | -2.26 | 132.13 | 633.31 | 0.00863 | 0.0467 |
| ABCD2 | -4.79 | -2.26 | 12.23 | 58.59 | 0.00902 | 0.0474 |
| SH2D3C | -4.75 | -2.25 | 193.95 | 921.55 | 0.0031 | 0.0418 |
| FAM229B | -4.75 | -2.25 | 110.37 | 524.11 | 0.00359 | 0.0418 |
| GJC1 | -4.75 | -2.25 | 283.41 | 1346.1 | 0.00442 | 0.0418 |
| C14orf37 | -4.74 | -2.25 | 32.11 | 152.34 | 0.00451 | 0.0418 |
| NPDC1 | -4.75 | -2.25 | 851.03 | 4038.15 | 0.0047 | 0.0418 |
| S1PR2 | -4.74 | -2.25 | 219.02 | 1039.22 | 0.00674 | 0.0432 |
| LARGE1 | -4.74 | -2.24 | 279.53 | 1323.57 | 0.00424 | 0.0418 |
| ADAMTS10 | -4.71 | -2.24 | 65.79 | 310.1 | 0.0049 | 0.0418 |
| CACNA1G | -4.71 | -2.24 | 25.81 | 121.65 | 0.0049 | 0.0418 |
| FAM69B | -4.73 | -2.24 | 119.19 | 563.86 | 0.00688 | 0.0433 |
| PTN | -4.72 | -2.24 | 549.49 | 2595.78 | 0.00885 | 0.0471 |
| PM20D1 | -4.72 | -2.24 | 1.23 | 5.81 | 0.0098 | 0.0488 |
| CTF1 | -4.69 | -2.23 | 142.04 | 666.05 | 0.00337 | 0.0418 |
| MOCS1 | -4.69 | -2.23 | 175.92 | 825.52 | 0.00461 | 0.0418 |
| SYNDIG1L | -4.68 | -2.23 | 2.46 | 11.49 | 0.0049 | 0.0418 |
| ELFN1 | -4.71 | -2.23 | 32.84 | 154.57 | 0.00587 | 0.0421 |
| WDR86 | -4.69 | -2.23 | 29.81 | 139.81 | 0.00623 | 0.0425 |
| MAP3K12 | -4.66 | -2.22 | 102.09 | 476.26 | 0.00391 | 0.0418 |
| EPHA3 | -4.65 | -2.22 | 74.77 | 347.79 | 0.00542 | 0.0418 |
| SYN3 | -4.66 | -2.22 | 2.89 | 13.47 | 0.00586 | 0.0421 |
| TSPYL2 | -4.63 | -2.21 | 572.44 | 2649.09 | 0.00297 | 0.0418 |
| TMEM98 | -4.62 | -2.21 | 638.92 | 2954.26 | 0.00383 | 0.0418 |
| ZNF568 | -4.62 | -2.21 | 35.73 | 164.95 | 0.00451 | 0.0418 |
| CCDC141 | -4.63 | -2.21 | 4.35 | 20.12 | 0.00489 | 0.0418 |
| SCN1B | -4.61 | -2.21 | 69.82 | 321.94 | 0.00542 | 0.0418 |
| LDOC1 | -4.64 | -2.21 | 253.99 | 1177.53 | 0.00587 | 0.0421 |
| ZNF491 | -4.63 | -2.21 | 8.95 | 41.43 | 0.00701 | 0.0434 |
| INPP5A | -4.58 | -2.2 | 437.24 | 2002.66 | 0.00291 | 0.0418 |
| PDGFC | -4.59 | -2.2 | 387.8 | 1779.97 | 0.00367 | 0.0418 |
| ZEB2 | -4.59 | -2.2 | 241.44 | 1107.04 | 0.00383 | 0.0418 |
| ARHGAP31 | -4.6 | -2.2 | 217.77 | 1001.49 | 0.00461 | 0.0418 |
| HYMAI | -4.6 | -2.2 | 1.6 | 7.37 | 0.00478 | 0.0418 |
| GIPC3 | -4.6 | -2.2 | 33.42 | 153.68 | 0.005 | 0.0418 |
| HAPLN4 | -4.59 | -2.2 | 8.13 | 37.3 | 0.0051 | 0.0418 |
| FAM117A | -4.59 | -2.2 | 197.51 | 905.95 | 0.00587 | 0.0421 |
| ST8SIA1 | -4.58 | -2.2 | 39.06 | 178.98 | 0.00804 | 0.0454 |
| SPARC | -4.6 | -2.2 | 11439.03 | 52660.83 | 0.00974 | 0.0487 |
| SOX18 | -4.58 | -2.19 | 151.13 | 691.84 | 0.00407 | 0.0418 |
| TMEM31 | -4.55 | -2.19 | 1.06 | 4.82 | 0.00526 | 0.0418 |
| CSRNP1 | -4.56 | -2.19 | 728.12 | 3319.72 | 0.00744 | 0.0442 |
| PAM | -4.57 | -2.19 | 2524.55 | 11540.24 | 0.0082 | 0.0456 |
| MTURN | -4.53 | -2.18 | 484.06 | 2193.61 | 0.00367 | 0.0418 |
| RPS6KA2 | -4.54 | -2.18 | 515.54 | 2339.29 | 0.00375 | 0.0418 |
| RHOB | -4.54 | -2.18 | 4281.48 | 19442.76 | 0.00424 | 0.0418 |
| FLI1 | -4.54 | -2.18 | 216.99 | 986.12 | 0.0052 | 0.0418 |
| SYN2 | -4.52 | -2.18 | 26.15 | 118.2 | 0.00529 | 0.0418 |
| EGR1 | -4.53 | -2.18 | 5408.83 | 24494.41 | 0.00648 | 0.043 |
| BMP6 | -4.53 | -2.18 | 70.11 | 317.86 | 0.00688 | 0.0433 |
| SPART | -4.5 | -2.17 | 692.55 | 3114.33 | 0.00291 | 0.0418 |
| FNBP1 | -4.51 | -2.17 | 887.52 | 4005.26 | 0.00304 | 0.0418 |
| ZYX | -4.49 | -2.17 | 4055.5 | 18188.97 | 0.00323 | 0.0418 |
| NRP2 | -4.5 | -2.17 | 827.93 | 3728.31 | 0.00424 | 0.0418 |
| PCDHB16 | -4.49 | -2.17 | 64.53 | 289.95 | 0.00599 | 0.0421 |
| PAR-SN | -4.51 | -2.17 | 29.16 | 131.44 | 0.00648 | 0.043 |
| KIAA1614 | -4.49 | -2.17 | 51.32 | 230.57 | 0.00773 | 0.0449 |
| NBEA | -4.5 | -2.17 | 120.89 | 544.33 | 0.00868 | 0.0467 |
| NIPSNAP3B | -4.46 | -2.16 | 18.73 | 83.54 | 0.00352 | 0.0418 |
| NOTCH4 | -4.48 | -2.16 | 206.91 | 927.23 | 0.00375 | 0.0418 |
| SERPING1 | -4.47 | -2.16 | 4299.89 | 19199.75 | 0.00391 | 0.0418 |
| MAN1C1 | -4.46 | -2.16 | 166.41 | 742.01 | 0.00416 | 0.0418 |
| PRKCA | -4.46 | -2.16 | 447.45 | 1997.44 | 0.00575 | 0.0421 |
| TRO | -4.48 | -2.16 | 90.98 | 407.35 | 0.00636 | 0.0427 |
| ADAMTSL3 | -4.47 | -2.16 | 124.82 | 558.53 | 0.00688 | 0.0433 |
| CPXM1 | -4.46 | -2.16 | 436.69 | 1947.69 | 0.00715 | 0.0437 |
| TRAM1L1 | -4.46 | -2.16 | 12.77 | 56.91 | 0.00773 | 0.0449 |
| IL33 | -4.47 | -2.16 | 506.44 | 2264.73 | 0.00789 | 0.0452 |
| LOC441666 | -4.48 | -2.16 | 10.26 | 46 | 0.00988 | 0.0488 |
| LOH3CR2A | -4.43 | -2.15 | 27.55 | 122.12 | 0.00337 | 0.0418 |
| TSC22D1 | -4.45 | -2.15 | 3868.26 | 17200.21 | 0.00345 | 0.0418 |
| CYP2U1 | -4.45 | -2.15 | 107.94 | 480.4 | 0.00367 | 0.0418 |
| DLL4 | -4.43 | -2.15 | 128.89 | 571.2 | 0.00407 | 0.0418 |
| MEIS3P1 | -4.43 | -2.15 | 132.28 | 586.41 | 0.00451 | 0.0418 |
| BACH2 | -4.44 | -2.15 | 80.03 | 355.1 | 0.00531 | 0.0418 |
| SNCAIP | -4.44 | -2.15 | 218.2 | 968.73 | 0.00542 | 0.0418 |
| CDC42EP3 | -4.45 | -2.15 | 604.64 | 2688.02 | 0.00553 | 0.0418 |
| MROH8 | -4.45 | -2.15 | 12.67 | 56.36 | 0.00623 | 0.0425 |
| FAM124B | -4.45 | -2.15 | 15.01 | 66.86 | 0.00674 | 0.0432 |
| PLXNA4 | -4.45 | -2.15 | 54 | 240.3 | 0.00759 | 0.0445 |
| GLIPR2 | -4.41 | -2.14 | 497.16 | 2192.02 | 0.0051 | 0.0418 |
| SUSD5 | -4.41 | -2.14 | 40.85 | 180.34 | 0.00868 | 0.0467 |
| MMP19 | -4.4 | -2.14 | 367.57 | 1618.44 | 0.0092 | 0.0476 |
| EID2B | -4.37 | -2.13 | 38.35 | 167.71 | 0.00399 | 0.0418 |
| GRAPL | -4.37 | -2.13 | 1.48 | 6.48 | 0.00595 | 0.0421 |
| ZNF876P | -4.37 | -2.13 | 11.03 | 48.17 | 0.00611 | 0.0424 |
| ZNF542 | -4.38 | -2.13 | 91.07 | 399.11 | 0.00836 | 0.046 |
| CLCNKB | -4.39 | -2.13 | 7.66 | 33.59 | 0.00942 | 0.0481 |
| RNF144A | -4.34 | -2.12 | 438.66 | 1903.52 | 0.00352 | 0.0418 |
| HTRA1 | -4.34 | -2.12 | 1926.33 | 8362.79 | 0.00442 | 0.0418 |
| CUBN | -4.34 | -2.12 | 12.24 | 53.19 | 0.00461 | 0.0418 |
| SHANK3 | -4.36 | -2.12 | 288.23 | 1256.48 | 0.00461 | 0.0418 |
| INE2 | -4.35 | -2.12 | 14.23 | 61.82 | 0.00496 | 0.0418 |
| LOC92973 | -4.33 | -2.12 | 15.27 | 66.15 | 0.005 | 0.0418 |
| HSPA12A | -4.34 | -2.12 | 107.75 | 467.86 | 0.0052 | 0.0418 |
| DSEL | -4.35 | -2.12 | 91.92 | 399.68 | 0.00575 | 0.0421 |
| SVIL | -4.34 | -2.12 | 2153.34 | 9341.41 | 0.00701 | 0.0434 |
| RIPOR3 | -4.36 | -2.12 | 93.36 | 406.62 | 0.0073 | 0.0439 |
| SPON2 | -4.35 | -2.12 | 911.75 | 3969.84 | 0.00789 | 0.0452 |
| RUSC2 | -4.32 | -2.11 | 415.81 | 1795.84 | 0.00297 | 0.0418 |
| TMEM8B | -4.33 | -2.11 | 237.83 | 1028.79 | 0.00297 | 0.0418 |
| STAT5B | -4.33 | -2.11 | 1156.87 | 5004.85 | 0.00317 | 0.0418 |
| PCDHGC3 | -4.31 | -2.11 | 1178.49 | 5083.25 | 0.00337 | 0.0418 |
| IL3RA | -4.32 | -2.11 | 95.3 | 411.49 | 0.00367 | 0.0418 |
| LTBP3 | -4.32 | -2.11 | 1510 | 6521.42 | 0.00375 | 0.0418 |
| ADARB1 | -4.3 | -2.11 | 280.53 | 1207.14 | 0.00383 | 0.0418 |
| MPZ | -4.32 | -2.11 | 18.01 | 77.85 | 0.00383 | 0.0418 |
| NAT2 | -4.33 | -2.11 | 3.34 | 14.45 | 0.00438 | 0.0418 |
| ZBTB46 | -4.31 | -2.11 | 90.25 | 389.44 | 0.00587 | 0.0421 |
| CLSTN2 | -4.32 | -2.11 | 42.67 | 184.16 | 0.00715 | 0.0437 |
| UNC5C | -4.33 | -2.11 | 5.58 | 24.14 | 0.00843 | 0.0464 |
| SLCO2A1 | -4.31 | -2.11 | 690.41 | 2974.57 | 0.00902 | 0.0474 |
| SYS1-DBNDD2 | -4.29 | -2.1 | 14.96 | 64.24 | 0.00341 | 0.0418 |
| ZCWPW2 | -4.29 | -2.1 | 5.85 | 25.07 | 0.00359 | 0.0418 |
| EMP3 | -4.29 | -2.1 | 671.05 | 2875.54 | 0.00461 | 0.0418 |
| SOX5 | -4.28 | -2.1 | 10.52 | 45.04 | 0.00786 | 0.0452 |
| NRN1 | -4.29 | -2.1 | 167.42 | 717.55 | 0.00852 | 0.0464 |
| HSPB8 | -4.29 | -2.1 | 862.64 | 3699.85 | 0.00955 | 0.0482 |
| GTF2IRD2B | -4.26 | -2.09 | 72.93 | 310.85 | 0.0033 | 0.0418 |
| DZIP1 | -4.27 | -2.09 | 264.99 | 1131.19 | 0.00407 | 0.0418 |
| DNM3 | -4.27 | -2.09 | 43.8 | 186.96 | 0.00715 | 0.0437 |
| DAB2 | -4.21 | -2.08 | 466.5 | 1965.65 | 0.00636 | 0.0427 |
| PRKD1 | -4.22 | -2.08 | 68.16 | 287.58 | 0.00759 | 0.0445 |
| ZNF415 | -4.24 | -2.08 | 47.2 | 199.94 | 0.00937 | 0.0479 |
| ARID5B | -4.2 | -2.07 | 1210.52 | 5082.08 | 0.00291 | 0.0418 |
| ICA1L | -4.2 | -2.07 | 66.71 | 279.86 | 0.00416 | 0.0418 |
| DDO | -4.21 | -2.07 | 22.09 | 92.93 | 0.00661 | 0.043 |
| SLC6A16 | -4.2 | -2.07 | 16.2 | 68.01 | 0.00661 | 0.043 |
| CLIC4 | -4.17 | -2.06 | 3340.87 | 13933.2 | 0.00304 | 0.0418 |
| ZNF516 | -4.16 | -2.06 | 253.84 | 1055.04 | 0.00367 | 0.0418 |
| IL11RA | -4.16 | -2.06 | 204.92 | 851.82 | 0.00375 | 0.0418 |
| CCDC3 | -4.16 | -2.06 | 373.92 | 1554.42 | 0.0052 | 0.0418 |
| PTPRN2 | -4.16 | -2.06 | 78.3 | 325.9 | 0.00688 | 0.0433 |
| CELF2 | -4.17 | -2.06 | 282.3 | 1177.46 | 0.00744 | 0.0442 |
| TGM2 | -4.17 | -2.06 | 5105.32 | 21308.42 | 0.00885 | 0.0471 |
| B4GAT1 | -4.13 | -2.05 | 305.96 | 1265.02 | 0.00304 | 0.0418 |
| NAP1L5 | -4.14 | -2.05 | 89.59 | 371.05 | 0.00304 | 0.0418 |
| TBL1X | -4.15 | -2.05 | 887.33 | 3678.45 | 0.00391 | 0.0418 |
| CAVIN3 | -4.13 | -2.05 | 389.4 | 1608.67 | 0.0092 | 0.0476 |
| CAMK1 | -4.1 | -2.04 | 234.46 | 961.33 | 0.00323 | 0.0418 |
| CD99L2 | -4.11 | -2.04 | 702.73 | 2889.06 | 0.00337 | 0.0418 |
| LOC100128164 | -4.12 | -2.04 | 1.07 | 4.41 | 0.0045 | 0.0418 |
| SMARCD3 | -4.12 | -2.04 | 186.24 | 767.96 | 0.00564 | 0.0419 |
| CYGB | -4.12 | -2.04 | 225.33 | 927.97 | 0.00636 | 0.0427 |
| TMEM100 | -4.12 | -2.04 | 41.12 | 169.4 | 0.00661 | 0.043 |
| ARMC4 | -4.11 | -2.04 | 7.26 | 29.79 | 0.00844 | 0.0464 |
| NT5E | -4.11 | -2.04 | 590.93 | 2430.61 | 0.0092 | 0.0476 |
| ARHGEF6 | -4.07 | -2.03 | 203.6 | 828.96 | 0.00461 | 0.0418 |
| LTC4S | -4.07 | -2.03 | 17.81 | 72.53 | 0.00564 | 0.0419 |
| MPL | -4.1 | -2.03 | 12.52 | 51.32 | 0.00623 | 0.0425 |
| C1QTNF9B | -4.1 | -2.03 | 4.48 | 18.37 | 0.00774 | 0.0449 |
| ATP8B4 | -4.06 | -2.02 | 48.02 | 195.17 | 0.00399 | 0.0418 |
| PRRT1 | -4.07 | -2.02 | 45.49 | 184.97 | 0.00416 | 0.0418 |
| CDNF | -4.05 | -2.02 | 9.94 | 40.24 | 0.00442 | 0.0418 |
| NT5DC3 | -4.07 | -2.02 | 269.43 | 1095.76 | 0.0049 | 0.0418 |
| SEMA6B | -4.05 | -2.02 | 182.04 | 736.53 | 0.0052 | 0.0418 |
| GUCY1B3 | -4.07 | -2.02 | 156.79 | 637.8 | 0.00575 | 0.0421 |
| KCNK3 | -4.06 | -2.02 | 58.42 | 237.4 | 0.00611 | 0.0424 |
| NDNF | -4.06 | -2.02 | 35.5 | 144.05 | 0.00955 | 0.0482 |
| ARHGEF17 | -4.03 | -2.01 | 621.33 | 2501.57 | 0.00323 | 0.0418 |
| SNX21 | -4.02 | -2.01 | 318.71 | 1281.59 | 0.00337 | 0.0418 |
| TSPAN4 | -4.03 | -2.01 | 574.77 | 2315.17 | 0.00433 | 0.0418 |
| LOC283392 | -4.03 | -2.01 | 7.38 | 29.7 | 0.00565 | 0.042 |
| SYDE1 | -4.04 | -2.01 | 351.5 | 1418.99 | 0.00648 | 0.043 |
| PCDH10 | -4.03 | -2.01 | 7.94 | 31.96 | 0.00649 | 0.043 |
| OR52N4 | -4.03 | -2.01 | 0.83 | 3.34 | 0.00727 | 0.0439 |
| FAIM2 | -4.04 | -2.01 | 62.75 | 253.45 | 0.00804 | 0.0454 |
| EID1 | -4 | -2 | 1926.3 | 7711.97 | 0.00291 | 0.0418 |
| HOXA10 | -4 | -2 | 521.01 | 2085.8 | 0.00416 | 0.0418 |
| ATL1 | -4.01 | -2 | 122.35 | 490.04 | 0.00564 | 0.0419 |
| PTPRF | 4.07 | 2.02 | 13528.89 | 3327.19 | 0.00297 | 0.0418 |
| GINS3 | 4.05 | 2.02 | 547.17 | 135.24 | 0.00337 | 0.0418 |
| PRIM1 | 4.06 | 2.02 | 384.91 | 94.88 | 0.00375 | 0.0418 |
| CEP72 | 4.05 | 2.02 | 206.97 | 51.07 | 0.00442 | 0.0418 |
| NSD2 | 4.08 | 2.03 | 2920.93 | 715.13 | 0.0033 | 0.0418 |
| **Gene** | **Fold change** | **log2 (Fold change)** | **Mean TPM* (tumor)** | **Mean TPM* (normal)** | **p-value** | **adjusted p-value** |
| TARBP1 | 4.11 | 2.04 | 831.12 | 202.27 | 0.00304 | 0.0418 |
| PRICKLE3 | 4.1 | 2.04 | 370.69 | 90.41 | 0.00433 | 0.0418 |
| DENND1C | 4.12 | 2.04 | 250.85 | 60.81 | 0.00759 | 0.0445 |
| CHAF1B | 4.14 | 2.05 | 641.88 | 155.17 | 0.00317 | 0.0418 |
| TPD52 | 4.14 | 2.05 | 2472.82 | 597.17 | 0.0049 | 0.0418 |
| C19orf23 | 4.14 | 2.05 | 31.95 | 7.72 | 0.00715 | 0.0437 |
| HAUS8 | 4.16 | 2.06 | 454.92 | 109.44 | 0.00297 | 0.0418 |
| C10orf110 | 4.16 | 2.06 | 14.22 | 3.42 | 0.0048 | 0.0418 |
| FBXO41 | 4.16 | 2.06 | 348.84 | 83.87 | 0.00623 | 0.0425 |
| SH3D21 | 4.16 | 2.06 | 198.58 | 47.68 | 0.00868 | 0.0467 |
| DEPDC4 | 4.2 | 2.07 | 12.94 | 3.08 | 0.00636 | 0.0427 |
| POLE | 4.24 | 2.08 | 1960.37 | 462.63 | 0.00291 | 0.0418 |
| SASS6 | 4.22 | 2.08 | 349.95 | 83.01 | 0.00352 | 0.0418 |
| IKBKE | 4.24 | 2.08 | 556.27 | 131.11 | 0.00661 | 0.043 |
| CEP152 | 4.27 | 2.09 | 215.19 | 50.43 | 0.00587 | 0.0421 |
| SKIL | 4.27 | 2.09 | 793.2 | 185.93 | 0.00661 | 0.043 |
| KIAA1841 | 4.27 | 2.1 | 452.37 | 105.88 | 0.00383 | 0.0418 |
| PRKCZ | 4.3 | 2.1 | 749.99 | 174.48 | 0.00416 | 0.0418 |
| KIAA1217 | 4.28 | 2.1 | 2111.59 | 493.12 | 0.00553 | 0.0418 |
| ARHGEF19 | 4.29 | 2.1 | 1176.44 | 274.09 | 0.00587 | 0.0421 |
| GPR113 | 4.29 | 2.1 | 11.35 | 2.64 | 0.00599 | 0.0421 |
| ARHGEF5 | 4.33 | 2.11 | 803.86 | 185.59 | 0.00359 | 0.0418 |
| LRRC8B | 4.33 | 2.11 | 661.92 | 152.97 | 0.00367 | 0.0418 |
| MYO10 | 4.31 | 2.11 | 1958.71 | 454.07 | 0.0047 | 0.0418 |
| LPAR2 | 4.31 | 2.11 | 532.76 | 123.7 | 0.0051 | 0.0418 |
| CCDC15 | 4.36 | 2.12 | 93.23 | 21.38 | 0.0049 | 0.0418 |
| DNMT1 | 4.39 | 2.13 | 3383.01 | 771.26 | 0.00337 | 0.0418 |
| NCAPD3 | 4.37 | 2.13 | 947.65 | 216.86 | 0.00337 | 0.0418 |
| IDH2 | 4.39 | 2.13 | 4676.16 | 1065.4 | 0.00375 | 0.0418 |
| NCAPD2 | 4.42 | 2.14 | 3289.24 | 743.93 | 0.00291 | 0.0418 |
| TOPBP1 | 4.41 | 2.14 | 1999.38 | 453.8 | 0.00323 | 0.0418 |
| CDCA4 | 4.4 | 2.14 | 1351.27 | 307.14 | 0.0033 | 0.0418 |
| RPP25 | 4.41 | 2.14 | 1004.84 | 227.72 | 0.0052 | 0.0418 |
| CDS1 | 4.4 | 2.14 | 911.4 | 207.31 | 0.00542 | 0.0418 |
| NUDT5 | 4.45 | 2.15 | 1132.51 | 254.4 | 0.00291 | 0.0418 |
| TIGD3 | 4.43 | 2.15 | 34.26 | 7.73 | 0.0082 | 0.0456 |
| KIF22 | 4.46 | 2.16 | 2627.45 | 589.25 | 0.00291 | 0.0418 |
| PTPN6 | 4.48 | 2.16 | 1316.38 | 293.57 | 0.0033 | 0.0418 |
| SREBF1 | 4.47 | 2.16 | 5684.5 | 1272.98 | 0.00352 | 0.0418 |
| TUBB8 | 4.48 | 2.16 | 16.22 | 3.62 | 0.00885 | 0.0471 |
| ZWILCH | 4.51 | 2.17 | 857.3 | 189.99 | 0.00291 | 0.0418 |
| SEMA3F | 4.5 | 2.17 | 3244.49 | 720.83 | 0.00599 | 0.0421 |
| PTPN3 | 4.48 | 2.17 | 981.84 | 218.92 | 0.00674 | 0.0432 |
| VAMP8 | 4.49 | 2.17 | 3585.73 | 798.66 | 0.00715 | 0.0437 |
| RAP2B | 4.51 | 2.17 | 2771.43 | 613.97 | 0.00759 | 0.0445 |
| FLVCR1 | 4.52 | 2.18 | 141.74 | 31.36 | 0.00367 | 0.0418 |
| NCEH1 | 4.55 | 2.18 | 897.77 | 197.44 | 0.00773 | 0.0449 |
| SKP2 | 4.58 | 2.19 | 371.01 | 81.08 | 0.00433 | 0.0418 |
| DENND2D | 4.56 | 2.19 | 827.03 | 181.45 | 0.0047 | 0.0418 |
| RHBDF2 | 4.56 | 2.19 | 1138.07 | 249.42 | 0.0047 | 0.0418 |
| DLEU2 | 4.57 | 2.19 | 112.08 | 24.5 | 0.0049 | 0.0418 |
| ERO1A | 4.56 | 2.19 | 2290.69 | 501.8 | 0.00955 | 0.0482 |
| LMNB2 | 4.59 | 2.2 | 4048.16 | 882.63 | 0.00291 | 0.0418 |
| MXD3 | 4.61 | 2.2 | 479.5 | 104.07 | 0.00359 | 0.0418 |
| RELT | 4.58 | 2.2 | 362.27 | 79.11 | 0.00433 | 0.0418 |
| POLD1 | 4.67 | 2.22 | 1849.13 | 396.23 | 0.00317 | 0.0418 |
| FASN | 4.65 | 2.22 | 5214.15 | 1120.19 | 0.00636 | 0.0427 |
| SIPA1L3 | 4.68 | 2.23 | 1552.07 | 331.93 | 0.0031 | 0.0418 |
| MMS22L | 4.69 | 2.23 | 474.58 | 101.17 | 0.00352 | 0.0418 |
| TPI1P2 | 4.69 | 2.23 | 15.89 | 3.39 | 0.00611 | 0.0424 |
| SPINT2 | 4.7 | 2.23 | 16827.16 | 3577.29 | 0.00701 | 0.0434 |
| RAB11FIP4 | 4.71 | 2.23 | 720.5 | 153.1 | 0.00789 | 0.0452 |
| PSRC1 | 4.73 | 2.24 | 358.94 | 75.85 | 0.00399 | 0.0418 |
| SCO2 | 4.71 | 2.24 | 1478.37 | 313.77 | 0.00442 | 0.0418 |
| RUNX1 | 4.74 | 2.24 | 1970.1 | 415.67 | 0.0049 | 0.0418 |
| CORO2A | 4.71 | 2.24 | 1322.8 | 280.68 | 0.00531 | 0.0418 |
| GCLM | 4.74 | 2.24 | 509.52 | 107.59 | 0.00564 | 0.0419 |
| C1orf53 | 4.74 | 2.24 | 67.47 | 14.24 | 0.00587 | 0.0421 |
| STXBP2 | 4.75 | 2.25 | 2083.05 | 438.56 | 0.00424 | 0.0418 |
| TBC1D2 | 4.76 | 2.25 | 1577.19 | 331.59 | 0.00868 | 0.0467 |
| CD101 | 4.8 | 2.26 | 81.17 | 16.9 | 0.00416 | 0.0418 |
| CPT1B | 4.79 | 2.26 | 595.09 | 124.11 | 0.00553 | 0.0418 |
| CEP85 | 4.83 | 2.27 | 671.3 | 138.97 | 0.00383 | 0.0418 |
| CKS1B | 4.85 | 2.28 | 2051.47 | 422.88 | 0.00291 | 0.0418 |
| SMC2 | 4.85 | 2.28 | 1444.86 | 297.62 | 0.00337 | 0.0418 |
| AMMECR1 | 4.86 | 2.28 | 765.63 | 157.65 | 0.00367 | 0.0418 |
| TDRKH | 4.85 | 2.28 | 340.24 | 70.18 | 0.00623 | 0.0425 |
| OPLAH | 4.86 | 2.28 | 480.95 | 98.99 | 0.00701 | 0.0434 |
| DARS2 | 4.9 | 2.29 | 773.08 | 157.64 | 0.00291 | 0.0418 |
| TRAIP | 4.89 | 2.29 | 261.18 | 53.4 | 0.00297 | 0.0418 |
| ANO8 | 4.9 | 2.29 | 367.12 | 74.85 | 0.00575 | 0.0421 |
| MB21D1 | 4.9 | 2.29 | 238.81 | 48.76 | 0.00773 | 0.0449 |
| FAAP24 | 4.93 | 2.3 | 87.87 | 17.82 | 0.00375 | 0.0418 |
| C1orf59 | 4.93 | 2.3 | 583.59 | 118.39 | 0.00451 | 0.0418 |
| NCRNA00105 | 4.91 | 2.3 | 127.22 | 25.89 | 0.00885 | 0.0471 |
| EFCAB11 | 4.96 | 2.31 | 128.22 | 25.87 | 0.00291 | 0.0418 |
| TEDC1 | 4.96 | 2.31 | 647.79 | 130.64 | 0.00297 | 0.0418 |
| DONSON | 4.97 | 2.31 | 668.62 | 134.41 | 0.00297 | 0.0418 |
| RFC4 | 4.97 | 2.31 | 1481.7 | 297.93 | 0.0031 | 0.0418 |
| DDX12 | 4.96 | 2.31 | 286.89 | 57.83 | 0.00345 | 0.0418 |
| PRR19 | 4.95 | 2.31 | 87.33 | 17.65 | 0.00345 | 0.0418 |
| EPS8L2 | 4.96 | 2.31 | 3176.06 | 640.38 | 0.00375 | 0.0418 |
| EBP | 4.97 | 2.31 | 2493.78 | 501.63 | 0.00391 | 0.0418 |
| N4BP3 | 4.96 | 2.31 | 450.98 | 90.97 | 0.00804 | 0.0454 |
| CENPL | 5 | 2.32 | 284.64 | 56.88 | 0.00291 | 0.0418 |
| NBEAL2 | 4.99 | 2.32 | 2140.86 | 429.23 | 0.0031 | 0.0418 |
| TRIM59 | 4.99 | 2.32 | 501.74 | 100.58 | 0.00531 | 0.0418 |
| XRCC3 | 5.04 | 2.33 | 793.86 | 157.36 | 0.00304 | 0.0418 |
| TMEM132A | 5.01 | 2.33 | 2030.49 | 405.12 | 0.00674 | 0.0432 |
| ENTPD7 | 5.07 | 2.34 | 509.55 | 100.43 | 0.00542 | 0.0418 |
| ITGB4 | 5.06 | 2.34 | 12422.87 | 2455.64 | 0.0082 | 0.0456 |
| PAFAH1B3 | 5.09 | 2.35 | 1497.94 | 294.07 | 0.00304 | 0.0418 |
| RMI1 | 5.09 | 2.35 | 500.87 | 98.31 | 0.0033 | 0.0418 |
| COMTD1 | 5.11 | 2.35 | 579.95 | 113.49 | 0.00451 | 0.0418 |
| SQLE | 5.09 | 2.35 | 1983.37 | 389.61 | 0.00688 | 0.0433 |
| ARHGEF39 | 5.13 | 2.36 | 347.22 | 67.66 | 0.0033 | 0.0418 |
| CENPH | 5.12 | 2.36 | 540.12 | 105.42 | 0.00337 | 0.0418 |
| DEF6 | 5.14 | 2.36 | 897.48 | 174.66 | 0.00416 | 0.0418 |
| SEMA4A | 5.15 | 2.36 | 1011.07 | 196.43 | 0.00531 | 0.0418 |
| STAP2 | 5.15 | 2.36 | 1442.09 | 280.18 | 0.00575 | 0.0421 |
| CRB3 | 5.13 | 2.36 | 477.75 | 93.08 | 0.00836 | 0.046 |
| CHRNA5 | 5.14 | 2.36 | 158.76 | 30.9 | 0.00974 | 0.0487 |
| MCM5 | 5.17 | 2.37 | 4234.55 | 819.74 | 0.00304 | 0.0418 |
| PRAG1 | 5.17 | 2.37 | 631.63 | 122.27 | 0.00424 | 0.0418 |
| SRD5A1 | 5.18 | 2.37 | 836.83 | 161.59 | 0.00451 | 0.0418 |
| STAT1 | 5.16 | 2.37 | 8856.56 | 1716.97 | 0.00599 | 0.0421 |
| AP1M2 | 5.18 | 2.37 | 1668.71 | 322.18 | 0.00661 | 0.043 |
| BRWD3 | 5.17 | 2.37 | 238.79 | 46.19 | 0.00836 | 0.046 |
| RPL39L | 5.17 | 2.37 | 890 | 172.26 | 0.0092 | 0.0476 |
| TUFT1 | 5.22 | 2.38 | 1305.04 | 250.13 | 0.00359 | 0.0418 |
| INCENP | 5.22 | 2.38 | 742.11 | 142.25 | 0.00375 | 0.0418 |
| FDPSL2A | 5.21 | 2.38 | 10.04 | 1.93 | 0.00885 | 0.0471 |
| AURKAPS1 | 5.24 | 2.39 | 23.41 | 4.47 | 0.00317 | 0.0418 |
| OAZ3 | 5.23 | 2.39 | 31.01 | 5.93 | 0.00599 | 0.0421 |
| HPSE | 5.26 | 2.4 | 215.01 | 40.87 | 0.00937 | 0.0479 |
| CIT | 5.31 | 2.41 | 991.21 | 186.8 | 0.00416 | 0.0418 |
| PRKX | 5.31 | 2.41 | 1595.98 | 300.36 | 0.00587 | 0.0421 |
| LOC80154 | 5.32 | 2.41 | 1762.41 | 331.5 | 0.00623 | 0.0425 |
| INTS7 | 5.4 | 2.43 | 855.23 | 158.42 | 0.00291 | 0.0418 |
| TTF2 | 5.37 | 2.43 | 596.85 | 111.09 | 0.00424 | 0.0418 |
| C11orf35 | 5.4 | 2.43 | 57.54 | 10.65 | 0.00433 | 0.0418 |
| GOLGA2P5 | 5.38 | 2.43 | 287.37 | 53.41 | 0.00789 | 0.0452 |
| MGME1 | 5.45 | 2.45 | 901.06 | 165.32 | 0.00297 | 0.0418 |
| RPS6KA1 | 5.46 | 2.45 | 2012.66 | 368.44 | 0.00367 | 0.0418 |
| KIFC2 | 5.5 | 2.46 | 759.59 | 138.17 | 0.00317 | 0.0418 |
| JUP | 5.49 | 2.46 | 26037.35 | 4738.94 | 0.00451 | 0.0418 |
| NDC1 | 5.53 | 2.47 | 501.45 | 90.64 | 0.0031 | 0.0418 |
| ABHD3 | 5.52 | 2.47 | 682.14 | 123.52 | 0.0033 | 0.0418 |
| S100A11 | 5.56 | 2.47 | 29183.86 | 5250.27 | 0.005 | 0.0418 |
| RCC2 | 5.58 | 2.48 | 7970.73 | 1427.87 | 0.00291 | 0.0418 |
| C4orf21 | 5.58 | 2.48 | 225.12 | 40.35 | 0.00352 | 0.0418 |
| ZDHHC23 | 5.59 | 2.48 | 337.95 | 60.47 | 0.00359 | 0.0418 |
| UBE2S | 5.57 | 2.48 | 1349.15 | 242.18 | 0.0049 | 0.0418 |
| SEC14L2 | 5.58 | 2.48 | 589.71 | 105.75 | 0.0092 | 0.0476 |
| ATP2A1 | 5.6 | 2.49 | 25.22 | 4.5 | 0.0073 | 0.0439 |
| DDR1 | 5.67 | 2.5 | 9137.85 | 1612.22 | 0.00317 | 0.0418 |
| GPT2 | 5.7 | 2.51 | 1073.72 | 188.24 | 0.00359 | 0.0418 |
| MTBP | 5.69 | 2.51 | 230.47 | 40.48 | 0.00407 | 0.0418 |
| ANKRD9 | 5.7 | 2.51 | 286.04 | 50.16 | 0.00451 | 0.0418 |
| KIAA1522 | 5.74 | 2.52 | 6247.29 | 1087.86 | 0.00304 | 0.0418 |
| LIG1 | 5.73 | 2.52 | 2183.32 | 381.12 | 0.00304 | 0.0418 |
| GMNN | 5.75 | 2.52 | 965.95 | 167.86 | 0.0031 | 0.0418 |
| LSR | 5.73 | 2.52 | 5561.46 | 970.75 | 0.00317 | 0.0418 |
| CENPW | 5.73 | 2.52 | 1397.66 | 243.82 | 0.0047 | 0.0418 |
| RNF43 | 5.73 | 2.52 | 590.35 | 103 | 0.00553 | 0.0418 |
| DSN1 | 5.82 | 2.54 | 1054 | 181.15 | 0.00291 | 0.0418 |
| KNSTRN | 5.85 | 2.55 | 561.02 | 95.93 | 0.00291 | 0.0418 |
| NHSL1 | 5.88 | 2.55 | 598.7 | 101.9 | 0.00375 | 0.0418 |
| C1orf97 | 5.84 | 2.55 | 145.49 | 24.9 | 0.00391 | 0.0418 |
| C5orf34 | 5.91 | 2.56 | 138.12 | 23.37 | 0.00291 | 0.0418 |
| FBXO43 | 5.88 | 2.56 | 30.9 | 5.25 | 0.00715 | 0.0437 |
| MANEAL | 5.88 | 2.56 | 391.95 | 66.61 | 0.0073 | 0.0439 |
| LOC100144603 | 5.9 | 2.56 | 16.28 | 2.76 | 0.00937 | 0.0479 |
| TUBA1C | 5.95 | 2.57 | 11730.91 | 1970.99 | 0.00291 | 0.0418 |
| OR2A7 | 5.95 | 2.57 | 218.97 | 36.83 | 0.00367 | 0.0418 |
| BDH1 | 5.96 | 2.57 | 981.94 | 164.8 | 0.00564 | 0.0419 |
| AGMAT | 5.94 | 2.57 | 88.02 | 14.82 | 0.00868 | 0.0467 |
| ODF3B | 5.94 | 2.57 | 386.05 | 64.94 | 0.00868 | 0.0467 |
| CHTF18 | 5.98 | 2.58 | 1016.44 | 169.95 | 0.00291 | 0.0418 |
| TCF19 | 5.99 | 2.58 | 1902.07 | 317.51 | 0.0031 | 0.0418 |
| GPSM2 | 5.99 | 2.58 | 1340.79 | 223.78 | 0.00337 | 0.0418 |
| NDC80 | 6.02 | 2.59 | 713.82 | 118.57 | 0.00291 | 0.0418 |
| ESRP2 | 6.07 | 2.6 | 1614.13 | 265.96 | 0.0031 | 0.0418 |
| C21orf58 | 6.07 | 2.6 | 250.58 | 41.29 | 0.00337 | 0.0418 |
| LOC100128191 | 6.08 | 2.6 | 204.85 | 33.68 | 0.00345 | 0.0418 |
| **Gene** | **Fold change** | **log2 (Fold change)** | **Mean TPM* (tumor)** | **Mean TPM* (normal)** | **p-value** | **adjusted p-value** |
| RBL1 | 6.07 | 2.6 | 329.75 | 54.29 | 0.00587 | 0.0421 |
| RBM47 | 6.06 | 2.6 | 1692.68 | 279.32 | 0.00661 | 0.043 |
| VDR | 6.11 | 2.61 | 1079.29 | 176.51 | 0.00564 | 0.0419 |
| PGD | 6.17 | 2.62 | 6112.03 | 991.19 | 0.0033 | 0.0418 |
| ELMO3 | 6.13 | 2.62 | 909.99 | 148.46 | 0.00575 | 0.0421 |
| SEMA4B | 6.17 | 2.62 | 8116.52 | 1316.19 | 0.00611 | 0.0424 |
| DHFR | 6.2 | 2.63 | 664.17 | 107.09 | 0.0033 | 0.0418 |
| ARHGEF35 | 6.19 | 2.63 | 693.69 | 112.1 | 0.00542 | 0.0418 |
| C1orf112 | 6.23 | 2.64 | 394.11 | 63.29 | 0.00291 | 0.0418 |
| RELL2 | 6.24 | 2.64 | 116.4 | 18.67 | 0.00636 | 0.0427 |
| NFE2L3 | 6.25 | 2.64 | 770.67 | 123.36 | 0.00744 | 0.0442 |
| TCF7 | 6.29 | 2.65 | 469.96 | 74.75 | 0.00661 | 0.043 |
| GEMIN8P4 | 6.32 | 2.66 | 64.07 | 10.14 | 0.00359 | 0.0418 |
| TNFRSF25 | 6.37 | 2.67 | 419.25 | 65.8 | 0.0052 | 0.0418 |
| TET3 | 6.41 | 2.68 | 1003.96 | 156.74 | 0.00337 | 0.0418 |
| RCAN3 | 6.39 | 2.68 | 137.02 | 21.44 | 0.00391 | 0.0418 |
| ACOT11 | 6.43 | 2.68 | 341.43 | 53.12 | 0.00433 | 0.0418 |
| DNMT3B | 6.41 | 2.68 | 218.38 | 34.08 | 0.00648 | 0.043 |
| CSTB | 6.41 | 2.68 | 15962 | 2488.74 | 0.00789 | 0.0452 |
| SLC25A10 | 6.48 | 2.7 | 1163.48 | 179.62 | 0.00304 | 0.0418 |
| CHEK1 | 6.56 | 2.71 | 470.81 | 71.78 | 0.00291 | 0.0418 |
| KIF20B | 6.54 | 2.71 | 648.79 | 99.13 | 0.0033 | 0.0418 |
| MCM8 | 6.55 | 2.71 | 592.37 | 90.42 | 0.00337 | 0.0418 |
| KPNA2 | 6.63 | 2.73 | 4356.92 | 657.09 | 0.00291 | 0.0418 |
| C6orf132 | 6.62 | 2.73 | 405.28 | 61.25 | 0.0073 | 0.0439 |
| ANKEF1 | 6.67 | 2.74 | 212.51 | 31.88 | 0.00317 | 0.0418 |
| AP1S3 | 6.69 | 2.74 | 318.54 | 47.63 | 0.00442 | 0.0418 |
| TUBA4A | 6.67 | 2.74 | 4257.4 | 638.41 | 0.00674 | 0.0432 |
| HIST1H2BD | 6.75 | 2.75 | 478.29 | 70.86 | 0.00661 | 0.043 |
| FHDC1 | 6.78 | 2.76 | 468.1 | 69.07 | 0.00367 | 0.0418 |
| PSD4 | 6.83 | 2.77 | 1151.62 | 168.57 | 0.00359 | 0.0418 |
| F11R | 6.88 | 2.78 | 9831.43 | 1429.84 | 0.00317 | 0.0418 |
| RNASEH2A | 6.94 | 2.79 | 1664.31 | 239.84 | 0.00291 | 0.0418 |
| CYP2D7 | 6.92 | 2.79 | 36.04 | 5.21 | 0.00424 | 0.0418 |
| ESPN | 6.92 | 2.79 | 975.82 | 141.05 | 0.00955 | 0.0482 |
| B3GNT5 | 6.95 | 2.8 | 2248.04 | 323.55 | 0.00611 | 0.0424 |
| SPTBN5 | 6.95 | 2.8 | 232.56 | 33.47 | 0.00992 | 0.0488 |
| NCAPG2 | 6.99 | 2.81 | 1247.6 | 178.37 | 0.00297 | 0.0418 |
| USP18 | 7.02 | 2.81 | 650 | 92.57 | 0.00375 | 0.0418 |
| PCNA | 7.12 | 2.83 | 6486.54 | 911.5 | 0.00291 | 0.0418 |
| IFI30 | 7.09 | 2.83 | 4798.83 | 676.6 | 0.0052 | 0.0418 |
| IRF5 | 7.11 | 2.83 | 760.04 | 106.96 | 0.0082 | 0.0456 |
| CHAF1A | 7.17 | 2.84 | 1399.27 | 195.19 | 0.00291 | 0.0418 |
| CELSR1 | 7.21 | 2.85 | 2862.5 | 396.93 | 0.00416 | 0.0418 |
| SYK | 7.2 | 2.85 | 1607.38 | 223.11 | 0.00564 | 0.0419 |
| SLC9A7 | 7.19 | 2.85 | 86.57 | 12.03 | 0.00661 | 0.043 |
| MFSD2A | 7.23 | 2.85 | 497.25 | 68.76 | 0.00902 | 0.0474 |
| MST1P2 | 7.24 | 2.86 | 110.31 | 15.23 | 0.00542 | 0.0418 |
| KIF24 | 7.3 | 2.87 | 261.17 | 35.78 | 0.00291 | 0.0418 |
| CAMSAP3 | 7.31 | 2.87 | 688.1 | 94.18 | 0.00701 | 0.0434 |
| CLDN7 | 7.32 | 2.87 | 3938.21 | 538.02 | 0.00759 | 0.0445 |
| DSCR9 | 7.31 | 2.87 | 4.18 | 0.57 | 0.00955 | 0.0482 |
| SH3BP1 | 7.42 | 2.89 | 1378.05 | 185.84 | 0.00323 | 0.0418 |
| SAMD10 | 7.48 | 2.9 | 231.53 | 30.97 | 0.00352 | 0.0418 |
| IKZF2 | 7.45 | 2.9 | 648.39 | 87.05 | 0.00902 | 0.0474 |
| FAM110A | 7.5 | 2.91 | 971.32 | 129.52 | 0.00317 | 0.0418 |
| DENND1B | 7.52 | 2.91 | 88.24 | 11.74 | 0.00337 | 0.0418 |
| SNORD1C | 7.53 | 2.91 | 37.85 | 5.03 | 0.00451 | 0.0418 |
| WWC1 | 7.49 | 2.91 | 1113.2 | 148.59 | 0.00852 | 0.0464 |
| REEP4 | 7.55 | 2.92 | 1847.11 | 244.51 | 0.00297 | 0.0418 |
| PRSS16 | 7.55 | 2.92 | 470.1 | 62.28 | 0.00442 | 0.0418 |
| P2RY2 | 7.59 | 2.92 | 333.18 | 43.88 | 0.0092 | 0.0476 |
| MYO19 | 7.63 | 2.93 | 1898.54 | 248.69 | 0.00304 | 0.0418 |
| MAP3K21 | 7.64 | 2.93 | 333.39 | 43.65 | 0.00323 | 0.0418 |
| CCDC138 | 7.61 | 2.93 | 109.63 | 14.4 | 0.00359 | 0.0418 |
| NUP62CL | 7.63 | 2.93 | 125.65 | 16.46 | 0.00611 | 0.0424 |
| BORA | 7.69 | 2.94 | 375.82 | 48.85 | 0.00291 | 0.0418 |
| SLC29A2 | 7.68 | 2.94 | 377.88 | 49.22 | 0.00367 | 0.0418 |
| OVOL2 | 7.69 | 2.94 | 271.4 | 35.31 | 0.00611 | 0.0424 |
| RCC1 | 7.75 | 2.95 | 1636.39 | 211.03 | 0.00291 | 0.0418 |
| SGO2 | 7.71 | 2.95 | 398.24 | 51.68 | 0.00291 | 0.0418 |
| C8orf73 | 7.74 | 2.95 | 783.6 | 101.18 | 0.0073 | 0.0439 |
| C12orf48 | 7.79 | 2.96 | 313.59 | 40.23 | 0.00291 | 0.0418 |
| LOC100133991 | 7.78 | 2.96 | 16.18 | 2.08 | 0.0052 | 0.0418 |
| IFNLR1 | 7.86 | 2.97 | 222.15 | 28.27 | 0.00701 | 0.0434 |
| DBNDD1 | 7.82 | 2.97 | 520 | 66.49 | 0.00759 | 0.0445 |
| CRYBG1 | 7.9 | 2.98 | 2518.29 | 318.86 | 0.00461 | 0.0418 |
| CKAP2 | 7.97 | 2.99 | 1258.3 | 157.95 | 0.00291 | 0.0418 |
| ECE2 | 7.93 | 2.99 | 451.25 | 56.9 | 0.00291 | 0.0418 |
| HMGA1 | 7.95 | 2.99 | 7150.16 | 898.9 | 0.00399 | 0.0418 |
| TSTD1 | 8 | 3 | 917.31 | 114.71 | 0.00352 | 0.0418 |
| MARVELD3 | 8.03 | 3 | 402.54 | 50.14 | 0.00383 | 0.0418 |
| KRTCAP3 | 8.02 | 3 | 828.61 | 103.35 | 0.00542 | 0.0418 |
| PLEKHH1 | 7.99 | 3 | 396.86 | 49.65 | 0.00701 | 0.0434 |
| MAP7 | 8.06 | 3.01 | 1158.64 | 143.84 | 0.00291 | 0.0418 |
| SERINC2 | 8.03 | 3.01 | 5424.19 | 675.08 | 0.0082 | 0.0456 |
| CCDC64B | 8.12 | 3.02 | 818.78 | 100.78 | 0.00868 | 0.0467 |
| BAIAP2L1 | 8.14 | 3.03 | 1966.93 | 241.55 | 0.00304 | 0.0418 |
| LRP8 | 8.19 | 3.03 | 372.54 | 45.47 | 0.00451 | 0.0418 |
| HIST1H1E | 8.17 | 3.03 | 13.53 | 1.65 | 0.00564 | 0.0419 |
| GGH | 8.24 | 3.04 | 1432.23 | 173.89 | 0.00375 | 0.0418 |
| NPL | 8.21 | 3.04 | 352.08 | 42.9 | 0.00531 | 0.0418 |
| IL10 | 8.21 | 3.04 | 17.03 | 2.07 | 0.0092 | 0.0476 |
| FEN1 | 8.3 | 3.05 | 2083.91 | 251.08 | 0.00291 | 0.0418 |
| OSBPL3 | 8.27 | 3.05 | 1060.64 | 128.18 | 0.00297 | 0.0418 |
| MYO5B | 8.28 | 3.05 | 1540.74 | 186 | 0.00674 | 0.0432 |
| RAD54B | 8.36 | 3.06 | 243.4 | 29.12 | 0.00291 | 0.0418 |
| EFNA3 | 8.31 | 3.06 | 606.42 | 72.93 | 0.00852 | 0.0464 |
| CDCA7 | 8.42 | 3.07 | 1422.44 | 168.99 | 0.00375 | 0.0418 |
| LBX2 | 8.41 | 3.07 | 22.78 | 2.71 | 0.00688 | 0.0433 |
| MFSD2B | 8.5 | 3.09 | 114.33 | 13.46 | 0.00461 | 0.0418 |
| NUAK2 | 8.49 | 3.09 | 716.86 | 84.45 | 0.0048 | 0.0418 |
| LMTK3 | 8.5 | 3.09 | 247.71 | 29.13 | 0.00937 | 0.0479 |
| OAS2 | 8.58 | 3.1 | 2946.26 | 343.35 | 0.00937 | 0.0479 |
| ARHGEF16 | 8.63 | 3.11 | 1214.14 | 140.67 | 0.00442 | 0.0418 |
| BRCA1 | 8.7 | 3.12 | 763.73 | 87.73 | 0.00297 | 0.0418 |
| DDIAS | 8.69 | 3.12 | 295.92 | 34.04 | 0.00304 | 0.0418 |
| FAM83G | 8.67 | 3.12 | 437.35 | 50.42 | 0.0082 | 0.0456 |
| CLDN4 | 8.68 | 3.12 | 7519.27 | 866.23 | 0.00902 | 0.0474 |
| LRRC16B | 8.72 | 3.12 | 31.73 | 3.64 | 0.00955 | 0.0482 |
| IGSF3 | 8.77 | 3.13 | 2623.83 | 299.17 | 0.00424 | 0.0418 |
| LOC441294 | 8.78 | 3.13 | 93.75 | 10.68 | 0.00433 | 0.0418 |
| ST6GALNAC2 | 8.73 | 3.13 | 1182.98 | 135.53 | 0.00564 | 0.0419 |
| PROM2 | 8.78 | 3.13 | 5047.79 | 574.69 | 0.00789 | 0.0452 |
| GSDMB | 8.81 | 3.14 | 568.29 | 64.48 | 0.00715 | 0.0437 |
| CNKSR1 | 8.89 | 3.15 | 751.48 | 84.49 | 0.00345 | 0.0418 |
| JPT1 | 8.93 | 3.16 | 4283.41 | 479.52 | 0.00297 | 0.0418 |
| TYMSOS | 8.94 | 3.16 | 46.52 | 5.2 | 0.00367 | 0.0418 |
| SYTL1 | 8.96 | 3.16 | 1569.78 | 175.2 | 0.00661 | 0.043 |
| GCSAM | 9.02 | 3.17 | 71.93 | 7.97 | 0.00636 | 0.0427 |
| TUBB3 | 8.98 | 3.17 | 1377.59 | 153.34 | 0.0092 | 0.0476 |
| TGFA | 9.06 | 3.18 | 1137.62 | 125.62 | 0.00674 | 0.0432 |
| PODXL2 | 9.14 | 3.19 | 1285.6 | 140.72 | 0.00587 | 0.0421 |
| KCNK13 | 9.15 | 3.19 | 26.35 | 2.88 | 0.00955 | 0.0482 |
| LYPD5 | 9.15 | 3.19 | 497.3 | 54.36 | 0.00955 | 0.0482 |
| CHMP4C | 9.19 | 3.2 | 529.12 | 57.6 | 0.00345 | 0.0418 |
| ABCA7 | 9.19 | 3.2 | 893.9 | 97.22 | 0.00359 | 0.0418 |
| CDCP1 | 9.27 | 3.21 | 3000.48 | 323.52 | 0.00367 | 0.0418 |
| LPAR5 | 9.28 | 3.21 | 326.11 | 35.13 | 0.00564 | 0.0419 |
| LAMB3 | 9.27 | 3.21 | 12404.96 | 1338.75 | 0.00955 | 0.0482 |
| WDHD1 | 9.37 | 3.23 | 711.89 | 75.97 | 0.00291 | 0.0418 |
| DHCR24 | 9.36 | 3.23 | 12366.58 | 1321.45 | 0.0033 | 0.0418 |
| C3orf52 | 9.36 | 3.23 | 360.93 | 38.58 | 0.00367 | 0.0418 |
| PHLDA2 | 9.36 | 3.23 | 710.93 | 75.94 | 0.00461 | 0.0418 |
| PABPC1L | 9.45 | 3.24 | 480.6 | 50.83 | 0.0031 | 0.0418 |
| PPP1R13L | 9.47 | 3.24 | 3149.32 | 332.48 | 0.00383 | 0.0418 |
| ERBB3 | 9.44 | 3.24 | 3163.2 | 334.95 | 0.00433 | 0.0418 |
| SLC12A8 | 9.52 | 3.25 | 466.87 | 49.06 | 0.00542 | 0.0418 |
| MARVELD2 | 9.61 | 3.26 | 368.59 | 38.36 | 0.00297 | 0.0418 |
| MREG | 9.6 | 3.26 | 698.22 | 72.69 | 0.00304 | 0.0418 |
| WDR76 | 9.58 | 3.26 | 652.79 | 68.14 | 0.0031 | 0.0418 |
| ITPR3 | 9.61 | 3.26 | 4476.6 | 465.91 | 0.00375 | 0.0418 |
| ANKRD22 | 9.6 | 3.26 | 658.03 | 68.55 | 0.00974 | 0.0487 |
| SH2D3A | 9.64 | 3.27 | 792.95 | 82.23 | 0.00433 | 0.0418 |
| OAS1 | 9.64 | 3.27 | 2313.57 | 240 | 0.00575 | 0.0421 |
| BRI3BP | 9.68 | 3.28 | 230.49 | 23.81 | 0.00291 | 0.0418 |
| LRRC8E | 9.72 | 3.28 | 521 | 53.58 | 0.0033 | 0.0418 |
| CELSR2 | 9.79 | 3.29 | 3272.27 | 334.28 | 0.00352 | 0.0418 |
| RIBC2 | 9.75 | 3.29 | 219.92 | 22.55 | 0.00542 | 0.0418 |
| KCNC3 | 9.76 | 3.29 | 176.01 | 18.04 | 0.00852 | 0.0464 |
| CDC7 | 9.87 | 3.3 | 654.03 | 66.28 | 0.00291 | 0.0418 |
| CCDC18 | 9.85 | 3.3 | 153.42 | 15.57 | 0.00297 | 0.0418 |
| POC1A | 9.87 | 3.3 | 467.2 | 47.35 | 0.00297 | 0.0418 |
| HOOK1 | 9.91 | 3.31 | 986.74 | 99.6 | 0.00345 | 0.0418 |
| LOC339674 | 9.93 | 3.31 | 11.15 | 1.12 | 0.00648 | 0.043 |
| GEN1 | 9.97 | 3.32 | 736.63 | 73.9 | 0.00291 | 0.0418 |
| FAM110C | 10.01 | 3.32 | 83.46 | 8.33 | 0.00902 | 0.0474 |
| MAD2L1 | 10.16 | 3.34 | 812.19 | 79.98 | 0.00291 | 0.0418 |
| BLNK | 10.1 | 3.34 | 358.01 | 35.45 | 0.00636 | 0.0427 |
| STON2 | 10.14 | 3.34 | 756.02 | 74.59 | 0.00759 | 0.0445 |
| PLA2G7 | 10.14 | 3.34 | 156.6 | 15.44 | 0.00992 | 0.0488 |
| C9orf163 | 10.2 | 3.35 | 14.84 | 1.45 | 0.00383 | 0.0418 |
| MPZL3 | 10.27 | 3.36 | 41.34 | 4.02 | 0.00804 | 0.0454 |
| SLC9A3R1 | 10.34 | 3.37 | 5931.25 | 573.88 | 0.00391 | 0.0418 |
| LTB4R | 10.33 | 3.37 | 1093.35 | 105.88 | 0.00553 | 0.0418 |
| NOD2 | 10.34 | 3.37 | 235.03 | 22.73 | 0.00599 | 0.0421 |
| DUOX1 | 10.37 | 3.37 | 1945.78 | 187.69 | 0.00885 | 0.0471 |
| TACC3 | 10.38 | 3.38 | 2184.51 | 210.41 | 0.00291 | 0.0418 |
| GRB7 | 10.41 | 3.38 | 1670.08 | 160.43 | 0.00345 | 0.0418 |
| TONSL | 10.59 | 3.4 | 930.01 | 87.85 | 0.00291 | 0.0418 |
| CBLC | 10.54 | 3.4 | 984.87 | 93.41 | 0.00542 | 0.0418 |
| TESMIN | 10.62 | 3.41 | 130.61 | 12.3 | 0.0049 | 0.0418 |
| FOXP3 | 10.61 | 3.41 | 83.72 | 7.89 | 0.00661 | 0.043 |
| ZNF367 | 10.68 | 3.42 | 555.63 | 52.03 | 0.0031 | 0.0418 |
| TLCD1 | 10.78 | 3.43 | 241.57 | 22.41 | 0.00323 | 0.0418 |
| **Gene** | **Fold change** | **log2 (Fold change)** | **Mean TPM* (tumor)** | **Mean TPM* (normal)** | **p-value** | **adjusted p-value** |
| CDKN2B | 11.03 | 3.46 | 2228.41 | 201.95 | 0.0049 | 0.0418 |
| TMPRSS13 | 11.03 | 3.46 | 567.93 | 51.51 | 0.00701 | 0.0434 |
| TIMELESS | 11.09 | 3.47 | 2374.82 | 214.05 | 0.00291 | 0.0418 |
| SOX9 | 11.1 | 3.47 | 1495.67 | 134.79 | 0.00885 | 0.0471 |
| NPAS1 | 11.16 | 3.48 | 29.66 | 2.66 | 0.0073 | 0.0439 |
| FXYD3 | 11.26 | 3.49 | 10140.83 | 900.62 | 0.00575 | 0.0421 |
| CENPN | 11.28 | 3.5 | 940.97 | 83.43 | 0.00291 | 0.0418 |
| FANCD2 | 11.34 | 3.5 | 673.82 | 59.43 | 0.00291 | 0.0418 |
| CXADR | 11.34 | 3.5 | 529.65 | 46.71 | 0.00359 | 0.0418 |
| TMEM184A | 11.3 | 3.5 | 1021.76 | 90.45 | 0.00531 | 0.0418 |
| KNTC1 | 11.41 | 3.51 | 1215.97 | 106.54 | 0.00291 | 0.0418 |
| C19orf57 | 11.4 | 3.51 | 338.63 | 29.69 | 0.00648 | 0.043 |
| LOC442459 | 11.47 | 3.52 | 2.63 | 0.23 | 0.0049 | 0.0418 |
| HIST1H2BC | 11.44 | 3.52 | 106.04 | 9.26 | 0.00542 | 0.0418 |
| RIPK4 | 11.53 | 3.53 | 1721.37 | 149.25 | 0.00424 | 0.0418 |
| RHBDL1 | 11.55 | 3.53 | 93.4 | 8.09 | 0.00852 | 0.0464 |
| SMC4 | 11.63 | 3.54 | 4572.71 | 393.02 | 0.00291 | 0.0418 |
| CTAGE9 | 11.65 | 3.54 | 289.63 | 24.85 | 0.00352 | 0.0418 |
| GINS2 | 11.68 | 3.55 | 925.02 | 79.19 | 0.00291 | 0.0418 |
| HIST1H3D | 11.81 | 3.56 | 54.16 | 4.59 | 0.00383 | 0.0418 |
| TRPV4 | 11.8 | 3.56 | 549.96 | 46.61 | 0.00587 | 0.0421 |
| FERMT1 | 11.81 | 3.56 | 1224.51 | 103.65 | 0.00902 | 0.0474 |
| GINS4 | 11.84 | 3.57 | 485.16 | 40.99 | 0.00291 | 0.0418 |
| POLE2 | 11.91 | 3.57 | 294.88 | 24.75 | 0.00291 | 0.0418 |
| PLEKHG6 | 11.9 | 3.57 | 545.78 | 45.87 | 0.0049 | 0.0418 |
| DNA2 | 11.98 | 3.58 | 278.16 | 23.23 | 0.00291 | 0.0418 |
| C14orf182 | 11.99 | 3.58 | 9.51 | 0.79 | 0.00599 | 0.0421 |
| RHBDL2 | 11.96 | 3.58 | 457.14 | 38.22 | 0.00661 | 0.043 |
| SLC22A20 | 12.03 | 3.59 | 34.05 | 2.83 | 0.00937 | 0.0479 |
| ATAD2 | 12.15 | 3.6 | 2268.46 | 186.7 | 0.00291 | 0.0418 |
| ISG15 | 12.09 | 3.6 | 5028.97 | 416.01 | 0.00715 | 0.0437 |
| CENPO | 12.21 | 3.61 | 248.29 | 20.34 | 0.00291 | 0.0418 |
| STYK1 | 12.23 | 3.61 | 255.46 | 20.88 | 0.0048 | 0.0418 |
| F2RL1 | 12.21 | 3.61 | 920.79 | 75.4 | 0.00599 | 0.0421 |
| FANCB | 12.29 | 3.62 | 69.37 | 5.65 | 0.00291 | 0.0418 |
| RAB25 | 12.27 | 3.62 | 3259.84 | 265.67 | 0.00345 | 0.0418 |
| KLF5 | 12.3 | 3.62 | 8608.42 | 699.74 | 0.00383 | 0.0418 |
| PMAIP1 | 12.36 | 3.63 | 820.35 | 66.35 | 0.00317 | 0.0418 |
| ATG9B | 12.4 | 3.63 | 235.62 | 19.01 | 0.00955 | 0.0482 |
| CIP2A | 12.49 | 3.64 | 504.19 | 40.36 | 0.00291 | 0.0418 |
| HIST1H2BG | 12.5 | 3.64 | 31.03 | 2.48 | 0.00828 | 0.046 |
| RACGAP1 | 12.62 | 3.66 | 1663.22 | 131.84 | 0.00291 | 0.0418 |
| C1orf74 | 12.61 | 3.66 | 314.52 | 24.94 | 0.00304 | 0.0418 |
| CTAGE4 | 12.67 | 3.66 | 100.5 | 7.93 | 0.00352 | 0.0418 |
| C2CD4D | 12.63 | 3.66 | 30.28 | 2.4 | 0.00433 | 0.0418 |
| LMNB1 | 12.75 | 3.67 | 2757.25 | 216.28 | 0.00304 | 0.0418 |
| OAS3 | 12.73 | 3.67 | 4158.7 | 326.56 | 0.00345 | 0.0418 |
| ECT2 | 12.85 | 3.68 | 2396.92 | 186.6 | 0.00297 | 0.0418 |
| TMEM79 | 12.79 | 3.68 | 1305.75 | 102.13 | 0.00337 | 0.0418 |
| FAM222A | 12.95 | 3.69 | 152.74 | 11.8 | 0.0031 | 0.0418 |
| CYP2D6 | 12.97 | 3.7 | 55.26 | 4.26 | 0.00759 | 0.0445 |
| ATAD5 | 13.13 | 3.71 | 259.02 | 19.72 | 0.00291 | 0.0418 |
| KIF23 | 13.12 | 3.71 | 1064.23 | 81.1 | 0.00291 | 0.0418 |
| WDR62 | 13.17 | 3.72 | 553.29 | 42.01 | 0.00291 | 0.0418 |
| PIF1 | 13.19 | 3.72 | 260.02 | 19.71 | 0.00297 | 0.0418 |
| MAP3K9 | 13.29 | 3.73 | 159.94 | 12.03 | 0.00317 | 0.0418 |
| HIST1H3H | 13.24 | 3.73 | 39.6 | 2.99 | 0.00345 | 0.0418 |
| KDF1 | 13.32 | 3.74 | 657.66 | 49.39 | 0.00304 | 0.0418 |
| DSG2 | 13.39 | 3.74 | 4361.81 | 325.78 | 0.0031 | 0.0418 |
| OCLN | 13.36 | 3.74 | 304.4 | 22.78 | 0.00433 | 0.0418 |
| MAL2 | 13.52 | 3.76 | 7686.71 | 568.64 | 0.0033 | 0.0418 |
| RMI2 | 13.66 | 3.77 | 1198.01 | 87.69 | 0.00291 | 0.0418 |
| EDARADD | 13.68 | 3.77 | 374.14 | 27.34 | 0.00674 | 0.0432 |
| C17orf53 | 13.71 | 3.78 | 325.44 | 23.73 | 0.00291 | 0.0418 |
| CDH1 | 13.73 | 3.78 | 9346.81 | 680.53 | 0.0031 | 0.0418 |
| CKS2 | 13.83 | 3.79 | 1303.91 | 94.31 | 0.00291 | 0.0418 |
| MCM2 | 13.92 | 3.8 | 5145.41 | 369.59 | 0.00291 | 0.0418 |
| PERP | 13.93 | 3.8 | 32282.76 | 2317.48 | 0.00424 | 0.0418 |
| HIST1H2BJ | 13.9 | 3.8 | 186.94 | 13.45 | 0.00442 | 0.0418 |
| CCNF | 14.11 | 3.82 | 930.08 | 65.93 | 0.00291 | 0.0418 |
| FANCI | 14.16 | 3.82 | 2085.48 | 147.24 | 0.00291 | 0.0418 |
| MAPK13 | 14.11 | 3.82 | 1909.96 | 135.39 | 0.0031 | 0.0418 |
| PLK4 | 14.18 | 3.83 | 487.96 | 34.4 | 0.00291 | 0.0418 |
| GPR19 | 14.27 | 3.83 | 49.53 | 3.47 | 0.0049 | 0.0418 |
| FAM72A | 14.45 | 3.85 | 59.18 | 4.09 | 0.00352 | 0.0418 |
| SLC2A1 | 14.43 | 3.85 | 14994.81 | 1039.48 | 0.0051 | 0.0418 |
| CKMT1B | 14.45 | 3.85 | 2073.51 | 143.52 | 0.0052 | 0.0418 |
| PRC1 | 14.53 | 3.86 | 2540.01 | 174.86 | 0.00291 | 0.0418 |
| ZNF296 | 14.53 | 3.86 | 164.56 | 11.33 | 0.00304 | 0.0418 |
| LTB4R2 | 14.52 | 3.86 | 295.54 | 20.36 | 0.00399 | 0.0418 |
| ARNTL2 | 14.68 | 3.88 | 456.8 | 31.11 | 0.00345 | 0.0418 |
| GRAMD2 | 14.69 | 3.88 | 118.41 | 8.06 | 0.00442 | 0.0418 |
| CENPI | 14.78 | 3.89 | 128.63 | 8.7 | 0.00317 | 0.0418 |
| MOCOS | 14.84 | 3.89 | 518.65 | 34.96 | 0.0051 | 0.0418 |
| TACSTD2 | 14.9 | 3.9 | 25689.4 | 1724.26 | 0.0052 | 0.0418 |
| LOC284837 | 15.11 | 3.92 | 113.14 | 7.49 | 0.00836 | 0.046 |
| RAC3 | 15.24 | 3.93 | 293.64 | 19.27 | 0.00304 | 0.0418 |
| ULBP3 | 15.33 | 3.94 | 60.44 | 3.94 | 0.0052 | 0.0418 |
| ALG1L | 15.38 | 3.94 | 481.28 | 31.3 | 0.0082 | 0.0456 |
| CCNE1 | 15.54 | 3.96 | 390.38 | 25.11 | 0.00291 | 0.0418 |
| RECQL4 | 15.53 | 3.96 | 1096.65 | 70.59 | 0.00291 | 0.0418 |
| CCNA2 | 15.62 | 3.97 | 1191.17 | 76.25 | 0.00291 | 0.0418 |
| PRRG4 | 15.63 | 3.97 | 636.53 | 40.72 | 0.00345 | 0.0418 |
| LAMC2 | 15.69 | 3.97 | 10320.4 | 657.9 | 0.00955 | 0.0482 |
| TTC22 | 15.94 | 3.99 | 279.39 | 17.53 | 0.00564 | 0.0419 |
| TUBBP5 | 15.92 | 3.99 | 137.44 | 8.63 | 0.00974 | 0.0487 |
| PAK6 | 15.97 | 4 | 882.1 | 55.24 | 0.0033 | 0.0418 |
| HPDL | 15.99 | 4 | 182.96 | 11.44 | 0.005 | 0.0418 |
| NUP210 | 16.34 | 4.03 | 3172.46 | 194.14 | 0.00304 | 0.0418 |
| NR6A1 | 16.28 | 4.03 | 14.91 | 0.91 | 0.00359 | 0.0418 |
| GPRIN1 | 16.42 | 4.04 | 392.93 | 23.92 | 0.00304 | 0.0418 |
| IL22RA1 | 16.45 | 4.04 | 262.63 | 15.97 | 0.0048 | 0.0418 |
| PAQR4 | 16.56 | 4.05 | 1706.11 | 102.99 | 0.00291 | 0.0418 |
| IL1RL2 | 16.51 | 4.05 | 67.88 | 4.11 | 0.00661 | 0.043 |
| CCDC150 | 16.84 | 4.07 | 106.55 | 6.32 | 0.00291 | 0.0418 |
| LAD1 | 16.83 | 4.07 | 7978.78 | 473.94 | 0.00391 | 0.0418 |
| TREM2 | 16.75 | 4.07 | 241.41 | 14.41 | 0.00955 | 0.0482 |
| TK1 | 16.97 | 4.09 | 3851.92 | 226.97 | 0.00291 | 0.0418 |
| RAPGEFL1 | 16.98 | 4.09 | 2898.02 | 170.64 | 0.00661 | 0.043 |
| SPAG5 | 17.12 | 4.1 | 1355.89 | 79.21 | 0.00291 | 0.0418 |
| SCD | 17.13 | 4.1 | 10770.1 | 628.77 | 0.0033 | 0.0418 |
| SLC5A10 | 17.18 | 4.1 | 9.25 | 0.54 | 0.00352 | 0.0418 |
| ACBD7 | 17.1 | 4.1 | 130.67 | 7.64 | 0.00611 | 0.0424 |
| EHF | 17.13 | 4.1 | 2863.64 | 167.18 | 0.00937 | 0.0479 |
| FAM72B | 17.43 | 4.12 | 343.59 | 19.71 | 0.00291 | 0.0418 |
| TYMS | 17.38 | 4.12 | 2033.48 | 117 | 0.00291 | 0.0418 |
| CELSR3 | 17.34 | 4.12 | 711.38 | 41.01 | 0.00337 | 0.0418 |
| FBXL16 | 17.46 | 4.13 | 250.14 | 14.32 | 0.00542 | 0.0418 |
| PRR5-ARHGAP8 | 17.48 | 4.13 | 40.74 | 2.33 | 0.00547 | 0.0418 |
| LINC00319 | 17.67 | 4.14 | 113.39 | 6.42 | 0.00542 | 0.0418 |
| RASSF6 | 17.59 | 4.14 | 230.18 | 13.08 | 0.00955 | 0.0482 |
| HK2 | 17.84 | 4.16 | 3095.44 | 173.56 | 0.00337 | 0.0418 |
| CCNB1 | 17.95 | 4.17 | 2330.88 | 129.87 | 0.00291 | 0.0418 |
| EME1 | 17.96 | 4.17 | 199.27 | 11.09 | 0.00291 | 0.0418 |
| MND1 | 17.99 | 4.17 | 176.25 | 9.8 | 0.00297 | 0.0418 |
| PCP2 | 17.95 | 4.17 | 27.78 | 1.55 | 0.00701 | 0.0434 |
| MCM4 | 18.19 | 4.18 | 3727.51 | 204.97 | 0.00291 | 0.0418 |
| E2F1 | 18.21 | 4.19 | 1251.97 | 68.73 | 0.00291 | 0.0418 |
| ACPP | 18.25 | 4.19 | 250.59 | 13.73 | 0.005 | 0.0418 |
| ESRP1 | 18.48 | 4.21 | 2874.62 | 155.58 | 0.00297 | 0.0418 |
| TRIP13 | 18.6 | 4.22 | 839.1 | 45.1 | 0.00291 | 0.0418 |
| CENPM | 18.74 | 4.23 | 589.24 | 31.44 | 0.00291 | 0.0418 |
| RHOV | 19.06 | 4.25 | 3255.63 | 170.85 | 0.00804 | 0.0454 |
| TSPAN10 | 19.26 | 4.27 | 103.53 | 5.37 | 0.00575 | 0.0421 |
| SH3RF2 | 19.4 | 4.28 | 1004.19 | 51.76 | 0.0047 | 0.0418 |
| CTAGE6 | 19.44 | 4.28 | 8.89 | 0.46 | 0.00495 | 0.0418 |
| GINS1 | 19.5 | 4.29 | 901.32 | 46.22 | 0.00291 | 0.0418 |
| MYCL | 19.56 | 4.29 | 616.49 | 31.52 | 0.00323 | 0.0418 |
| CDC25A | 19.69 | 4.3 | 406.21 | 20.63 | 0.00291 | 0.0418 |
| HELLS | 19.65 | 4.3 | 430.02 | 21.88 | 0.00291 | 0.0418 |
| TYMP | 19.8 | 4.31 | 6864.58 | 346.64 | 0.00399 | 0.0418 |
| CENPE | 19.98 | 4.32 | 678.78 | 33.96 | 0.00291 | 0.0418 |
| CYP2J2 | 19.91 | 4.32 | 169.69 | 8.52 | 0.00623 | 0.0425 |
| PRKY | 20.1 | 4.33 | 4.61 | 0.23 | 0.00611 | 0.0424 |
| CNIH2 | 20.21 | 4.34 | 101.65 | 5.03 | 0.00391 | 0.0418 |
| C1orf106 | 20.37 | 4.35 | 2905.26 | 142.6 | 0.00359 | 0.0418 |
| WNT7B | 20.36 | 4.35 | 1165.78 | 57.26 | 0.00416 | 0.0418 |
| TICRR | 20.54 | 4.36 | 543.65 | 26.46 | 0.00291 | 0.0418 |
| UBE2T | 20.64 | 4.37 | 759.32 | 36.78 | 0.00291 | 0.0418 |
| IL23A | 20.62 | 4.37 | 85.56 | 4.15 | 0.00391 | 0.0418 |
| CKMT1A | 20.63 | 4.37 | 793.22 | 38.46 | 0.005 | 0.0418 |
| SOWAHB | 20.73 | 4.37 | 175.21 | 8.45 | 0.00531 | 0.0418 |
| LOC647946 | 20.83 | 4.38 | 54.72 | 2.63 | 0.00796 | 0.0454 |
| RAD51 | 20.95 | 4.39 | 479.67 | 22.9 | 0.00291 | 0.0418 |
| RAD51AP1 | 21.14 | 4.4 | 468.44 | 22.16 | 0.00291 | 0.0418 |
| C12orf70 | 21.24 | 4.41 | 15.56 | 0.73 | 0.005 | 0.0418 |
| ZNF488 | 21.29 | 4.41 | 133.71 | 6.28 | 0.00937 | 0.0479 |
| VGF | 21.34 | 4.42 | 18.67 | 0.87 | 0.00715 | 0.0437 |
| ABCC11 | 21.34 | 4.42 | 4.89 | 0.23 | 0.0078 | 0.0452 |
| TEDC2 | 21.48 | 4.43 | 370.62 | 17.25 | 0.00291 | 0.0418 |
| EVPL | 21.52 | 4.43 | 4388.96 | 203.94 | 0.0031 | 0.0418 |
| ANO9 | 21.57 | 4.43 | 596.14 | 27.63 | 0.00317 | 0.0418 |
| MYB | 21.49 | 4.43 | 288.5 | 13.43 | 0.00352 | 0.0418 |
| GALR2 | 21.71 | 4.44 | 9.93 | 0.46 | 0.00461 | 0.0418 |
| FAM83F | 21.71 | 4.44 | 357.13 | 16.45 | 0.0049 | 0.0418 |
| CDKN3 | 21.88 | 4.45 | 471.55 | 21.55 | 0.00291 | 0.0418 |
| PBK | 21.87 | 4.45 | 556.06 | 25.43 | 0.00291 | 0.0418 |
| POU2F3 | 21.8 | 4.45 | 182.48 | 8.37 | 0.00902 | 0.0474 |
| CDCA3 | 21.97 | 4.46 | 537.77 | 24.48 | 0.00291 | 0.0418 |
| KIFC1 | 22.07 | 4.46 | 1693.75 | 76.75 | 0.00291 | 0.0418 |
| MST1R | 21.99 | 4.46 | 1051.43 | 47.82 | 0.00407 | 0.0418 |
| MYH14 | 22.04 | 4.46 | 8453.4 | 383.46 | 0.00433 | 0.0418 |
| AURKA | 22.12 | 4.47 | 903.76 | 40.86 | 0.00291 | 0.0418 |
| HSPA4L | 22.46 | 4.49 | 573.39 | 25.53 | 0.0052 | 0.0418 |
| ORC6 | 22.84 | 4.51 | 440.24 | 19.27 | 0.00291 | 0.0418 |
| **Gene** | **Fold change** | **log2 (Fold change)** | **Mean TPM* (tumor)** | **Mean TPM* (normal)** | **p-value** | **adjusted p-value** |
| NECTIN1 | 23.02 | 4.53 | 7011.6 | 304.53 | 0.00317 | 0.0418 |
| NECTIN4 | 23.06 | 4.53 | 3810.5 | 165.27 | 0.00375 | 0.0418 |
| LY6K | 23.14 | 4.53 | 1425.47 | 61.59 | 0.00564 | 0.0419 |
| SULT2B1 | 23.07 | 4.53 | 914.94 | 39.66 | 0.00661 | 0.043 |
| MMP9 | 23.02 | 4.53 | 1794.59 | 77.94 | 0.0082 | 0.0456 |
| CDCA5 | 23.39 | 4.55 | 1335.76 | 57.1 | 0.00291 | 0.0418 |
| EPN3 | 23.67 | 4.56 | 973.89 | 41.15 | 0.0033 | 0.0418 |
| BRCA2 | 23.57 | 4.56 | 259.56 | 11.01 | 0.00359 | 0.0418 |
| CYP27B1 | 23.8 | 4.57 | 146.47 | 6.15 | 0.00317 | 0.0418 |
| PKP3 | 23.69 | 4.57 | 4270.33 | 180.26 | 0.0033 | 0.0418 |
| SDC1 | 24.22 | 4.6 | 22519.72 | 929.81 | 0.00337 | 0.0418 |
| XRCC2 | 24.36 | 4.61 | 116.09 | 4.76 | 0.00291 | 0.0418 |
| KLHL35 | 24.44 | 4.61 | 255.66 | 10.46 | 0.005 | 0.0418 |
| EPR1 | 24.38 | 4.61 | 821.13 | 33.68 | 0.0052 | 0.0418 |
| CAMK2N2 | 24.5 | 4.61 | 131.88 | 5.38 | 0.00531 | 0.0418 |
| APOBEC3B | 24.46 | 4.61 | 902.21 | 36.89 | 0.00674 | 0.0432 |
| FAM83H | 24.51 | 4.62 | 4333.36 | 176.83 | 0.00297 | 0.0418 |
| SYCE2 | 24.54 | 4.62 | 87.52 | 3.56 | 0.00531 | 0.0418 |
| DTL | 25.01 | 4.64 | 1039.3 | 41.56 | 0.00291 | 0.0418 |
| PSAT1 | 25.19 | 4.66 | 1213.81 | 48.18 | 0.0033 | 0.0418 |
| KRT86 | 25.54 | 4.67 | 94.67 | 3.71 | 0.0051 | 0.0418 |
| CDK1 | 25.66 | 4.68 | 2117.13 | 82.49 | 0.00291 | 0.0418 |
| C6orf105 | 25.56 | 4.68 | 66.2 | 2.59 | 0.00836 | 0.046 |
| LIPG | 25.68 | 4.68 | 267.15 | 10.4 | 0.00836 | 0.046 |
| BICDL1 | 25.89 | 4.69 | 757.72 | 29.27 | 0.00297 | 0.0418 |
| CCNE2 | 25.92 | 4.7 | 560.69 | 21.63 | 0.00297 | 0.0418 |
| NETO2 | 26.41 | 4.72 | 925.07 | 35.03 | 0.00323 | 0.0418 |
| APOC1 | 26.6 | 4.73 | 1272.31 | 47.82 | 0.00461 | 0.0418 |
| ZWINT | 26.99 | 4.75 | 1802.46 | 66.78 | 0.00304 | 0.0418 |
| STRIP2 | 27.25 | 4.77 | 282.23 | 10.36 | 0.00304 | 0.0418 |
| HASPIN | 27.51 | 4.78 | 143.57 | 5.22 | 0.00291 | 0.0418 |
| STIL | 27.39 | 4.78 | 703.3 | 25.67 | 0.00291 | 0.0418 |
| TDO2 | 27.83 | 4.8 | 47.74 | 1.71 | 0.00902 | 0.0474 |
| EPHA1 | 28 | 4.81 | 821.68 | 29.34 | 0.00291 | 0.0418 |
| EZH2 | 28.47 | 4.83 | 1112.53 | 39.08 | 0.00291 | 0.0418 |
| KIF11 | 28.43 | 4.83 | 1545.79 | 54.36 | 0.00291 | 0.0418 |
| GRHL2 | 28.64 | 4.84 | 1665.68 | 58.16 | 0.00297 | 0.0418 |
| NIPAL1 | 28.66 | 4.84 | 52.84 | 1.84 | 0.00715 | 0.0437 |
| ULBP1 | 28.66 | 4.84 | 70.44 | 2.46 | 0.00715 | 0.0437 |
| NKPD1 | 28.91 | 4.85 | 63.87 | 2.21 | 0.00461 | 0.0418 |
| CPNE7 | 28.81 | 4.85 | 183.86 | 6.38 | 0.005 | 0.0418 |
| B3GNT4 | 29.19 | 4.87 | 45.1 | 1.54 | 0.00323 | 0.0418 |
| ARHGAP11A | 29.52 | 4.88 | 936.65 | 31.73 | 0.00291 | 0.0418 |
| FANCA | 29.5 | 4.88 | 1001.71 | 33.95 | 0.00291 | 0.0418 |
| IRF6 | 29.6 | 4.89 | 3065.5 | 103.55 | 0.0031 | 0.0418 |
| SHCBP1 | 29.78 | 4.9 | 492 | 16.52 | 0.00291 | 0.0418 |
| GJB3 | 29.86 | 4.9 | 2290.17 | 76.71 | 0.00442 | 0.0418 |
| DUOXA1 | 29.84 | 4.9 | 639.78 | 21.44 | 0.00759 | 0.0445 |
| LARGE2 | 30.27 | 4.92 | 565.83 | 18.69 | 0.00383 | 0.0418 |
| LAMP3 | 30.35 | 4.92 | 1290.93 | 42.53 | 0.00391 | 0.0418 |
| PLCH2 | 30.17 | 4.92 | 1102.85 | 36.55 | 0.00992 | 0.0488 |
| FAM57B | 30.42 | 4.93 | 9.37 | 0.31 | 0.00515 | 0.0418 |
| TNS4 | 30.44 | 4.93 | 4530.32 | 148.81 | 0.00688 | 0.0433 |
| SAPCD2 | 30.75 | 4.94 | 1042.66 | 33.91 | 0.00297 | 0.0418 |
| KIF4B | 30.92 | 4.95 | 14.06 | 0.45 | 0.00291 | 0.0418 |
| CDC42BPG | 31.16 | 4.96 | 927.9 | 29.78 | 0.00291 | 0.0418 |
| PLK1 | 31.21 | 4.96 | 1392.77 | 44.62 | 0.00291 | 0.0418 |
| SPC25 | 31.18 | 4.96 | 272.61 | 8.74 | 0.00291 | 0.0418 |
| CTSV | 31.15 | 4.96 | 1466.61 | 47.09 | 0.00304 | 0.0418 |
| RTKN2 | 31.17 | 4.96 | 491.38 | 15.76 | 0.00317 | 0.0418 |
| FOXH1 | 31.09 | 4.96 | 9.07 | 0.29 | 0.00356 | 0.0418 |
| BIRC5 | 31.33 | 4.97 | 1016.15 | 32.43 | 0.00291 | 0.0418 |
| RASSF10 | 31.75 | 4.99 | 197.79 | 6.23 | 0.00587 | 0.0421 |
| AQP3 | 32.21 | 5.01 | 18567.29 | 576.48 | 0.00974 | 0.0487 |
| AUNIP | 32.43 | 5.02 | 223.04 | 6.88 | 0.00291 | 0.0418 |
| MESP2 | 32.39 | 5.02 | 68.84 | 2.12 | 0.00701 | 0.0434 |
| CENPK | 32.69 | 5.03 | 409.78 | 12.53 | 0.00291 | 0.0418 |
| DIAPH3 | 32.92 | 5.04 | 354.15 | 10.76 | 0.00297 | 0.0418 |
| LOC541473 | 32.91 | 5.04 | 50.15 | 1.52 | 0.00515 | 0.0418 |
| COX6B2 | 33.09 | 5.05 | 183.25 | 5.54 | 0.00688 | 0.0433 |
| MICALCL | 33.5 | 5.07 | 46 | 1.37 | 0.00515 | 0.0418 |
| CDC25C | 33.85 | 5.08 | 263.3 | 7.78 | 0.00291 | 0.0418 |
| CDT1 | 34.34 | 5.1 | 1469.4 | 42.79 | 0.00291 | 0.0418 |
| PTTG1 | 34.69 | 5.12 | 1947.94 | 56.15 | 0.00291 | 0.0418 |
| RAD54L | 35.13 | 5.13 | 588.39 | 16.75 | 0.00291 | 0.0418 |
| ERCC6L | 35.01 | 5.13 | 259.71 | 7.42 | 0.00359 | 0.0418 |
| PCLAF | 35.62 | 5.15 | 1291.83 | 36.27 | 0.00297 | 0.0418 |
| RAB26 | 35.58 | 5.15 | 129.05 | 3.63 | 0.00416 | 0.0418 |
| RAB27B | 35.5 | 5.15 | 139.14 | 3.92 | 0.005 | 0.0418 |
| OASL | 35.84 | 5.16 | 824.33 | 23 | 0.00416 | 0.0418 |
| KRT17 | 35.73 | 5.16 | 104474.25 | 2924.33 | 0.00701 | 0.0434 |
| MTFR2 | 36.07 | 5.17 | 167.29 | 4.64 | 0.00291 | 0.0418 |
| SYNGR3 | 36.2 | 5.18 | 300.36 | 8.3 | 0.00407 | 0.0418 |
| SPC24 | 36.83 | 5.2 | 70.85 | 1.92 | 0.00304 | 0.0418 |
| C20orf151 | 36.7 | 5.2 | 196.25 | 5.35 | 0.00367 | 0.0418 |
| S100A14 | 37 | 5.21 | 11799.33 | 318.94 | 0.00433 | 0.0418 |
| GRHL1 | 37.36 | 5.22 | 1609.41 | 43.08 | 0.00352 | 0.0418 |
| ALDH3B2 | 37.3 | 5.22 | 1128.04 | 30.24 | 0.00575 | 0.0421 |
| ASF1B | 37.6 | 5.23 | 1796.82 | 47.79 | 0.00291 | 0.0418 |
| KIF2C | 37.52 | 5.23 | 1538.46 | 41.01 | 0.00291 | 0.0418 |
| SLC27A2 | 37.79 | 5.24 | 194.67 | 5.15 | 0.0049 | 0.0418 |
| MT1G | 38.41 | 5.26 | 272.15 | 7.08 | 0.0051 | 0.0418 |
| CENPF | 38.73 | 5.28 | 2942.84 | 75.99 | 0.00291 | 0.0418 |
| FAM72D | 39.03 | 5.29 | 164.78 | 4.22 | 0.00291 | 0.0418 |
| PRR11 | 39.24 | 5.29 | 156.78 | 3.99 | 0.00553 | 0.0418 |
| ORC1 | 39.44 | 5.3 | 501.45 | 12.71 | 0.00291 | 0.0418 |
| HIST1H2AM | 39.56 | 5.31 | 13.53 | 0.34 | 0.00291 | 0.0418 |
| NUSAP1 | 39.55 | 5.31 | 2324.76 | 58.79 | 0.00291 | 0.0418 |
| SEPT3 | 39.76 | 5.31 | 296.96 | 7.47 | 0.0033 | 0.0418 |
| FAM64A | 39.96 | 5.32 | 310.78 | 7.78 | 0.00291 | 0.0418 |
| ITGB6 | 39.87 | 5.32 | 3156.41 | 79.16 | 0.00416 | 0.0418 |
| TMEM171 | 40.17 | 5.33 | 144.38 | 3.59 | 0.00407 | 0.0418 |
| CDCA8 | 40.54 | 5.34 | 1008.31 | 24.87 | 0.00291 | 0.0418 |
| CENPU | 40.39 | 5.34 | 1203.6 | 29.8 | 0.00291 | 0.0418 |
| KIF18A | 40.85 | 5.35 | 308 | 7.54 | 0.00291 | 0.0418 |
| DSP | 40.68 | 5.35 | 27101.37 | 666.18 | 0.00297 | 0.0418 |
| HCAR2 | 41.24 | 5.37 | 952.69 | 23.1 | 0.00974 | 0.0487 |
| DEPDC1B | 41.61 | 5.38 | 351.79 | 8.45 | 0.00291 | 0.0418 |
| TRIM29 | 41.72 | 5.38 | 13546.54 | 324.7 | 0.00636 | 0.0427 |
| RGS20 | 41.69 | 5.38 | 65.4 | 1.57 | 0.00868 | 0.0467 |
| DLX4 | 42.22 | 5.4 | 70.45 | 1.67 | 0.00407 | 0.0418 |
| F12 | 42.41 | 5.41 | 346.48 | 8.17 | 0.00323 | 0.0418 |
| OVOL1 | 42.72 | 5.42 | 880.12 | 20.6 | 0.00337 | 0.0418 |
| GPRIN2 | 42.86 | 5.42 | 189.8 | 4.43 | 0.00547 | 0.0418 |
| KIF15 | 42.98 | 5.43 | 487.22 | 11.34 | 0.00291 | 0.0418 |
| IGLON5 | 43.47 | 5.44 | 18.4 | 0.42 | 0.00433 | 0.0418 |
| THEM5 | 43.41 | 5.44 | 31.58 | 0.73 | 0.00708 | 0.0437 |
| BUB1 | 43.84 | 5.45 | 806.42 | 18.4 | 0.00291 | 0.0418 |
| UBE2C | 44.02 | 5.46 | 1892.68 | 42.99 | 0.00291 | 0.0418 |
| GRIN1 | 43.91 | 5.46 | 15.02 | 0.34 | 0.00515 | 0.0418 |
| BLM | 44.25 | 5.47 | 364.72 | 8.24 | 0.00291 | 0.0418 |
| NCAPG | 44.27 | 5.47 | 919.24 | 20.76 | 0.00291 | 0.0418 |
| CARD14 | 44.97 | 5.49 | 323.28 | 7.19 | 0.00399 | 0.0418 |
| MKI67 | 45.3 | 5.5 | 4453.7 | 98.31 | 0.00291 | 0.0418 |
| C12orf59 | 45.68 | 5.51 | 47.37 | 1.04 | 0.0073 | 0.0439 |
| TTK | 46.28 | 5.53 | 615.1 | 13.29 | 0.00291 | 0.0418 |
| LOC100132111 | 46.98 | 5.55 | 29.11 | 0.62 | 0.0057 | 0.0421 |
| CLDN14 | 47.21 | 5.56 | 29.14 | 0.62 | 0.00701 | 0.0434 |
| CCNB2 | 47.68 | 5.58 | 1275.39 | 26.75 | 0.00297 | 0.0418 |
| ULBP2 | 47.75 | 5.58 | 330.68 | 6.92 | 0.00407 | 0.0418 |
| LOC399815 | 48.28 | 5.59 | 27.53 | 0.57 | 0.00461 | 0.0418 |
| PAX9 | 48.02 | 5.59 | 440.64 | 9.18 | 0.00744 | 0.0442 |
| IRX5 | 48.14 | 5.59 | 310.59 | 6.45 | 0.00759 | 0.0445 |
| IGSF9 | 49.15 | 5.62 | 1339.97 | 27.26 | 0.00297 | 0.0418 |
| C9orf169 | 49.64 | 5.63 | 237.87 | 4.79 | 0.0073 | 0.0439 |
| DDN | 49.77 | 5.64 | 22.76 | 0.46 | 0.00442 | 0.0418 |
| AMH | 50.34 | 5.65 | 62.05 | 1.23 | 0.00902 | 0.0474 |
| CEP55 | 51.26 | 5.68 | 1198.83 | 23.39 | 0.00291 | 0.0418 |
| PKMYT1 | 51.8 | 5.69 | 1161.8 | 22.43 | 0.00291 | 0.0418 |
| ADAMDEC1 | 51.47 | 5.69 | 165.26 | 3.21 | 0.00836 | 0.046 |
| BRIP1 | 52.72 | 5.72 | 261.16 | 4.95 | 0.00291 | 0.0418 |
| CENPA | 52.96 | 5.73 | 395.52 | 7.47 | 0.00291 | 0.0418 |
| DEPDC1 | 53.93 | 5.75 | 572.72 | 10.62 | 0.00291 | 0.0418 |
| SALL4 | 54.23 | 5.76 | 76.97 | 1.42 | 0.0051 | 0.0418 |
| MT1H | 54.09 | 5.76 | 33.25 | 0.61 | 0.00526 | 0.0418 |
| ISL2 | 54.72 | 5.77 | 48.7 | 0.89 | 0.00885 | 0.0471 |
| RDM1 | 54.92 | 5.78 | 38.23 | 0.7 | 0.00337 | 0.0418 |
| CDC6 | 55.38 | 5.79 | 1129.12 | 20.39 | 0.00291 | 0.0418 |
| KIF4A | 55.5 | 5.79 | 1024.28 | 18.45 | 0.00291 | 0.0418 |
| GJB4 | 55.3 | 5.79 | 329.84 | 5.96 | 0.00636 | 0.0427 |
| AURKB | 55.54 | 5.8 | 1014.4 | 18.26 | 0.00291 | 0.0418 |
| MELK | 55.65 | 5.8 | 963.41 | 17.31 | 0.00291 | 0.0418 |
| HMMR | 56.41 | 5.82 | 556.97 | 9.87 | 0.00291 | 0.0418 |
| KIF14 | 56.69 | 5.83 | 430.76 | 7.6 | 0.00291 | 0.0418 |
| PLEKHN1 | 56.81 | 5.83 | 223.81 | 3.94 | 0.00367 | 0.0418 |
| CDRT1 | 57.16 | 5.84 | 17.74 | 0.31 | 0.00642 | 0.043 |
| FAM83B | 57.66 | 5.85 | 355.57 | 6.17 | 0.00424 | 0.0418 |
| SPTSSB | 57.92 | 5.86 | 366.97 | 6.34 | 0.0092 | 0.0476 |
| CDC20 | 58.65 | 5.87 | 2347.04 | 40.02 | 0.00291 | 0.0418 |
| RRM2 | 58.48 | 5.87 | 3436.17 | 58.76 | 0.00291 | 0.0418 |
| TOP2A | 58.49 | 5.87 | 4932.33 | 84.33 | 0.00291 | 0.0418 |
| S100A2 | 58.58 | 5.87 | 28601.64 | 488.21 | 0.00789 | 0.0452 |
| FOXM1 | 58.91 | 5.88 | 2182.32 | 37.05 | 0.00291 | 0.0418 |
| NCAPH | 58.98 | 5.88 | 662.56 | 11.23 | 0.00291 | 0.0418 |
| GRIN2D | 59.45 | 5.89 | 373.85 | 6.29 | 0.00345 | 0.0418 |
| IGF2BP3 | 59.45 | 5.89 | 455 | 7.65 | 0.00992 | 0.0488 |
| ESCO2 | 59.72 | 5.9 | 301.7 | 5.05 | 0.00291 | 0.0418 |
| TPX2 | 59.98 | 5.91 | 3173.17 | 52.9 | 0.00291 | 0.0418 |
| SKA1 | 60.37 | 5.92 | 407.72 | 6.75 | 0.00291 | 0.0418 |
| KRT80 | 60.72 | 5.92 | 2194.15 | 36.13 | 0.0033 | 0.0418 |
| MMP12 | 60.64 | 5.92 | 739.72 | 12.2 | 0.00623 | 0.0425 |
| CDCA2 | 62.01 | 5.95 | 399.11 | 6.44 | 0.00291 | 0.0418 |
| FAM111B | 61.63 | 5.95 | 919.82 | 14.92 | 0.00291 | 0.0418 |
| EXO1 | 62.2 | 5.96 | 459.21 | 7.38 | 0.00291 | 0.0418 |
| SKA3 | 62.28 | 5.96 | 397.02 | 6.37 | 0.00291 | 0.0418 |
| **Gene** | **Fold change** | **log2 (Fold change)** | **Mean TPM* (tumor)** | **Mean TPM* (normal)** | **p-value** | **adjusted p-value** |
| TNNT1 | 62.23 | 5.96 | 999.38 | 16.06 | 0.00674 | 0.0432 |
| IQGAP3 | 62.95 | 5.98 | 1604.7 | 25.49 | 0.00291 | 0.0418 |
| ANLN | 63.8 | 6 | 2057.25 | 32.24 | 0.00291 | 0.0418 |
| OIP5 | 64.59 | 6.01 | 270.92 | 4.19 | 0.00291 | 0.0418 |
| SGO1 | 65.01 | 6.02 | 170.4 | 2.62 | 0.00291 | 0.0418 |
| ESPL1 | 65.45 | 6.03 | 795.64 | 12.16 | 0.00291 | 0.0418 |
| ARHGAP11B | 65.89 | 6.04 | 67.31 | 1.02 | 0.00387 | 0.0418 |
| LYPD3 | 65.96 | 6.04 | 8448.51 | 128.08 | 0.00937 | 0.0479 |
| KLC3 | 66.36 | 6.05 | 453.94 | 6.84 | 0.00416 | 0.0418 |
| BUB1B | 68.82 | 6.1 | 838.71 | 12.19 | 0.00291 | 0.0418 |
| CXCL10 | 68.68 | 6.1 | 2395.14 | 34.87 | 0.00804 | 0.0454 |
| HMSD | 68.82 | 6.1 | 42.31 | 0.61 | 0.00885 | 0.0471 |
| GTSE1 | 69.39 | 6.12 | 582.52 | 8.39 | 0.00304 | 0.0418 |
| FAM83D | 70.93 | 6.15 | 1147.74 | 16.18 | 0.00291 | 0.0418 |
| HJURP | 71.81 | 6.17 | 625.71 | 8.71 | 0.00291 | 0.0418 |
| DSC2 | 72.54 | 6.18 | 5286.77 | 72.88 | 0.00337 | 0.0418 |
| CDKN2BAS | 72.5 | 6.18 | 74.93 | 1.03 | 0.00399 | 0.0418 |
| RUFY4 | 73.47 | 6.2 | 41.71 | 0.57 | 0.00451 | 0.0418 |
| PLA2G4F | 76.72 | 6.26 | 202.48 | 2.64 | 0.00581 | 0.0421 |
| CEL | 77.93 | 6.28 | 982.29 | 12.6 | 0.00744 | 0.0442 |
| POLQ | 79.6 | 6.31 | 541.48 | 6.8 | 0.00291 | 0.0418 |
| ASPM | 79.88 | 6.32 | 1259.25 | 15.76 | 0.00291 | 0.0418 |
| CKAP2L | 79.71 | 6.32 | 333.11 | 4.18 | 0.00291 | 0.0418 |
| KIF20A | 82.26 | 6.36 | 1209.46 | 14.7 | 0.00291 | 0.0418 |
| TP73 | 82.38 | 6.36 | 318.77 | 3.87 | 0.00323 | 0.0418 |
| KLHDC7B | 82.1 | 6.36 | 2675.8 | 32.59 | 0.00868 | 0.0467 |
| E2F2 | 83.72 | 6.39 | 711.49 | 8.5 | 0.00291 | 0.0418 |
| HES2 | 85.19 | 6.41 | 1868.36 | 21.93 | 0.00611 | 0.0424 |
| TROAP | 85.34 | 6.42 | 850.57 | 9.97 | 0.00291 | 0.0418 |
| GUCY1B2 | 86.32 | 6.43 | 19.78 | 0.23 | 0.00758 | 0.0445 |
| NUF2 | 86.57 | 6.44 | 658.22 | 7.6 | 0.00291 | 0.0418 |
| PLEK2 | 86.85 | 6.44 | 987.65 | 11.37 | 0.00297 | 0.0418 |
| NEK2 | 88.34 | 6.47 | 807.01 | 9.13 | 0.00291 | 0.0418 |
| KCNS1 | 88.64 | 6.47 | 542.88 | 6.12 | 0.00992 | 0.0488 |
| TFAP2A | 89.28 | 6.48 | 1502.99 | 16.83 | 0.00345 | 0.0418 |
| EVPLL | 89.66 | 6.49 | 62.63 | 0.7 | 0.0052 | 0.0418 |
| TFR2 | 91.78 | 6.52 | 138.27 | 1.51 | 0.00317 | 0.0418 |
| CA2 | 91.78 | 6.52 | 1525.83 | 16.62 | 0.00359 | 0.0418 |
| LOC254559 | 91.58 | 6.52 | 918.65 | 10.03 | 0.00553 | 0.0418 |
| TLX2 | 91.55 | 6.52 | 20.98 | 0.23 | 0.00661 | 0.043 |
| KIF18B | 92.3 | 6.53 | 789.82 | 8.56 | 0.00291 | 0.0418 |
| DLGAP5 | 95.85 | 6.58 | 843.51 | 8.8 | 0.00291 | 0.0418 |
| UHRF1 | 97.98 | 6.61 | 1222.46 | 12.48 | 0.00291 | 0.0418 |
| CDC45 | 98.72 | 6.63 | 722.2 | 7.31 | 0.00291 | 0.0418 |
| SYT8 | 100.37 | 6.65 | 849.06 | 8.46 | 0.00955 | 0.0482 |
| E2F8 | 102.09 | 6.67 | 460.8 | 4.51 | 0.00291 | 0.0418 |
| MYBL2 | 102.75 | 6.68 | 3218.45 | 31.32 | 0.00291 | 0.0418 |
| MCM10 | 105.62 | 6.72 | 439.46 | 4.16 | 0.00291 | 0.0418 |
| PPP1R14C | 105.68 | 6.72 | 686.35 | 6.49 | 0.00433 | 0.0418 |
| S100A9 | 109.35 | 6.77 | 67240.3 | 614.93 | 0.00992 | 0.0488 |
| RASEF | 109.79 | 6.78 | 138.52 | 1.26 | 0.00581 | 0.0421 |
| DMBX1 | 110.7 | 6.79 | 77.06 | 0.7 | 0.00655 | 0.043 |
| LOC642587 | 113.18 | 6.82 | 2555.16 | 22.57 | 0.00937 | 0.0479 |
| GJB5 | 119.45 | 6.9 | 996.64 | 8.34 | 0.0051 | 0.0418 |
| CDKN2A | 121.04 | 6.92 | 3850.07 | 31.81 | 0.00359 | 0.0418 |
| CA9 | 121.92 | 6.93 | 1778.07 | 14.58 | 0.00674 | 0.0432 |
| NXPH4 | 132.35 | 7.05 | 694.35 | 5.25 | 0.00345 | 0.0418 |
| NEIL3 | 134.27 | 7.07 | 136.83 | 1.02 | 0.00304 | 0.0418 |
| IL20RB | 138.2 | 7.11 | 2790.77 | 20.19 | 0.00852 | 0.0464 |
| GPR87 | 149.18 | 7.22 | 1544.35 | 10.35 | 0.0082 | 0.0456 |
| CCL18 | 150.73 | 7.24 | 418.33 | 2.77 | 0.00416 | 0.0418 |
| KNL1 | 151.81 | 7.25 | 344.54 | 2.27 | 0.00291 | 0.0418 |
| TM4SF19 | 152.22 | 7.25 | 94.18 | 0.62 | 0.0049 | 0.0418 |
| AIM2 | 151.75 | 7.25 | 583.92 | 3.85 | 0.00868 | 0.0467 |
| SERPINB5 | 157.97 | 7.3 | 6131.66 | 38.81 | 0.00688 | 0.0433 |
| HOXC13 | 166.75 | 7.38 | 148.43 | 0.89 | 0.00955 | 0.0482 |
| LOC100131726 | 174.36 | 7.45 | 54.11 | 0.31 | 0.00722 | 0.0439 |
| BNIPL | 176.4 | 7.46 | 479.21 | 2.72 | 0.0048 | 0.0418 |
| MNX1 | 178.45 | 7.48 | 40.89 | 0.23 | 0.00352 | 0.0418 |
| SFN | 184.1 | 7.52 | 41560.92 | 225.75 | 0.0031 | 0.0418 |
| CLSPN | 186.23 | 7.54 | 314.24 | 1.69 | 0.00291 | 0.0418 |
| CRYBG2 | 186.66 | 7.54 | 809.86 | 4.34 | 0.0033 | 0.0418 |
| E2F7 | 196.31 | 7.62 | 556.58 | 2.83 | 0.00291 | 0.0418 |
| FAM83A | 199.6 | 7.64 | 2972.89 | 14.89 | 0.00542 | 0.0418 |
| FOXE1 | 202.64 | 7.66 | 602.93 | 2.97 | 0.00911 | 0.0476 |
| SMC1B | 215.67 | 7.75 | 365.47 | 1.69 | 0.00674 | 0.0432 |
| TP53AIP1 | 217.06 | 7.76 | 116.36 | 0.54 | 0.00531 | 0.0418 |
| KRT15 | 220.58 | 7.79 | 25763.78 | 116.8 | 0.0073 | 0.0439 |
| ANKRD34B | 222.08 | 7.79 | 68.92 | 0.31 | 0.00991 | 0.0488 |
| ANXA8 | 235.58 | 7.88 | 6287.04 | 26.69 | 0.0092 | 0.0476 |
| FOXA1 | 240.81 | 7.91 | 942.54 | 3.91 | 0.00352 | 0.0418 |
| ANXA8L2 | 242.66 | 7.92 | 2113.7 | 8.71 | 0.0082 | 0.0456 |
| SERPINB2 | 256.7 | 8 | 1150.23 | 4.48 | 0.00868 | 0.0467 |
| DUSP9 | 257.83 | 8.01 | 180.1 | 0.7 | 0.005 | 0.0418 |
| GRHL3 | 279.41 | 8.13 | 1013.72 | 3.63 | 0.00367 | 0.0418 |
| DMRTA2 | 301.68 | 8.24 | 151.43 | 0.5 | 0.00766 | 0.0448 |
| KLRG2 | 311.11 | 8.28 | 71.28 | 0.23 | 0.0048 | 0.0418 |
| PITX1 | 312.21 | 8.29 | 5026.99 | 16.1 | 0.00297 | 0.0418 |
| KRT78 | 312.01 | 8.29 | 217.18 | 0.7 | 0.00593 | 0.0421 |
| ABCA12 | 315.37 | 8.3 | 377.52 | 1.2 | 0.00403 | 0.0418 |
| KREMEN2 | 373.78 | 8.55 | 304.17 | 0.81 | 0.00291 | 0.0418 |
| GSDMC | 455.04 | 8.83 | 611.57 | 1.34 | 0.00359 | 0.0418 |
| ZIC2 | 588.03 | 9.2 | 335.23 | 0.57 | 0.00363 | 0.0418 |
| SOX21 | 644.53 | 9.33 | 272.76 | 0.42 | 0.00955 | 0.0482 |
| LGALS7 | 654.79 | 9.35 | 5137.29 | 7.84 | 0.00974 | 0.0487 |
| KRT81 | 1007.41 | 9.98 | 2140.89 | 2.12 | 0.0033 | 0.0418 |
| FAM83C | 1996.51 | 10.96 | 619.62 | 0.31 | 0.00617 | 0.0425 |
