# Supplementary material for: Analysis of miR-497/195 cluster identifies new therapeutic targets in cervical cancer
Source: BMC Res Notes. 2024 Aug 2;17:217. doi: 10.1186/s13104-024-06876-8 (PMC11297691; doi:10.1186/s13104-024-06876-8)
Supplement: Supplementary file 12 — Additional file 12: Table 3. List of genes targeted by miRNA-497/195 cluster. [file 13104_2024_6876_MOESM12_ESM.docx]

**Supplementary Table 3: List of genes targeted by miRNA-497/195 MC.**

| **Gene Numbers** | **Genes** |
| --- | --- |
| **miRNA-497/195 cluster targeted genes**  **(Total -646)** | *DNAJA1, FASN, BZW1, USP15, AGO2, RNF149, JARID2, TPM3, CYP26B1, GATAD2A, EFTUD2, YRDC, CDK6, VEGFA, BCL2L12, C16orf72, CCND2, SOWAHC, FBXL18, UBE2V1, LUZP1, BAZ2A, SLC2A3, ABL2, PAG1, NAA25, TFAP2A, EIF2B2, DIAPH1, PPIL1, NR6A1, HDGF, ZNF622, ACTR2, CHAC, HSPA8, CD274, AVL9, MAFK, PLAG1, TUBB2A, L2HGDH, IER2, GPRC5A, RCAN3, N4BP1, RAD23B, FOXK1, CCND1, AHNAK2, SIK1, E2F3, IPPK, MYO5A, FURIN, RTN4, TNFSF9, CACUL1, PRDM4, KPNA3, OSCAR, TSC22D2, CSNK1E, CBX4, CDCA4, RIMS3, VPS4A, CARM1, PDIA6, PAFAH1B2, PLEKHB2, TAF13, NCKAP1, ZNF460, NAPG, PIM1, SBNO1, HIGD1A, FCF1, HSPA1B, YWHAQ, ARHGDIA, CCNE1, AMOTL1, CRKL, AP5Z1, PPP2R5C, RPS6KB1, SNRPB2, RAB3IP, SEC24A, MOB4, PPP1R11, SSRP1, MCFD2, CCND3, HSPA4L, KIF23, ATP5G3, RUBCN, OSBPL3, TMEM189, ZNRF2, CANX, EFNB2, CALU, SKI, NUP50, CLIP4, C11orf24, WIPI2, RPL14, GNB1, CCNT1, ADORA3, GABARAPL1, AURKAIP1, FGFR4, EXT1, FBXL20, APP, BAG4, GRB2, CDC25A, REL, ZNF391, ORC4, PRKAR2A, CLEC2D, TRIM35, BTRC, CLUH, MINK1, RACGAP1, ARIH1, USP42, MAP3K7, TM7SF3, KPNA1, SERBP1, CMTM4, HIST2H2BE, NUFIP2, PDCD1, RIF1, ZBTB34, TMEM245, CD180, GPR180, HOXA3, TBPL1, CRIM1, HNRNPA2B1, TARBP2, ASCC1, CLSPN, DDX3X, MAP2K3, SNCG, MSANTD4, TLL1, UBE2Q1, YIPF6, FZD9, BIRC5, UBE2H, DYNLL2, ITGA2, SLCO3A1, DICER1, HNRNPA1L2, TAOK1, CAMSAP1, CRK, SLC29A1, ALDH3B1, FAM103A1, LAMC1, CASKIN1, RPRD1B, AKR1B10, CEP55, CD2AP, MTHFR, DCTN5, GALNT1, ZNF284, SZRD1, JPT2, SMAD7, PAK2, IVNS1ABP, KIF5B, PHKA1, PLRG1, SRSF1, DCAF17, STRADB, LRIG2, PHLPP2, DMPK, FZD6, ZDHHC16, KIAA0895, KMT2D, CBX2, LAMP2, PNPLA6, RUNX2, SETD1B, RASEF, GPATCH8, PISD, SEC61A1, SOCS5, SRPRA, TLK1, NOTCH2, HNRNPA1, SLC39A9, TOB2, CDK4, DNAJC10, ODF2L, SNTB2, ATG14, CDK17, EIF1AX, PSAT1, UBR3, CHEK1, SHOC2, AP2B1, VAV2, MAP4K2, ZNRF3, FKBP1A, HOXC8, CARD10, SMAD3, GNG12, TMEM161B, RAPH1, CDC37L1, CDC42SE2, PLEKHA1, PDE4D, RNF168, GPR27, RS1, ARHGAP32, ASH1L, SMURF1, YWHAH, ZBTB10, ATAD5, LRRFIP2, SSU72, RNPS1, DYRK3, ZNF585B, WNT7A, B3GNT2, LSM11, POM121C, SYNRG, TM4SF1, ATG9A, RFK, MKX, MSL1, BTN3A3, USP48, AKT3, CBX6, GRAMD2B, CAPZA2, BSPRY, VSIR, MTMR3, ZMAT3, C1orf226, KANK1, ATXN7L3B, GOSR1, RALGAPB, RECK, STK38, ZNF267, CPSF7, PHF19, ZCCHC3, E2F7, ELK4, HAUS3, PPP6R3, GABPA, RPRD2, CCNE2, PPIG, SRPRB, TTLL5, DLGAP3, DOCK11, OCRL, MBD4, CDK1, FLCN, NFIC, CUL3, KLHL15, RAF1, CHMP4B, HOXA10, ZNF691, ENTPD1, PPM1A, PLPBP, ZFHX4, SNX16, CREBL2, CTDSPL, USP53, PRSS21, RBBP6, ZNF449, GNAL, EN2, VOPP1, TPM2, RAB23, HNRNPDL, THRAP3, TBRG1, TXNIP, PI4K2B, UBN2, YTHDC1, CASK, SESTD1, CDADC1, ZNF367, CPEB3, ACVR2A, DMRT2, CREBRF, POLDIP3, SLC25A12, AGO4, SIDT2, RAB15, ABHD2, HCFC2, OGT, ASGR2, PAGR1, CCDC88C, POLR2E, PRKAA1, WEE1, KIAA1456, ANKRD36, RASSF2, PPIP5K2, SYPL1, ARHGAP12, ZBTB16, CLU, PTPRD, RUNX1T1, LANCL1, C1orf21, LUC7L3, SH3BP4, WDR13, PNRC2, DECR1, ZNF704, C21orf62, SLC35E2B, PNISR, ZNF620, C3orf36, CNKSR3, SREK1, TNRC6B, WNK3, ADRA2B, ANAPC13, SPRED1, ACTR3B, CA8, ARMC12, NNT, CCDC80, FGF2, FLOT2, ZFP28, PHC3, PIK3R1, ZNRF1, AMOT, C16orf58, FAM122B, SCAMP4, DMTF1, EPM2AIP1, HEYL, PHYHIP, RBPJ, TRAK1, ZNF275, ABCC6, NUCKS1, PRICKLE2, LURAP1L, BCL2, DENND6A, FAM229B, CHIC1, SMDT1, MTFR1L, AXIN2, SALL1, NEGR1, PLPP3, RARB, TMEM100, TGFBR3, ANKMY1, MAP2K1, ANLN, IGF1R, ESRRA, KCNN4, HIST1H3H, TCEA1, EEF1A1, MTOR, TWIST1, MACC1, BRD1, DESI2, TPM1, EIF4E, PANK2, PBX3, IKBKB, ALDH9A1, DNAJA1, FASN, BZW1, USP15, AGO2, RNF149, JARID2, TPM3, CYP26B1, GATAD2A, EFTUD2, YRDC, CDK6, VEGFA, BCL2L12, C16orf72, CCND2, SOWAHC, FBXL18, UBE2V1, LUZP1, BAZ2A, SLC2A3, ABL2, PAG1, NAA25, TFAP2A, EIF2B2, DIAPH1, PPIL1, NR6A1, HDGF, ZNF622, ACTR2, CHAC1, HSPA8, CD274, AVL9, MAFK, PLAG1, TUBB2A, L2HGDH, IER2, GPRC5A, RCAN3, N4BP1 ,RAD23B, FOXK1, CCND1, AHNAK2, SIK1, E2F3, IPPK, MYO5A, FURIN, RTN4, TNFSF9, CACUL1, PRDM4, KPNA3, OSCAR, TSC22D2, CSNK1E, CBX4, CDCA4, RIMS3, VPS4A, CARM1, PDIA6, PAFAH1B2, PLEKHB2, TAF13, NCKAP1, ZNF460, NAPG, PIM1, SBNO1, HIGD1A, FCF1, HSPA1B, YWHAQ, ARHGDIA, CCNE1, AMOTL1, CRKL, AP5Z1, PPP2R5C, RPS6KB1, SNRPB2, RAB3IP, SEC24A, MOB4, PPP1R11, SSRP1, MCFD2, CCND3, HSPA4L, KIF23, ATP5G3, RUBCN, OSBPL3, TMEM189, ZNRF2, CANX, EFNB2, CALU, SKI, NUP50, CLIP4, C11orf24, WIPI2, RPL14, GNB1, CCNT1, ADORA3, GABARAPL1, AURKAIP1, FGFR4, EXT1, FBXL20, APP, BAG4, GRB2, CDC25A, REL, ZNF391, ORC4, PRKAR2A, CLEC2D, TRIM35, BTRC, CLUH, MINK1, RACGAP1, ARIH1, USP42, MAP3K7, TM7SF3, KPNA1, SERBP1, CMTM4, HIST2H2BE, NUFIP2, PDCD1, RIF1, ZBTB34, TMEM245, CD180, GPR180, HOXA3, TBPL1, CRIM1, HNRNPA2B1, TARBP2, ASCC1, CLSPN, DDX3X, MAP2K3, SNCG, MSANTD4, TLL1, UBE2Q1, YIPF6, FZD9, BIRC5, UBE2H, DYNLL2, ITGA2, SLCO3A1, DICER1, HNRNPA1L2, TAOK1, CAMSAP1, CRK,SLC29A1, ALDH3B1, FAM103A1, LAMC1, CASKIN1, RPRD1B, AKR1B10, CEP55, CD2AP, MTHFR, DCTN5, GALNT1, ZNF284, SZRD1, JPT2, SMAD7, PAK2, IVNS1ABP, KIF5B, PHKA1, PLRG1, SRSF1, DCAF17, STRADB, LRIG2, PHLPP2, DMPK, FZD6, ZDHHC16, KIAA0895, KMT2D, CBX2, LAMP2, PNPLA6, RUNX2, SETD1B, RASEF, GPATCH8, PISD, SEC61A1, SOCS5, SRPRA, TLK1, NOTCH2, HNRNPA1, SLC39A9, TOB2, CDK4, DNAJC10, ODF2L, SNTB2, ATG14, CDK17, EIF1AX, PSAT1, UBR3, CHEK1, SHOC2, AP2B1, VAV2, MAP4K2, ZNRF3, FKBP1A, HOXC8, CARD10, SMAD3, GNG12, TMEM161B, RAPH1, CDC37L1, CDC42SE2, PLEKHA1, PDE4D, RNF168, GPR27, RS1, ARHGAP32, ASH1L, SMURF1, YWHAH, ZBTB10, ATAD5, LRRFIP2, SSU72, RNPS1, DYRK3, ZNF585B, WNT7A, B3GNT2, LSM11, POM121C, SYNRG, TM4SF1, ATG9A, RFK, MKX, MSL1, BTN3A3, USP48, AKT3, CBX6, GRAMD2B, CAPZA2, BSPRY, VSIR, MTMR3, ZMAT3, C1orf226, KANK1, ATXN7L3B, GOSR1, RALGAPB, RECK, STK38, ZNF267, CPSF7, PHF19, ZCCHC3, E2F7, ELK4, HAUS3, PPP6R3, GABPA, RPRD2, CCNE2, PPIG, SRPRB, TTLL5, DLGAP3, DOCK11, OCRL, MBD4, CDK1, FLCN, NFIC, CUL3, KLHL15, RAF1, CHMP4B, HOXA10, ZNF691, ENTPD1, PPM1A, PLPBP, ZFHX4, SNX16, CREBL2, CTDSPL, USP53, PRSS21, RBBP6, ZNF449, GNAL, EN2, VOPP1, TPM2, RAB23, HNRNPDL, THRAP3, TBRG1, TXNIP, PI4K2B, UBN2, YTHDC1, CASK, SESTD1, CDADC1, ZNF367, CPEB3, ACVR2A, DMRT2, CREBRF, POLDIP3, SLC25A12, AGO4, SIDT2, RAB15, ABHD2, HCFC2, OGT, ASGR2, PAGR1, CCDC88C, POLR2E, PRKAA1, WEE1, KIAA1456, ANKRD36, RASSF2, PPIP5K2, SYPL1, ARHGAP12, ZBTB16, CLU, PTPRD, RUNX1T1, LANCL1, C1orf21, LUC7L3, SH3BP4, WDR13, PNRC2, DECR1, ZNF704, C21orf62, SLC35E2B, PNISR, ZNF620, C3orf36, CNKSR3, SREK1, TNRC6B, WNK3, ADRA2B, ANAPC13, SPRED1, ACTR3B, CA8, ARMC12, NNT, CCDC80, FGF2, FLOT2, ZFP28, PHC3, PIK3R1, ZNRF1, AMOT, C16orf58, FAM122B, SCAMP4, DMTF1, EPM2AIP1, HEYL, PHYHIP, RBPJ, TRAK1, ZNF275, ABCC6, NUCKS1, PRICKLE2, LURAP1L, BCL2, DENND6A, FAM229B, CHIC1, SMDT1, MTFR1L, AXIN2, SALL1, NEGR1, PLPP3, RARB, TMEM100, TGFBR3, ANKMY1, SLC7A5, SLC25A22, OTUB1, LRPPRC, TPI1, MLXIP, COPB1, CCL4, TRAM1, B4GALT1, PSMB5, CAND1, HYOU1, RNH1, RELT, PCMT1, USP3, CSDE1, TM9SF2, RPS5, TUFT1, CDKN1A, HMGA1, C15orf39, SELENOI, KLC2, NOLC1, CDC27, LITAF, TMEM135, ARCN1, CDK8, C6orf106, BSG, PEX13, ACOX1, SNX11, PTPRJ, TRIP10, KIAA0100, YAP1, RNMT, SRPK1, CDKN2A, IPNL, KRT7, CHUK, SPTLC1, CDV3, TMC6, CPEB2, UBE2Q2, CPNE1, NKD1, TMEM138, PSKH1, RNF41, ENTPD6, PANK1, TADA2B, HMBOX1, AP3M1, UBE4A, BCL7A, CREG1, TFB1M, GANAB, ENTPD7, EDC3, SIRT4, TBC1D14, CUL2, ZBTB5, MBD1, ATP13A3, RCOR1, RPL36, ARL2, SPTBN1, UBE3C, BHLHE40, LRIF1, NCOR2, TUBB, KLHDC10, MTMR4, SUN1, RPL10, BCL2L2, BCL2L11, CDS2, PAFAH1B1, CYB561A3, PNPO, SUPT16H, POLE4, ANKRD13B, TMEM69, CAB39, GGA3, RHOV, RET, SLC9A1, LRRC57, PURA, NR2C2, TET3, AFF4, TBC1D20, DPP8, MIGA1, IFT74, PPP6C, MYB, IRAK1BP1, RASSF5, KIF21A, ZNF91, DSCR3, VPS33B, ABCB7, NOL4L, TCF3, PHACTR2, MAPKAPK2, KIF3B, PIP4P1, ASXL1, MCM3AP-AS1, STX17, MLLT6, ETNK1, AGO1, CYLD, ZBTB33, GABARAP, RNF38, AKAP11, RFWD2, TMEM109, MRPL40, PGD, RAB11FIP2, PRKCD, RAP2C, TIMM13, PEX12, KATNAL1, SCAMP5, GSK3B, INSR, MED11, FRYL, RPS6KA3, ETFRF1, MIB1, IRF4, PRRC2C, SLC9A6, TECPR2, USP31, TBL1XR1, TASP1, AGER, REXO1, ALOX12, TKTL1, CDC42, DNAJC9, VCL, CCNT2, KDR, RASGEF1B, CHMP3, PDCD4, JAK2, SH3BGRL2, U2SURP, BTG2, AMER1, C2orf42, TAB3, TLE4, TBCCD1, CBFA2T3, ITPR1, STXBP3, MECP2, CGNL1, EZH1, RNF138, ZNF280C, PDIK1L, RAB40B, SLC25A29, POU2AF1, ELN, MAP2K1, ANLN, IGF1R, ESRRA, KCNN4, HIST1H3H, TCEA1, EEF1A1, MTOR, TWIST1, MACC1, BRD1, DESI2, TPM1, EIF4E, PANK2, PBX3, IKBKB, ALDH9A1* |
| **miR-497 and miR-195 Common target genes**  **(Total 430)** | *DNAJA1, FASN, BZW1, USP15, AGO2, RNF149, JARID2, TPM3, CYP26B1, GATAD2A, EFTUD, YRDC, CDK6, VEGFA, BCL2L12, C16ORF72, CCND2, SOWAHC, FBXL18, UBE2V1, LUZP1, BAZ2A, SLC2A3, ABL2, PAG1, NAA25, TFAP2A, EIF2B2, DIAPH1, PPIL1, NR6A1, HDGF, ZNF622, ACTR2, CHAC1, HSPA8, CD274, AVL9, MAFK, PLAG1, TUBB2A, L2HGDH, IER2, GPRC5A, RCAN3, N4BP1, RAD23B, FOXK1, CCND1, AHNAK2, SIK1, E2F3, IPPK, MYO5A, FURIN, RTN4, TNFSF9, CACUL1, PRDM4, KPNA3, OSCAR, TSC22D2, CSNK1E, CBX4, CDCA4, RIMS3, VPS4A, CARM1, PDIA6, PAFAH1B2, PLEKHB2, TAF13, NCKAP1, ZNF460, NAPG, PIM1, SBNO1, HIGD1A, FCF1, HSPA1B, YWHAQ, ARHGDIA, CCNE1, AMOTL1, CRKL, AP5Z1, PPP2R5C, RPS6KB1, SNRPB2, RAB3IP, SEC24A, MOB4, PPP1R11, SSRP1, MCFD2, CCND3, HSPA4L, KIF23, ATP5G3, RUBCN, OSBPL3, TMEM189, ZNRF2, CANX, EFNB2, CALU, SKI, NUP50, CLIP4, C11ORF24, WIPI2, RPL14, GNB1, CCNT1, ADORA3, GABARAPL1, AURKAIP1, FGFR4, EXT1, FBXL20, APP, BAG4, GRB2, CDC25A, REL, ZNF391, ORC4, PRKAR2A, CLEC2D, TRIM35, BTRC, CLUH, MINK1, RACGAP1, ARIH1, USP42, MAP3K7, TM7SF3, KPNA1, SERBP1, CMTM4, HIST2H2BE, NUFIP2, PDCD1, RIF1, ZBTB34, TMEM245, CD180, GPR180, HOXA3, TBPL1, CRIM1, HNRNPA2B1, TARBP2, ASCC1, CLSPN, DDX3X, MAP2K3, SNCG, MSANTD4, TLL1, UBE2Q1, YIPF6, FZD9, BIRC5, UBE2H, DYNLL2, ITGA2, SLCO3A1, DICER1, HNRNPA1L2, TAOK1, CAMSAP1, CRK, SLC29A1, ALDH3B1, FAM103A1, LAMC1, CASKIN1, RPRD1B, AKR1B10, CEP55, CD2AP, MTHFR, DCTN5, GALNT1, ZNF284, SZRD1, JPT2, SMAD7, PAK2, IVNS1ABP, KIF5B, PHKA1, PLRG1, SRSF1, DCAF17, STRADB, LRIG2, PHLPP2, DMPK, FZD6, ZDHHC16, KIAA0895, KMT2D, CBX2, LAMP2, PNPLA6, RUNX2, SETD1B, RASEF, GPATCH8, PISD, SEC61A1, SOCS5, SRPRA, TLK1, NOTCH2, HNRNPA1, SLC39A9, TOB2, CDK4, DNAJC10, ODF2L, SNTB2, ATG14, CDK17, EIF1AX, PSAT1, UBR3, CHEK1, SHOC2, AP2B1, VAV2, MAP4K2, ZNRF3, FKBP1A, HOXC8, CARD10, SMAD3, GNG12, TMEM161B, RAPH1, CDC37L1, CDC42SE2, PLEKHA1, PDE4D, RNF168, GPR27, RS1, ARHGAP32, ASH1L, SMURF1, YWHAH, ZBTB10, ATAD5, LRRFIP2, SSU72, RNPS1, DYRK3, ZNF585B, WNT7A, B3GNT2, LSM11, POM121C, SYNRG, TM4SF1, ATG9A, RFK, MKX, MSL1, BTN3A3, USP48, AKT3, CBX6, GRAMD2B, CAPZA2, BSPRY, VSIR, MTMR3, ZMAT3, C1ORF226, KANK1, ATXN7L3B, GOSR1, RALGAPB, RECK, STK38, ZNF267, CPSF7, PHF19, ZCCHC3, E2F7, ELK4, HAUS3, PPP6R3, GABPA, RPRD2, CCNE2, PPIG, SRPRB, TTLL5, DLGAP3, DOCK11, OCRL, MBD4, CDK1, FLCN, NFIC, CUL3, KLHL15, RAF1, CHMP4B, HOXA10, ZNF691, ENTPD1, PPM1A, PLPBP, ZFHX4, SNX16, CREBL2, CTDSPL, USP53, PRSS21, RBBP6, ZNF449, GNAL, EN2, VOPP1, TPM2, RAB23, HNRNPDL, THRAP3, TBRG1, TXNIP, PI4K2B, UBN2, YTHDC1, CASK, SESTD1, CDADC1, ZNF367, CPEB3, ACVR2A, DMRT2, CREBRF, POLDIP3, SLC25A12, AGO4, SIDT2, RAB15, ABHD2, HCFC2, OGT, ASGR2, PAGR1, CCDC88C, POLR2E, PRKAA1, WEE1, KIAA1456, ANKRD36, RASSF2, PPIP5K2, SYPL1, ARHGAP12, ZBTB16, CLU, PTPRD, RUNX1T1, LANCL1, C1ORF21, LUC7L3, SH3BP4, WDR13, PNRC2, DECR1, ZNF704, C21ORF62, SLC35E2B, PNISR, ZNF620, C3ORF36, CNKSR3, SREK1, TNRC6B, WNK3, ADRA2B, ANAPC13, SPRED1, ACTR3B, CA8, ARMC12, NNT, CCDC80, FGF2, FLOT2, ZFP28, PHC3, PIK3R1, ZNRF1, AMOT, C16ORF58, FAM122B, SCAMP4, DMTF1, EPM2AIP1, HEYL, PHYHIP, RBPJ, TRAK1, ZNF275, ABCC6, NUCKS1, PRICKLE2, LURAP1L, BCL2, DENND6A, FAM229B, CHIC1, SMDT1, MTFR1L, AXIN2, SALL1, NEGR1, PLPP3, RARB, TMEM100, TGFBR3, ANKMY1* |
| **Other miR-497 target genes not involved in CC (Total 19)** | *MAP2K1, ANLN, IGF1R, ESRRA, KCNN4, HIST1H3H, TCEA1, EEF1A1, MTOR, TWIST1, MACC1, BRD1, DESI2, TPM1, EIF4E, PANK2, PBX3, IKBKB, ALDH9A1* |
| **Other miR-195 target genes not involved in CC (Total 197)** | *SLC7A5, SLC25A22, OTUB1, LRPPRC, TPI1, MLXIP, COPB1, CCL4, TRAM1, B4GALT1, PSMB, CAND1, HYOU1, RNH1, RELT, PCMT1, USP3, CSDE1, TM9SF2, RPS5, TUFT1, CDKN1A, HMGA1, C15orf39, SELENOI, KLC2, NOLC1, CDC27, LITAF, TMEM135, ARCN1, CDK8, C6orf106, BSG, PEX13, ACOX1, SNX11, PTPRJ, TRIP10, KIAA0100, YAP1, RNMT, SRPK1, CDKN2A, IPNL, KRT7, CHUK, SPTLC1, CDV3, TMC6, CPEB2, UBE2Q2, CPNE1, NKD1, TMEM138, PSKH1, RNF41, ENTPD6, PANK1, TADA2B, HMBOX1, AP3M1, UBE4A, BCL7A, CREG1, TFB1M, GANAB, ENTPD7, EDC3, SIRT4, TBC1D14, CUL2, ZBTB5, MBD1, ATP13A3, RCOR1, RPL36, ARL2, SPTBN1, UBE3C, BHLHE40, LRIF1, NCOR2, TUBB, KLHDC10, MTMR4, SUN1, RPL10, BCL2L2, BCL2L11, CDS2, PAFAH1B1, CYB561A3, PNPO, SUPT16H, POLE4, ANKRD13B, TMEM69, CAB39, GGA3, RHOV, RET, SLC9A1, LRRC57, PURA, NR2C2, TET3, AFF4, TBC1D20, DPP8, MIGA1, IFT74, PPP6C, MYB, IRAK1BP1, RASSF5, KIF21A, ZNF91, DSCR3, VPS33B, ABCB7, NOL4L, TCF3, PHACTR2, MAPKAPK2, KIF3B, PIP4P1, ASXL1, MCM3AP-AS1, STX17, MLLT6, ETNK1, AGO1, CYLD, ZBTB33, GABARAP, RNF38, AKAP11, RFWD2, TMEM109, MRPL40, PGD, RAB11FIP2, PRKCD, RAP2C, TIMM13, PEX12, KATNAL1, SCAMP5, GSK3B, INSR, MED11, FRYL, RPS6KA3, ETFRF1, MIB1, IRF4, PRRC2C, SLC9A6, TECPR2, USP31, TBL1XR1, TASP1, AGER, REXO1, ALOX12, TKTL1, CDC42, DNAJC9, VCL, CCNT2, KDR, RASGEF1B, CHMP3, PDCD4, JAK2, SH3BGRL2, U2SURP, BTG2, AMER1, C2orf42, TAB3, TLE4, TBCCD1, CBFA2T3, ITPR1, STXBP3, MECP2, CGNL1, EZH1, RNF138, ZNF280C, PDIK1L, RAB40B, SLC25A29, POU2AF1, ELN* |
