# Supplementary material for: Analysis of miR-497/195 cluster identifies new therapeutic targets in cervical cancer
Source: BMC Res Notes. 2024 Aug 2;17:217. doi: 10.1186/s13104-024-06876-8 (PMC11297691; doi:10.1186/s13104-024-06876-8)
Supplement: Supplementary file 13 — Additional file 13: Table 4. List of differentially expressed target genes. [file 13104_2024_6876_MOESM13_ESM.docx]

**Supplementary Table 4: List of differentially expressed target genes**

| **Differential Expression** | **Genes** |
| --- | --- |
| **Differentially expressed target genes (DETGs)** | *CDC25A, CCNE1, CHEK1, OSBPL3, RACGAP1, HSPA4L, KIF23, CDCA4, PSAT1, CEP55, CDK1, E2F7, CCNE2, GRAMD2B, RCAN3, TFAP2A, CLSPN, RASEF, TPM2, RECK, CLU, HOXA10, AKT3, ZBTB16, PTPRD, RAB23, PHYHIP, FAM229B, SALL1, PRICKLE2, AXIN2, BCL2, TXNIP, FGF2, CCDC80, RUNX1T1, HEYL, PLPP3, NEGR1, TMEM100, RELT, TUFT1, C6orf106, SLC29A2, ENTPD7, TET3, MYB, ATD5, PGD, RAB11f1P2, KDR, ITPR1, CGNL1, NR6A1, ANLN, HIST1H3H, ZNF367, TGFBK3, TPM1, RAB23* |
| **miRNA 195-5p** | **Upregulated**- *TFAP2A, NR6A1, RCAN3, ANLN, CDCA4, CCNE1, HSPA4L, KIF23, OSBPL3, HIST1H3H, CDC25A, RACGAP1, CHEK1, PSAT1, GRAMD2B, CLSPN, CEP55, E2F7, CCNE2, CDK1, ZNF367, RASEF* |
|  | **Downregulated-** *PRICKLE2, BCL2, FAM229B, AXIN2, SALL1, NEGR1, PLPP3, TMEM100, TGFBK3, AKT3, RECK, TPM1, HOXA10, TPM2, RAB23, ZBTB16, CLU, PTPRD, CCDC80, PHYHIP, FGF2, HEYL, RAB23, SALL1, AXIN2, TXNIP, RUNX1T1* |
| **miR 497 – 5p** | **Upregulated**- *RELT, TUFT1, CDC25A, CCNE1, C6orf106, CHEK1, SLC29A2, ENTPD7, OSBPL3, RACGAP1, HSPA4L, KIF23, RASEF, CDCA4, TET3, MYB, PSAT1, ATD5, PGD, CEP55, CDK1, E2F7, CCNE2, GRAMD2B, RCAN3, TFAP2, CLSPN* |
|  | **Downregulated-** *TPM2, RECK, CLU, RAB11F1P2, HOXA10, AKT3, ZBTB16, KDR, PTPRD, RAB23, PHYHIP, FAM229B, SALL1, ITPR1, PRICKLE2, AXIN2, BCL2, TXNIP, FGF2, CCDC80, CGNL1, RUNX1T1, HEYL, PLPP3, NEGR1, TMEM100* |
| **Common gene targets for miR-497/195 cluster** | **Upregulated**- *CDC25A, CCNE1, CHEK1, OSBPL3, RACGAP1, HSPA4L, KIF23, CDCA4, PSAT1, CEP55, CDK1, E2F7, CCNE2, GRAMD2B, RCAN3, TFAP2A, CLSPN, RASEF* |
|  | **Downregulated-** *TPM2, RECK, CLU, HOXA10, AKT3, ZBTB16, PTPRD, RAB23, PHYHIP, FAM229B, SALL1, PRICKLE2, AXIN2, BCL2, TXNIP, FGF2, CCDC80, RUNX1T1, HEYL, PLPP3, NEGR1, TMEM100* |
