# Supplementary material for: Analysis of miR-497/195 cluster identifies new therapeutic targets in cervical cancer
Source: BMC Res Notes. 2024 Aug 2;17:217. doi: 10.1186/s13104-024-06876-8 (PMC11297691; doi:10.1186/s13104-024-06876-8)
Supplement: Supplementary file 14 — Additional file 14: Table 5. The differentially expressed target genes of miR-497/195 cluster and their characteristic metastatic signatures. [file 13104_2024_6876_MOESM14_ESM.docx]

**Supplementary table 5: The Differentially Expressed target genes of miR-497/195 MC and their characteristic metastatic signatures**

| **Metastatic genes** | **Secondary sites** | **Expression** |
| --- | --- | --- |
| TFAP2A | head & neck,lung,other | Upregulated |
| CLSPN | head & neck,lung,other | Upregulated |
| RASEF | head & neck,lung,other | Upregulated |
| HIST1H3H | head & neck,lung,other | Upregulated |
| AKT3 | head & neck,lung,other | Downregulated |
| ITPR1 | head & neck,lung,other | Downregulated |
