# Supplementary material for: Analysis of miR-497/195 cluster identifies new therapeutic targets in cervical cancer
Source: BMC Res Notes. 2024 Aug 2;17:217. doi: 10.1186/s13104-024-06876-8 (PMC11297691; doi:10.1186/s13104-024-06876-8)
Supplement: Supplementary file 16 — Additional file 16: Table 7. Hub Genes and Immune Infiltrates. [file 13104_2024_6876_MOESM16_ESM.docx]

**Supplementary Table 7: Hub genes and its Immune Infiltrates**

| **Hub Genes** | **Immune Infiltrates** |
| --- | --- |
| **CCNE1** | Plasma B cell, cancer associated fibroblasts, common myeloid progenitor, neutrophils, activated NK cells and T cell CD4+ effector memory |
| **CCNE2** | Memory B cell, Plasma B cell, cancer associated fibroblasts, endothelial cells, hematopoietic stem cells, macrophages, resting mast cells, Neutrophils, Activated NK cells, Plasmacytoid dendritic cells, T cell CD4+ (non-regulatory, T cell CD4+ effector memory, T cell CD4+ memory resting, T cell CD8+ effector memory, T cell follicular helper and T cell regulatory (Tregs) |
| **ANLN** | Memory B cell, cancer associated fibroblasts, endothelial cells, hematopoietic stem cells, macrophages, resting mast cells, Myeloid dendritic cell activated, T cell CD4+ effector memory, Neutrophils, Activated NK cells, T cell CD4+ naïve, T cell CD4+ Th1, T cell CD8+ central memory, T cell CD8+ effector memory and T cell regulatory (Tregs) |
| **RACGAP1** | Memory B cell, cancer associated fibroblasts, endothelial cells, macrophages, Myeloid dendritic cell resting, T cell CD4+ effector memory, Neutrophils, Activated NK cells, Plasmacytoid dendritic cells, T cell CD4+ Th1, T cell CD8+ effector memory and myeloid derived suppressor cells (MDSC) |
| **KIF23** | Memory B cell, cancer associated fibroblasts, endothelial cells, macrophages, Myeloid dendritic cell activated, neutrophils, class-switch B cell, monocytes, plasmacytoid dendritic cells, T cell CD4+ memory cell, T cell CD4+ naïve, T cell CD4+ Th1, T cell CD8+ effector memory, T cell gamma delta, T cell NK and T cell CD8+ central memory |
| **CHEK1** | Memory B cell, cancer associated fibroblasts, endothelial cells, macrophages M2, Myeloid dendritic cell, neutrophils, class-switch B cell, plasmacytoid dendritic cells, T cell CD4+ naïve, NK cell, T cell NK, T cell CD8+ naive and Common Lymphoid progenitor |
| **CDC25A** | Common Lymphoid progenitor, endothelial cells, macrophages M2, Myeloid dendritic activated cell, macrophages M2, NK cell, mast cell activated, monocytes and T cell CD8+ naive |
| **E2F7** | Plasma B cells, B cells, cancer associated fibroblasts, endothelial cells, hematopoetic stem cells, macrophages, mavrophages M1 and M2, mast cells activated, MDSC, monocytes myeloid dendritic activated, myeloid dendritic resting, neutrophils, T cell CD4+ (non-regulatory), T cell CD4+ memory resting, T cell CD4+ naive, T cell CD4+ Th1, T cell CD4+ Th2, T cell CD8+ effector memory, T cell CD8+, T cell follicular helper and T cell reg (Treg) |
| **CDK1** | B cell memory, B cell naïve, B cell plasma, Endothelial cells, hematopoetic stem cell, macrophages m1, macrophages M2, mast cells activated, mast cells resting, MDSC, monocytes, myeloid dendritic cells, myeloid dendritic cells resting, neutrophils, NK cells activated, plasmacytoid dendritic cells, T cells CD4+1 (non-regulatory), T cell CD4+ Th1, T cell CD8+ and T cell follicular helper cell |
| **CEP55** | B cell memory, B cell, Cancer associated fibroblasts, macrophages, MDSC, Myeloid dendritic cell activated, neutrophils, NK call, plasmacytoid dendritic cells, T cell CD4+ resting, T cell CD4+ naive, T cell CD4+ Th1, T cell CD8+ central memory, T cell CD8+ effector memory, T cell CD8+, T cell gamma delta and T cell regulatory (Treg) |
