# Supplementary material for: Analysis of miR-497/195 cluster identifies new therapeutic targets in cervical cancer
Source: BMC Res Notes. 2024 Aug 2;17:217. doi: 10.1186/s13104-024-06876-8 (PMC11297691; doi:10.1186/s13104-024-06876-8)
Supplement: Supplementary file 17 — Additional file 17: Table 8. The list of drugs and interacting genes. [file 13104_2024_6876_MOESM17_ESM.docx]

**Supplementary table 8: The list of drugs and interacting genes**

| Gene | Drug | Interaction types | Sources | pmids | Interaction Score |
| --- | --- | --- | --- | --- | --- |
| KDR | SUNITINIB | Inhibitory | TALC, DTC, MyCancerGenome, TdgClinicalTrial, JAX-CKB, TEND, CIViC, Guide to Pharmacology, My Cancer Genome, Clinical Trial, PharmGKB, TTD | 27149458, 20142593, 25639617, 1924897, 16418310, 23583911, 16533791, 218852, 21963305,  20124951, 12538485, 1475371, 11752352, 2801162, 19723655, 12873999 | 0.29 |
| KDR | PAZOPANIB | Inhibitory | TALC, DTC, My Cancer Genome,  Tdg Clinical Trial, CIViC, Guide To Pharmacology, My Cancer Genome, Clinical Trial PharmGKB | 26063633, 27160228, 17288876, 2541116, 24036042 | 0.14 |
| KDR | SORAFANIB | Antagonistic/  Inhibitory | TALC, DTC,  My Cancer Genome, Tdg Clinical Trial, Clearity Foundation Clinical Trial, JAX | 16824050, 16418310, 26344591, 2518270, 16503817, 16757355, 20124951, 2574321, 28362716, 16446323, 25816720, 1972365, 24510746, 20630084 | 0.14 |
| KDR | PONATANIB | Inhibitory | MY Cancer Genome | - | 0.03 |
| KDR | GEMCITABINE | - | JAX-CKB | - | 0.01 |
| KDR | DASATANIB | - | DTC | - | 0.01 |
| MYB | DOXORUBICIN | - | NCI | 15001837 | 0.45 |
| MYB | PACLITAXEL | - | NCI | 15001837 | 0.44 |
| MYB | FLUOROURACIL | - | NCI | 15001837 | 0.41 |
| CCNE1 | PALBOCICLIB | - | CiViC | 25557169, 27020857 30807234 | 1.09 |
| CHEK1 | ETOPOSIDE | - | DTC | 22364746 | 0.07 |
| CHEK1 | GEMCITABINE | - | NCI | 17245119 | 0.04 |
| CHEK1 | OLAPARIB | - | CiViC | 28490518 | 0.12 |
| CHEK1 | PALBOCICLIB | - | DTC | - | 0.04 |
| CHEK1 | CISPLATIN | - | CIViC | 28490518 | 0.02 |
| RACGAP1 | MITOXANTRONE | - | DTC | - | 0.02 |
| AKT3 | EVEROLIMUS | Inhibitory | My Cancer Genome | - | 0.07 |
| BCL2 | RALTITREXED | - | NCI | 10785598 | 0.41 |
| BCL2 | TENIPOSIDE | - | NCI | 9842975 | 0.36 |
| BCL2 | PACLITAXEL | Inhibitory | PharmGKB | 16741658, 16895478, 23963862, 1723052, 17119350, 17062688 | 0.16 |
| BCL2 | METHYLPRESDISOLONE | - | NCI | 16141680, 16684279 | 0.27 |
| BCL2 | FLOXURIDINE | - | NCI | 8324744 | 0.21 |
| BCL2 | ETIPOSIDE | - | NCI | 11350408, 14977850, | 0.08 |
| BCL2 | DOCETAXEL | - | PharmGKB | 17674353, 15277270, 15161985, 1564350, 15714982, 23963862, 15685445 | 0.17 |
| BCL2 | MITOXANTRANE | - | NCI | 11751483 | 0.07 |
| BCL2 | OXALIPLATIN | - | NCI | 114977850 | 0.12 |
| BCL2 | EPIRUBICIN | - | NCI | 14503796 | 0.1 |
| BCL2 | TRETINOIN | - | NCI | 9402847 | 0.09 |
| BCL2 | CISPLATIN | - | NCI | 12543810, 11350408 | 0.04 |
| BCL2 | STREPTOZOCIN | - | NCI | 16741044 | 0.06 |
| BCL2 | DOXORUBICIN | - | NCI | 16749867 | 0.03 |
| BCL2 | CARBOPLATIN | - | PharmGKB | 23963862 | 0.06 |
| BCL2 | VINCRISTINE | - | PharmGKB | - | 0.02 |
| BCL2 | BORTEZOMIB | Inhibitory | TALC | - | 0.02 |
| FGF2 | LINALINOMIDE | - | PharmGKB | 28373444 | 0.43 |
| FGF2 | VINCRISTRINE | - | NCI | 8988045 | 0.1 |
| FGF2 | THALIDOMIDE | - | PharmGKB | 28373444 | 0.17 |
| FGF2 | SIROLIMUS | Other/unknown | Tdg Clinical trial | 12742462 | 0.11 |
